# Supplementary material for: Psychological correlates of free colorectal cancer screening uptake in a Scottish sample: a cross-sectional observational study
Source: BMJ Open. 2022 Feb 1;12(2):e042210. doi: 10.1136/bmjopen-2020-042210 (PMC8808413; doi:10.1136/bmjopen-2020-042210)
Supplement: Supplementary data [file bmjopen-2020-042210supp001.pdf]

Supplementary Materials for: Fawns-Ritchie et al. Psychological correlates of free colorectal cancer screening uptake in a Scottish sample

HAGIS risk aversion questions

1. Income A, which will with certainty give you a £1,500 per month for the rest of your life OR Income B which will give you a 50-50 chance of £3,000 and a 50-50 chance of £1,000 per month for the rest of your life.

Would you choose Income A or Income B?
(a) Income A
(b) Income B

2. Income A, which will with certainty give you a £1,500 per month for the rest of your life OR Income B, which will give you a 50-50 chance of £3,000 and a 50-50 chance of £1,200 per month for the rest of your life.

Would you choose Income A or Income B?
(a) Income A
(b) Income B

3. Income A, which will with certainty give you a £1,500 per month for the rest of your life OR Income B, which will give you a 50-50 chance of £3,000 and a 50-50 chance of £1,300 per month for the rest of your life.

Would you choose Income A or Income B?
(a) Income A
(b) Income B

Fawns-Ritchie C, *et al. BMJ Open* 2022; 12:e042210. doi: 10.1136/bmjopen-2020-042210

HAGIS time orientation questions

1. Would you rather have £1,500 now or £1,506 a month from now?
   (a) £1,500 now
   (b) £1,506 a month from now

2. Would you rather have £1,500 now or £1,512 a month from now?
   (a) £1,500 now
   (b) £1,512 a month from now

3. Would you rather have £1,500 now or £1,518 a month from now?
   (a) £1,500 now
   (b) £1,518 a month from now

4. Would you rather have £1,500 now or £1,524 a month from now?
   (a) £1,500 now
   (b) £1,524 a month from now

5. Would you rather have £1,500 now or £1,536 a month from now?
   (a) £1,500 now
   (b) £1,536 a month from now

6. Would you rather have £1,500 now or £1,548 a month from now?
   (a) £1,500 now
   (b) £1,548 a month from now

7. Would you rather have £1,500 now or £1,596 a month from now?
   (a) £1,500 now
   (b) £1,596 a month from now

Fawns-Ritchie C, *et al. BMJ Open* 2022; 12:e042210. doi: 10.1136/bmjopen-2020-042210

SUPPLEMENTARY METHODS

Cognitive ability

Five tests of cognitive function that were assessed during the face-to-face interview were used here. Verbal declarative memory was assessed using the word list learning test in which participants were presented with a list of 10 words and had to recall these words immediately, and again after an approximately 20 minute delay. The scores for word list learning immediate and delayed tests correlated at r=0.66 ($p<0.001$) therefore, the score used here is the total number of words recalled across the two tests (range 0-20). Categorical (animal) fluency, in which participants are asked to name as many animals as possible, was used to measure executive function. The score is the number of animals named in 60 seconds. The word list learning and animal fluency tests have been administered in other longitudinal studies of ageing including the Health and Retirement Study[1] and the English Longitudinal Study of Ageing.[2] Processing speed was measured using the letter digit substitution test.[3] Here, participants were presented with a piece of paper with a key pairing letters with numbers. Below the key were rows of letters with empty boxes underneath each letter. Participants were to use this key to write in the number that is paired with each letter. The score is the number of correct letter-digit matches made in 60 seconds. Two tests from the COGNITO neuropsychological test battery were administered to assess vocabulary and non-verbal fluid reasoning.[4] A shortened version of the COGNITO vocabulary test[4] was used. Participants were presented with a target word and were instructed to select which of six alternatives means the same as the target word. There were 20 items and the score is the number of correctly answered questions. COGNITO matrices[4] was used to assess non-verbal reasoning. Participants were presented with 15 matrix designs with a piece missing

Fawns-Ritchie C, *et al. BMJ Open* 2022; 12:e042210. doi: 10.1136/bmjopen-2020-042210

and they were required to select the missing piece from a list of alternatives. The score is the number of correctly answered questions in 4 minutes.

To create a measure of general cognitive ability, scores on the five cognitive tests were entered into a principal components analysis. The eigenvalues and scree plot (Supplementary Figure 1) indicated that one component should be extracted. The first unrotated principal component accounted for 44% of the total variance across the five tests. The loadings for the five tests were: total word recall=0.60, animal fluency=0.73, letter digit substitution=0.70, vocabulary=0.58, matrices=0.68. The score from the first unrotated principal component was used here as a measure of general cognitive ability (mean=0.00, SD=1.00).

Fawns-Ritchie C, *et al. BMJ Open* 2022; 12:e042210. doi: 10.1136/bmjopen-2020-042210

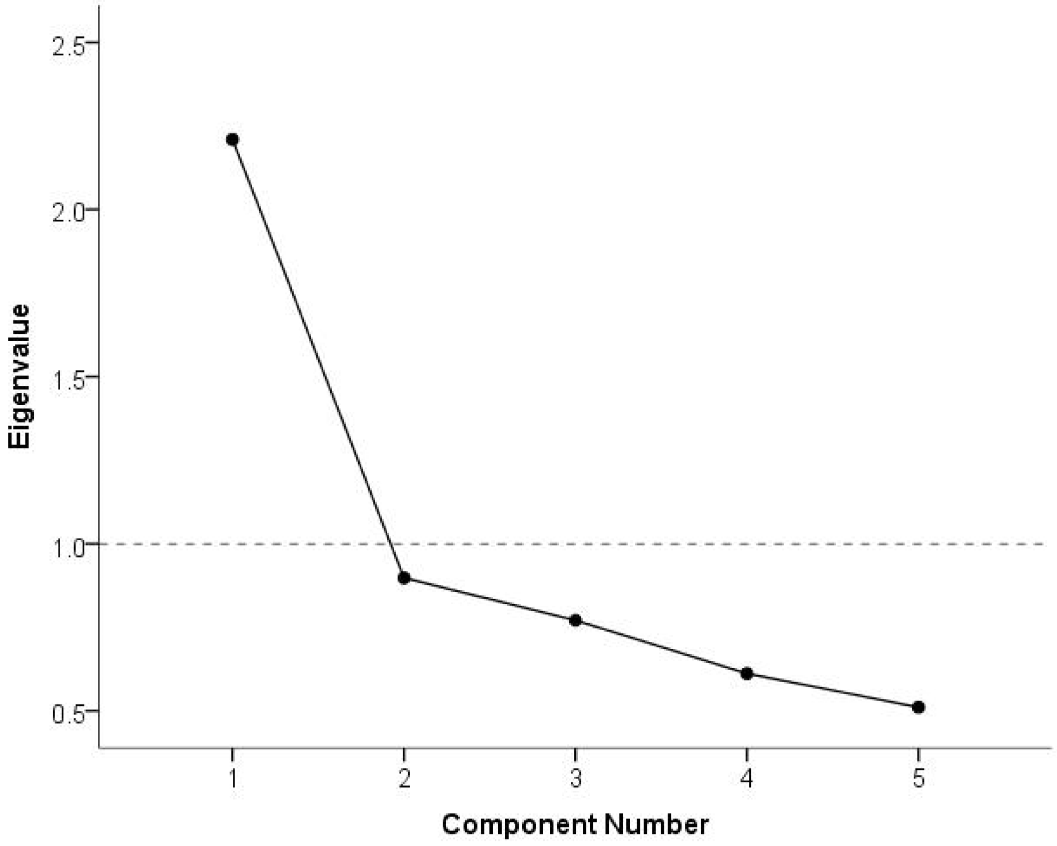

Supplementary Figure 1 Scree plot of eigenvalues from the 5 HAGIS cognitive tests

Supplementary Table 1 Pearson (below the diagonal) and rank-order (above the diagonal) correlations between psychological variables and covariates

	1	2	3	4	5	6	7	8	9	10	11	12	13	14
1. Age	-	0.04	-0.04	-0.16***	0.03	0.18***	-0.01	-0.20***	-0.28***	-0.03	-0.08	-0.05	0.06	-0.08
2. Sex	0.04	-	0.18***	0.07*	0.03	-0.08	-0.11*	-0.04	-0.04	-0.04	-0.29***	-0.09*	0.05	0.06
3. Cohabiting	-0.04	0.18***	-	0.14***	0.17***	-0.10*	-0.11*	0.17***	0.15***	0.11**	0.01	0.06	0.05	0.14**
4. Qualifications	-0.14***	0.08*	0.14***	-	0.35***	-0.21***	-0.24***	0.22***	0.48***	0.00	0.03	0.09*	0.13**	0.36***
5. SIMD	0.02	0.03	0.17***	0.35***	-	-0.15***	-0.28***	0.05	0.26***	0.01	0.00	-0.02	0.04	0.13**
6. Risk aversion	0.15***	-0.09*	-0.07	-0.16***	-0.12**	-	0.14**	-0.17***	-0.17***	-0.06	0.07	0.01	0.03	-0.18***
7. Present-orientation	-0.01	-0.10*	-0.11*	-0.25***	-0.30***	0.14**	-	-0.11*	-0.11***	0.07	0.10*	0.04	0.03	-0.12**
8. Health literacy	-0.21***	-0.06	0.16***	0.22***	0.08*	-0.12**	-0.09	-	0.45***	0.11**	0.08	0.13**	0.08	0.22
9. Cognitive ability	-0.27***	-0.04	0.15***	0.49***	0.27***	-0.13**	-0.11*	0.46***	-	0.17***	0.22***	0.23***	0.18***	0.39***
10. Extraversion	-0.02	-0.05	0.11**	-0.02	0.01	-0.05	0.07	0.11**	0.14**	-	0.37***	0.20***	0.27***	0.28***
11. Agreeableness	-0.09*	-0.30***	0.02	0.03	0.01	0.11*	0.10*	0.13**	0.22***	0.35***	-	0.45***	0.25***	0.27***
12. Conscientiousness	-0.05	-0.09*	0.07	0.09*	-0.01	0.05	0.04	0.15**	0.23***	0.18***	0.44***	-	0.30***	0.30***
13. Emotional stability	0.07	0.05	0.05	0.15***	0.06	0.03	0.01	0.08	0.18***	0.28***	0.22***	0.29***	-	0.08
14. Intellect	-0.06	0.08	0.12**	0.39***	0.14**	-0.15***	-0.13**	0.26***	0.38***	0.27***	0.25	0.30***	0.08	-

SIMD, Scottish Index of Multiple Deprivation.
Sex is coded 0 for female, 1 for male. Cohabiting is coded 0 for living alone, 1 for cohabiting.
*p<0.05, **p<0.01, ***p<0.001.

Supplementary Table 2 Partial correlations controlling for age and sex between psychological variables

	1	2	3	4	5	6	7	8	9
1. Risk aversion	-								
2. Present-orientation	0.15**	-							
3. Health literacy	-0.18***	-0.11*	-						
4. Cognitive ability	-0.12*	-0.16**	0.44***	-					
5. Extraversion	-0.01	0.12*	0.06	0.14**	-				
6. Agreeableness	0.09	0.09	-0.01	0.13*	0.37***	-			
7. Conscientiousness	0.05	0.06	0.06	0.20***	0.22***	0.45***	-		
8. Emotional stability	0.03	0.03	0.1	0.23***	0.27***	0.30***	0.33***	-	
9. Intellect	-0.15**	-0.13**	0.23***	0.37***	0.27***	0.27***	0.21***	0.09	-

Fawns-Ritchie C, *et al. BMJ Open* 2022; 12:e042210. doi: 10.1136/bmjopen-2020-042210

Supplementary Table 3 Participant characteristics according to whether they have complete data on all variables of interest (completers) or whether they had missing data (non-completers)

	N	Completers (n=384)	Non-completers (n=524)	p value for a difference
Age (years), mean (SD)	908	65.33 (8.10)	66.24 (8.31)	0.101
Sex, n (%)	908			0.953
Female		214 (55.7%)	291 (55.5%)	
Male		170 (44.3%)	233 (44.5%)	
Living Arrangement, n (%)	908			0.003
Cohabiting		275 (71.6%)	325 (62.0%)	
Living alone		109 (28.4%)	199 (38.0%)	
Qualifications, n (%)	902			<0.001
Primary or less		43 (11.2%)	124 (23.9%)	
O level/O grade		113 (29.4%)	162 (31.3%)	
Highers/Sixth year studies/HNC/HND		113 (29.4%)	148 (28.6%)	
First degree		62 (16.1%)	57 (11.0%)	
Postgraduate/higher degree		53 (13.8%)	27 (5.2%)	
SIMD, n (%)	908			0.007
1 (Most deprived)		68 (17.7%)	144 (27.5%)	
2		86 (22.4%)	109 (20.8%)	
3		121 (31.5%)	142 (27.1%)	
4 (Least deprived)		109 (28.4%)	129 (24.6%)	

SIMD, Scottish Index of Multiple Deprivation.

Fawns-Ritchie C, *et al. BMJ Open* 2022; 12:e042210. doi: 10.1136/bmjopen-2020-042210

Supplementary Table 4 Odd ratios (95% CIs) for participating in colorectal cancer screening using participants with complete data (n=384)

	Model 1	Model 2
	OR (95% CI)	OR (95% CI)
Risk aversion	0.51 (0.35 to 0.72)***	0.53 (0.37 to 0.77)**
Present-orientation	0.90 (0.82 to 0.98)*	0.96 (0.87 to 1.06)
Health literacy	1.17 (0.99 to 1.37)	1.10 (0.93 to 1.31)
Cognitive ability	1.56 (1.17 to 2.08)**	1.32 (0.96 to 1.83)
Extraversion	0.98 (0.94 to 1.01)	0.98 (0.95 to 1.02)
Agreeableness	0.97 (0.92 to 1.02)	0.98 (0.93 to 1.03)
Conscientiousness	1.02 (0.98 to 1.06)	1.02 (0.98 to 1.07)
Emotional stability	1.02 (0.98 to 1.06)	1.01 (0.98 to 1.05)
Intellect	1.05 (1.00 to 1.10)	1.02 (0.97 to 1.08)

Model 1 reports the ORs (95% CIs) for each psychological variable entered individually, adjusting for age, age-squared, sex, living arrangement and sex*living arrangement. Model 2 reports the ORs (95% CIs) when additionally adjusting for deprivation and qualifications. *p<0.05, **p<0.01, ***p<0.001.

SUPPLEMENTARY REFERENCES

1 Sonnega A, Faul JD, Ofstedal MB*, et al.* Cohort Profile: the Health and Retirement Study (HRS). *International journal of epidemiology* 2014;**43**:576-85. doi: 10.1093/ije/dyu067

2 Steptoe A, Breeze E, Banks J*, et al.* Cohort profile: the English longitudinal study of ageing. *International journal of epidemiology* 2013;**42**:1640-8. doi: 10.1093/ije/dys168

3 van der Elst W, van Boxtel MP, van Breukelen GJ*, et al.* The Letter Digit Substitution Test: normative data for 1,858 healthy participants aged 24–81 from the Maastricht Aging Study (MAAS): influence of age, education, and sex. *J Clin Exp Neuropsychol* 2006;**28**:998-1009. doi: 10.1080/13803390591004428

4 Ritchie K, de Roquefeuil G, Ritchie C*, et al.* COGNITO: computerized assessment of information processing. *J Psychol Psychother* 2014;**4**:136. doi: 10.4172/2161-0487.1000136
