# Supplementary material for: Psychological correlates of free colorectal cancer screening uptake in a Scottish sample: a cross-sectional observational study
Source: BMJ Open. 2022 Feb 1;12(2):e042210. doi: 10.1136/bmjopen-2020-042210 (PMC8808413; doi:10.1136/bmjopen-2020-042210)
Supplement: Author's manuscript [file bmjopen-2020-042210.draft_revisions.pdf]

BMJ Open is committed to open peer review. As part of this commitment we make the peer review history of every article we publish publicly available.

When an article is published we post the peer reviewers' comments and the authors' responses online. We also post the versions of the paper that were used during peer review. These are the versions that the peer review comments apply to.

The versions of the paper that follow are the versions that were submitted during the peer review process. They are not the versions of record or the final published versions. They should not be cited or distributed as the published version of this manuscript.

BMJ Open is an open access journal and the full, final, typeset and author-corrected version of record of the manuscript is available on our site with no access controls, subscription charges or pay-per-view fees (http://bmjopen.bmj.com).

If you have any questions on BMJ Open's open peer review process please email info.bmjopen@bmj.com

BMJ Open

Psychological correlates of free colorectal cancer screening uptake in a Scottish sample: A cross-sectional observational study

Journal:	*BMJ Open*
Manuscript ID	bmjopen-2020-042210
Article Type:	Original research
Date Submitted by the Author:	28-Jun-2020
Complete List of Authors:	Fawns-Ritchie, Chloe; The University of Edinburgh<br>Miller, Christopher; University of Stirling, Department of Psychology<br>van der Pol, Marjon; University of Aberdeen, Health Economics Research Unit<br>Douglas, Elaine; University of Stirling, Division of Economics<br>Bell, David; University of Stirling, Division of Economics<br>O'carroll, Ronan; University of Stirling, Division of Psychology<br>Deary, Ian; The University of Edinburgh, Department of Psychology
Keywords:	EPIDEMIOLOGY, PUBLIC HEALTH, STATISTICS & RESEARCH METHODS

SCHOLARONE™
Manuscripts

I, the Submitting Author has the right to grant and does grant on behalf of all authors of the Work (as defined in the below author licence), an exclusive licence and/or a non-exclusive licence for contributions from authors who are: i) UK Crown employees; ii) where BMJ has agreed a CC-BY licence shall apply, and/or iii) in accordance with the terms applicable for US Federal Government officers or employees acting as part of their official duties; on a worldwide, perpetual, irrevocable, royalty-free basis to BMJ Publishing Group Ltd ("BMJ") its licensees and where the relevant Journal is co-owned by BMJ to the co-owners of the Journal, to publish the Work in this journal and any other BMJ products and to exploit all rights, as set out in our licence.

The Submitting Author accepts and understands that any supply made under these terms is made by BMJ to the Submitting Author unless you are acting as an employee on behalf of your employer or a postgraduate student of an affiliated institution which is paying any applicable article publishing charge ("APC") for Open Access articles. Where the Submitting Author wishes to make the Work available on an Open Access basis (and intends to pay the relevant APC), the terms of reuse of such Open Access shall be governed by a Creative Commons licence – details of these licences and which Creative Commons licence will apply to this Work are set out in our licence referred to above.

Other than as permitted in any relevant BMJ Author's Self Archiving Policies, I confirm this Work has not been accepted for publication elsewhere, is not being considered for publication elsewhere and does not duplicate material already published. I confirm all authors consent to publication of this Work and authorise the granting of this licence.

For peer review only

Psychological correlates of free colorectal cancer screening uptake in a Scottish sample: A cross-sectional observational study

Chloe Fawns-Ritchie[1,2], Christopher B. Miller[3], Marjon van der Pol[4], Elaine Douglas[5], David Bell[5], Ronan E. O'Carroll[3]*, and Ian J. Deary[1,2]*

[1]Centre for Cognitive Ageing and Cognitive Epidemiology, University of Edinburgh, Edinburgh, UK

[2]Department of Psychology, University of Edinburgh, Edinburgh, UK

[3]Division of Psychology, University of Stirling, Stirling, UK

[4]Health Economics Research Unit, University of Aberdeen, Aberdeen, UK

[5]Division of Economics, University of Stirling, Stirling, UK

*These authors contributed equally

Corresponding author: Chloe Fawns-Ritchie, Department of Psychology, University of Edinburgh, Edinburgh, Scotland, UK, EH8 9JZ.

Telephone: (+44) 0131 650 8317

Email: c.fawns-ritchie@ed.ac.uk

Word count: 3035

For peer review only - http://bmjopen.bmj.com/site/about/guidelines.xhtml

For peer review only

ABSTRACT

Objectives Colorectal cancer (CRC) screening uptake in Scotland is 56%. This study examined whether psychological factors were associated with CRC screening uptake.

Design Cross-sectional observational study.

Setting This study used data from the Healthy AGeing In Scotland (HAGIS) pilot study, a study designed to be representative of Scottish adults aged 50 years and older.

Participants 908 (505 female) Scottish adults aged 50 to 81 years (mean age=65.84, SD=8.24), who took part in the HAGIS study (2017-2018).

Primary and secondary outcome measures Participants were asked about participation in CRC screening. Health literacy and cognitive function tests were administered. Participants completed questionnaires assessing risk aversion, time preference (e.g., more or less present-oriented), and personality traits.

Results Logistic regression was used to test whether risk aversion, time preference, health literacy, cognitive ability, and personality were associated with CRC screening. A one-point increase in risk aversion (OR=0.67, 95% CI 0.51 to 0.85), and present-orientation (OR=0.86, 0.80 to 0.94) was associated with reduced odds of screening. Higher scores on health literacy (OR per one-point increase=1.20, 1.09 to 1.31), the intellect personality trait (OR per one-point increase=1.05, 1.01 to 1.09), and cognitive ability (OR per SD increase=1.51, 1.25 to 1.81), were associated with increased odds of screening. Higher risk aversion was the only psychological variable that remained associated with CRC screening when all psychological variables and levels of deprivation and education were entered together. A backward elimination model retained risk aversion and cognitive ability as significant correlates of CRC screening.

For peer review only - http://bmjopen.bmj.com/site/about/guidelines.xhtml

Conclusion Individuals who are more risk averse are less likely to participate in free, home CRC screening. There was some indication that higher cognitive ability is associated with increased odds of participating in CRC screening.

Keywords: Cancer screening, cognitive ability, personality, health literacy, risk aversion, time preference

For peer review only

For peer review only - http://bmjopen.bmj.com/site/about/guidelines.xhtml

Strengths and limitations of this study

- This study used data from the Healthy AGeing In Scotland (HAGIS) pilot study, which is a study designed to be representative of Scottish adults aged 50 years and older.
- A wide range of psychological characteristics were measured which allowed us to investigate whether each psychological characteristic was associated with colorectal cancer screening independent of the other psychological characteristics measured.
- The outcome measure used was whether participants have ever completed a colorectal screening test, and we did not examine whether participants regularly take part in colorectal cancer screening.

For peer review only

For peer review only - http://bmjopen.bmj.com/site/about/guidelines.xhtml

INTRODUCTION

Colorectal cancer (CRC) is the third most common cancer in Scotland.[1] Screening to detect CRC using the faecal occult blood test (FOBT) has been found to reduce CRC-related mortality.[2] The Scottish Bowel Screening Programme was implemented in 2009 and involves inviting adults aged 50 to 74 years to take part in free home screening (FOBT kits are mailed to eligible individuals and returned postage free) for faecal blood every two years.[3] Uptake of CRC screening in Scotland is 56%.[3, 4] Understanding the characteristics of individuals who do not complete the FOBT test are important for designing methods for improving uptake. Lower age, male sex, and lower socioeconomic status are associated with lower screening rates.[4, 5]

Psychological factors might influence CRC screening uptake. Higher cognitive function and higher scores on personality traits including extraversion and conscientiousness have been associated with higher rates of cancer screening.[6-9] One study found that better cognitive function and higher conscientiousness, when measured in the same model, were associated with higher rates of participation in CRC after adjusting for age and sex.[10] These associations were attenuated and non-significant when additionally adjusting for health literacy and household wealth.[10] Adequate health literacy—the ability to obtain and understand basic health information[11]—has been identified as another possible correlate of CRC screening. Whereas some studies have found that lower health literacy was associated with reduced rates of CRC screening, others have not.[12-15] The association between health literacy and CRC screening was found to be attenuated by 40% when adjusting for cognitive function.[10]

Further psychological barriers to screening may include risk and time preference.[16-20] Individuals who are more future-oriented may be more likely to participate in routine cancer

For peer review only

For peer review only - http://bmjopen.bmj.com/site/about/guidelines.xhtml

screening because these individuals place a higher value on future benefits of screening, such as reducing the need for more invasive cancer treatment, and a lower value on the immediate costs of screening, such as time and effort, compared to individuals who are more present-oriented.[17, 18] Future-oriented women have been found to be more likely to undergo breast cancer and cervical screening;[17-19] however, more present-oriented men were found to be more likely to attend a prostate exam.[17] When examining the association between risk aversion and cancer screening, one study found that risk averse women were less likely to undergo regular breast cancer screening.[18] Another study[19] reported that risk aversion was weakly associated with reduced use of cancer screening services; however, these associations did not reach statistical significance. Risk averse individuals might be less likely to take part in cancer screening because screening may lead to having to undergo risky medical treatments.[18, 19]

Most studies have tended to examine the association between one psychological variable, or a small number of psychological variables, with cancer screening uptake. These psychological variables, however, are not independent of each other. Higher cognitive ability is moderately correlated with health literacy[21] and with greater future-orientation.[22] Risk aversion has been found to be associated with lower cognitive function and lower literacy.[23] Risk and time preferences also have small, but inconsistent, associations with conscientiousness, agreeableness and extraversion.[24] The unique contributions of these psychological variables with CRC screening uptake are not known. Using data from the Healthy AGeing in Scotland (HAGIS) pilot study—a study designed to be representative of middle-aged and older Scottish adults—the present study aimed to evaluate whether risk aversion, time preference, cognitive function, health literacy and personality traits, when examined concurrently in the same model, have independent associations with participation in home CRC screening.

For peer review only

For peer review only - http://bmjopen.bmj.com/site/about/guidelines.xhtml

METHODS

Participants

Data from the HAGIS pilot study[25] were used here. To recruit participants, a random sample of residual addresses in Scotland were identified. These addresses were screened to identify addresses where at least one resident was aged over 50 years. Eligible addresses were sent a study invitation. More information on the sampling procedure is reported elsewhere.[25] Eligible participants were those aged over 50 years, and their partners if their partner was aged over 45 years.[25] A total of 1,057 participants were recruited in 2016-2017. Ethical approval was obtained from the University of Stirling Ethics, the Administrative Data Research Network (PROJ-065-HAGIS Study), and the Public Benefits and Privacy Panel (HAGIS 1516-0417). This study conformed to the principles embodied in the Declaration of Helsinki.

Face-to-face interviews were carried out in the participants' own home. Information collected during this interview included health, cognitive function, and social circumstances. Participants were also asked to complete a self-completion questionnaire, which covered topics including health behaviours and personality. Self-completion questionnaire data were available on 705 participants.

For the present report, the sample was restricted to participants aged 50 to 81 years (n=909), as these participants would have been invited to take part in the Scottish Bowel Screening Programme.

Patient and Public Involvement

For peer review only - http://bmjopen.bmj.com/site/about/guidelines.xhtml

HAGIS participants were not involved in the development of any part of this study. The results of this study will be disseminated to participants and the public via the HAGIS website (www.hagis.scot).

Measures

CRC screening

As part of the face-to-face interview participants were asked "Have you ever completed a home testing kit for screening bowel cancer?". Individuals who answered don't know (n=6) or who refused to answer (n=3) were removed from this analysis.

Risk aversion

Risk aversion and time preference were assessed using Multiple Price Lists (MPL); a standard method for eliciting preferences in economics.[26] A MPL with three choices in the self-completion questionnaire was used to assess risk aversion (shown in Supplementary Materials). Participants were presented with a series of choices between a certain amount of monthly income of £1,500 or taking a gamble with a 50% chance of a higher monthly income and a 50% chance of a lower monthly income. The lower monthly income increased from £1,000 in question 1 to £1,300 in question 3. Less risk averse individuals will be more likely to choose the gamble. The measure of risk aversion was the point at which the participant switched from choosing the certain monthly income of £1,500, to choosing the gamble (score range 1 to 4). Higher scores reflect more risk aversion.

Time preference

For peer review only - http://bmjopen.bmj.com/site/about/guidelines.xhtml

Time preference was assessed using a MPL with seven choices administered in the self-completion questionnaire (shown in Supplementary Materials) that were based on a simplified version of the Kirby Delay-Discounting task.[27] Participants had to choose between receiving £1,500 now, or a higher amount of money in one month's time. The amount received in one month's time ranged from £1,506 to £1,596. If participants are very future-oriented, they will always pick the higher amount in one month's time. The measure of time preference used here was the point at which the participant switched from choosing the money now to choosing the money in one month's time (score range 1 to 8). A higher score reflects more present-orientation.

Health literacy

Health literacy was assessed during the face-to-face interview using a popular test of health literacy, the Newest Vital Sign (NVS).[28] The NVS assesses reading and numeracy skills required to understand health information.[28] Participants were presented with a nutrition label from a container of ice cream and were asked six questions about this information. For example, one question asked participants to work out how many calories they would eat if they ate the entire container of ice cream. The score is the sum of correct answers (maximum score=6).

Cognitive ability

Five tests of cognitive function that were assessed in the face-to-face interview were used here. These tests were designed to measure verbal declarative memory (immediate and delayed word recall), executive function (categorical fluency), processing speed (letter digit substitution), crystallised ability (vocabulary), and non-verbal reasoning (matrices). Scores on

For peer review only - http://bmjopen.bmj.com/site/about/guidelines.xhtml

the five cognitive tests were entered into a principal components analysis and the score on the first unrotated component was saved and used as a measure of general cognitive ability (mean=0.00, SD=1.00). Descriptions of each cognitive test and more detail on the creation of the general cognitive ability measure are reported in the Supplementary Methods.

Personality traits

Personality was assessed as part of the self-completion questionnaire using the 50-item IPIP questionnaire which measures the Big-Five personality traits of extraversion, agreeableness, conscientiousness, emotional stability (the opposite of neuroticism), and intellect/imagination.[29] This questionnaire consists of ten items for each personality factor. Participants are presented with a statement (e.g., "I make people feel at ease") and were asked to rate how accurately each statement described them on a 5-point Likert scale (very inaccurate to very accurate). The score for each factor is the sum of these ten items.

Covariates

Age in days, sex, living arrangement, social deprivation and educational qualifications were assessed as part of the face-to-face interview. Participants who reported they were married or living with their partner as if married were categorised as cohabiting. Individuals who reported they were single, separated, divorced, or widowed were categorised as living alone. Deprivation was measured using Scottish Index of Multiple Deprivation (SIMD) quartiles,[30] which ranks participant's level of deprivation based on the amount of employment, income, health, education, housing, access and crime in the area where they live. Education was assessed by asking participants "What is the highest level of education you have completed?". Qualifications were coded as 1=primary or less, 2=O level/O grade or

For peer review only

For peer review only - http://bmjopen.bmj.com/site/about/guidelines.xhtml

equivalent, 3=Highers/sixth year studies/HNC/HND or equivalent, 4=first degree, 5=postgraduate/higher degree.

Statistical analysis

To test whether individuals who have participated in CRC screening differ from non-participants on demographic, socioeconomic and psychological variables, t-tests were performed for normally distributed variables, Mann-Whitney U tests were used for non-normally distributed variables, and χ^2 tests were used for categorical variables. Spearman-rank correlations were calculated to examine the associations between predictor variables. Logistic regression was used to examine the association of psychological factors with CRC screening uptake. For each model, participants with missing data were removed. The outcome variable was coded 1 for reporting participating in CRC screening, and 0 if not. Age, age-squared, sex, living arrangement, and an interaction between sex and living arrangement were entered into all models. To test the association of each psychological variable with CRC screening, models were run in which each of the psychological variables (risk aversion, time preference, health literacy, cognitive ability, and personality traits), qualifications, and deprivation were entered individually. To determine whether any associations between risk aversion, time preference, health literacy, cognitive ability and personality with CRC screening were independent of the other psychological traits, a multivariate logistic regression model was run in which these psychological variables were entered into the same model. A fully-adjusted model was then run which additionally adjusted for qualifications and deprivation. Finally, a backward elimination logistic regression was run to determine the best independent correlates of CRC screening participation. The p-values reported have been

For peer review only - http://bmjopen.bmj.com/site/about/guidelines.xhtml

false discovery rate (FDR) corrected to control for multiple comparisons. The FDR method controls for the number of false positive results in those tests that reach significance.

RESULTS

After removal of one participant who did not have data on whether they had completed the CRC screening test, a total of 908 (mean age=65.85, SD=8.23) participants were aged between 50 and 81 years and answered yes (n=683) or no (n=225) to whether they have ever completed a home bowel screening test. Participant characteristics according to screening status are shown in Table 1. Individuals who participated in CRC screening were older, more likely to be cohabiting, have higher qualifications and be less likely to live in deprived areas, compared to non-participants. Screeners were less risk averse, more future-oriented, and scored higher on the NVS, cognitive ability, emotional stability and intellect.

The rank-order correlations and partial correlations, controlling for age and sex, between all covariates are reported in Supplementary Table 1. Many of the psychological variables have moderate correlations with each other. Notable among them, higher cognitive ability was moderately correlated with higher scores on the NVS (rho=0.45, p<0.001). Risk aversion and present-orientation were correlated positively with each other (rho=0.14, p=0.002), and negatively with cognitive ability (risk aversion rho=-0.17, p<0.001; present-orientation rho=-0.11, p<0.001) and the NVS (risk aversion rho=-0.17, p<0.001; present-orientation rho=-0.11,p=0.01). Cognitive ability was positively correlated with all five personality traits (rho=0.17 to 0.39, p<0.001).

Table 2 shows the ORs (95% CI) for participating in CRC screening, for each psychological variable entered separately, adjusting for age, age-squared, sex, living arrangement and sex*living arrangement. More risk averse (OR=0.67, 0.51 to 0.85) and more present-oriented

For peer review only - http://bmjopen.bmj.com/site/about/guidelines.xhtml

For peer review only

(OR=0.86, 0.80 to 0.94) individuals were less likely to participate in CRC screening. Individuals with higher NVS (OR=1.20, 1.09 to 1.31), cognitive ability (OR=1.51, 1.25 to 1.81) and intellect (OR=1.05, 1.01 to 1.09) scores were more likely to screen. Higher qualifications (OR=1.27, 1.11 to 1.47) and less deprivation (OR=1.32, 1.15 to 1.53) were associated with increased odds of screening.

Next, all of the psychological variables were entered into the model simultaneously (Table 3, Model 1). The association between risk aversion and CRC screening became stronger (OR=0.52, 0.36 to 0.76). Adjusting for all other psychological variables, the associations between time preference, the NVS, cognitive ability and intellect with CRC screening participation were reduced in size and non-significant. The association between risk aversion and CRC screening remained almost unchanged when additionally adjusting for qualifications and deprivation (Table 3, Model 2). In this fully-adjusted model, older age was associated with increased odds of screening. The significant age-squared term suggests that this association became slightly weaker with increased age. Participants living alone, compared to those cohabiting, were less likely to screen; however, this association varied by sex. The odds of participating in CRC screening were lower for male participants living alone than female participants living alone. Participants living in areas of less deprivation were more likely to screen.

A backwards elimination logistic regression run from the fully-adjusted model retained age, age-squared, living arrangement, risk aversion, cognitive ability and SIMD (Table 3; Model 3). Individuals who were more risk averse were less likely to participate in CRC screening (OR=0.55, 0.39 to 0.78), whereas higher cognitive ability (OR=1.42, 1.05 to 1.93) was associated with increased odds of screening. Older age was associated with increased odds of screening. This age-association became slightly weaker with increasing age. Living in areas with greater deprivation was associated with reduced odds of screening.

For peer review only - http://bmjopen.bmj.com/site/about/guidelines.xhtml

Only 384 participants had complete data. Demographic characteristics were compared between participants with and without complete data (Supplementary Table 2). Those with complete data were more likely to be cohabiting, had higher qualifications, and were less deprived than non-completers. The models reported in Table 2 were re-run using participants with complete data. Using only participants with complete data, the associations between higher NVS and intellect with CRC screening were similar in size but were non-significant (Supplementary Table 3). The association between risk aversion and screening was stronger (OR=0.51, 0.35 to 0.72) using this smaller sample. The increase in the strength of the association between risk aversion and CRC screening seen when risk aversion was entered simultaneously with other psychological variables appears to be due to the reduction in sample size.

DISCUSSION

Using a sample of middle-aged and older adults living in Scotland, this study found that, when examining each psychological variable separately, participants who were more risk averse and more present-oriented were less likely to have participated in CRC screening, whereas individuals who had higher health literacy, cognitive ability, and intellect were more likely to have participated in CRC screening. When entering these psychological variables simultaneously in the same model, risk averse individuals were consistently less likely to have completed a home CRC screening test, even when additionally adjusting for socioeconomic indicators. Although cognitive ability was not associated with CRC screening in the fully-adjusted model, higher cognitive ability was associated with increased odds of participating in CRC screening in the backwards elimination logistic regression, thus there is some evidence that cognitive ability may be associated with CRC screening. Time

For peer review only - http://bmjopen.bmj.com/site/about/guidelines.xhtml

preference, health literacy, and intellect were no longer associated with CRC screening when all psychological traits were entered simultaneously, suggesting that these psychological traits do not have associations with CRC screening that are independent of the other psychological traits measured.

Finding that risk averse individuals are less likely to take part in CRC screening is in line with another study that reported that risk averse women were less likely to regularly undergo breast cancer screening.[18] The outcomes of treating cancer are uncertain. Risk averse individuals may be more sensitive to this uncertainty compared to a risk seeking individuals.[18] Emphasising how CRC screening can reduce risk of future ill-health, e.g., by increasing survival rates and reducing the need for more invasive and uncertain cancer treatment, may encourage risk averse individuals to undergo the test.[18]

The current study found some support that higher cognitive ability was associated with increased rates of screening. Completing a home CRC screening test may be a cognitively demanding task, and therefore individuals with lower cognitive ability might struggle to successfully complete this task. Using a sample of English adults, Gale et al.[10] also found some support that higher cognitive ability predicted participation in a home CRC screening test; however, the association between cognitive ability and CRC screening was attenuated and non-significant when concurrently measuring conscientiousness, health literacy, and household wealth. The association between higher cognitive ability and increased CRC screening may not be independent of other psychological and socioeconomic variables.

This study has a number of advantages, including that the HAGIS pilot study was designed to be representative of Scottish adults aged over 50 years and that the HAGIS study measured a wide range of psychological characteristics. One limitation is that one-third of participants did not return the self-completion questionnaire, therefore the fully-adjusted models reported

For peer review only - http://bmjopen.bmj.com/site/about/guidelines.xhtml

here are likely not representative of the Scottish population. Another limitation is that this study examined whether participants have ever screened, and not whether they regularly take part in CRC screening, which is important for detecting cancer early.[3] Future studies should investigate the psychological correlates of regularly participating in CRC screening.

In this sample of Scottish middle-aged and older adults, individuals who were risk averse were less likely to report that they had ever completed a home CRC screening test, even when adjusting for a range of other psychological and socioeconomic measures. There was also some indication that higher cognitive ability may be associated with increased likelihood of having participated in CRC screening. Educational materials that emphasise how CRC screening can reduce the risk of future ill-health may encourage risk averse individuals to participate in CRC screening.

For peer review only

For peer review only - http://bmjopen.bmj.com/site/about/guidelines.xhtml

Table 1 Participant characteristics according to colorectal cancer screening participation status

	N	Yes (n=683)	No (n=225)	Effect size	p value for a difference
Age (years), mean (SD)	908	66.51 (7.95)	63.87 (8.76)	*r*=0.136	<0.001
Sex, n (%)	908			*Φ*=0.011	0.741
Female		382 (55.9%)	123 (54.7%)		
Male		301 (44.1%)	102 (45.3%)		
Living Arrangement, n (%)	908			*Φ*=0.122	<0.001
Cohabiting		474 (69.4%)	126 (56.0%)		
Living alone		209 (30.6%)	99 (44.0%)		
Qualifications, n (%)	902			Φ_c=0.123	0.009
Primary or less		117 (17.3%)	50 (22.3%)		
O level/O grade		194 (28.6%)	81 (36.2%)		
Highers/Sixth year studies/HNC/HND		208 (30.7%)	53 (23.6%)		
First degree		90 (13.3%)	29 (12.9%)		
Postgraduate/higher degree		69 (10.2%)	11 (4.9%)		
SIMD, n (%)	908			*Φ*=0.163	<0.001
1 (Most deprived)		139 (20.4%)	73 (32.4%)		
2		136 (19.9%)	59 (26.2%)		
3		213 (31.2%)	50 (22.2%)		
4 (Least deprived)		195 (28.6%)	43 (19.1%)		
Risk aversion, n (%)	581				
1 (Risk seeking)		47 (10.3%)	8 (6.5%)		
2		46 (10.1%)	6 (4.8%)		
3		55 (12.0%)	12 (9.7%)		
4 (Risk averse)		309 (67.6%)	98 (79.0%)		
Risk aversion, mean (SD)		3.37 (1.03)	3.61 (0.85)	*r*=0.104	0.012
Time preference, n (%)	504				
1 (Future-oriented)		159 (39.7%)	24 (23.3%)		
2		27 (6.7%)	4 (3.9%)		
3		20 (5.0%)	2 (1.9%)		
4		34 (8.5%)	8 (7.8%)		
5		20 (5.0%)	3 (2.9%)		
6		17 (4.2%)	15 (14.6%)		
7		44 (11.0%)	21 (20.4%)		
8 (Present-oriented)		80 (20.0%)	26 (25.2%)		
Time preference, mean (SD)		3.89 (2.89)	5.15 (2.74)	*r*=0.159	<0.001
Newest Vital Sign, mean (SD)	867	4.51 (1.68)	4.07 (1.86)	*r*=0.103	0.002
Cognitive ability, mean (SD)	790	0.06 (0.98)	-0.21 (1.06)	*g*=0.273	0.001
Extraversion, mean (SD)	575	30.64 (7.17)	30.71 (7.17)	*g*=0.009	0.929
Agreeableness, mean (SD)	566	40.18 (5.95)	39.88 (6.40)	*r*=0.010	0.804
Conscientiousness, mean (SD)	547	37.77 (6.04)	36.59 (6.63)	*g*=0.191	0.065
Emotional stability, mean (SD)	575	33.68 (7.22)	31.99 (7.97)	*g*=0.229	0.035
Intellect, mean (SD)	565	33.53 (5.61)	32.14 (5.91)	*r*=0.112	0.008

SIMD, Scottish Index of Multiple Deprivation; *r*, effect size correlation coefficient; *Φ*, Phi; Φ_c, Cramer's *V*; *g*, Hedge's *g*.

For peer review only - http://bmjopen.bmj.com/site/about/guidelines.xhtml

Table 2 Odd ratios (95% CI) for participating in colorectal cancer screening

	n	OR (95% CI)
Risk aversion	581	0.66 (0.51 to 0.85)**
Time preference	504	0.86 (0.80 to 0.94)**
Newest Vital Sign	867	1.20 (1.09 to 1.31)**
Cognitive ability	790	1.51 (1.25 to 1.81)***
Extraversion	575	0.99 (0.96 to 1.02)
Agreeableness	566	1.01 (0.97 to 1.04)
Conscientiousness	547	1.03 (1.00 to 1.07)
Emotional stability	575	1.03 (1.00 to 1.06)
Intellect	565	1.05 (1.01 to 1.09)*
Qualifications	902	1.27 (1.11 to 1.47)**
SIMD	908	1.32 (1.15 to 1.53)**

ORs (95% CIs) reported are for each psychological factor entered individually, adjusting for age, age-squared, sex, living arrangement and sex*living arrangement.
SIMD, Scottish Index of Multiple Deprivation.
*p<0.05, **p<0.01, ***p<0.001. P-values are false discovery rate corrected.

For peer review only

Table 3 Odd ratios (95% CIs) for participating in colorectal cancer screening

	Model 1: All psychological variables entered simultaneously (n=388)	Model 2: Additionally adjusting for indicators of SES (n=384)	Model 3: Backward elimination model (n=384)
Age	1.08 (1.04 to 1.12)***	1.08 (1.04 to 1.12)**	1.09 (1.05 to 1.12)***
Age^2	0.99 (0.99 to 0.99)**	0.99 (0.99 to 0.99)**	0.995 (0.991 to 0.999)*
Sex			
Female	Reference	Reference	-
Male	0.60 (0.32 to 1.11)	0.61 (0.33 to 1.15)	-
Living arrangement			
Cohabiting	Reference	Reference	Reference
Living alone	0.40 (0.22 to 0.73)**	0.43 (0.23 to 0.78)*	1.86 (1.06 to 3.28)
Sex*Living arrangement	0.24 (0.07 to 0.81)*	0.24 (0.07 to 0.81)*	-
Risk aversion	0.52 (0.36 to 0.76)**	0.53 (0.36 to 0.77)**	0.55 (0.39 to 0.78)**
Time preference	0.94 (0.85 to 1.04)	0.98 (0.88 to 1.08)	-
Newest Vital Sign	1.02 (0.84 to 1.24)	1.02 (0.84 to 1.25)	-
Cognitive ability	1.38 (0.97 to 1.97)	1.26 (0.86 to 1.85)	1.42 (1.05 to 1.93)*
Extraversion	0.97 (0.92 to 1.01)	0.97 (0.92 to 1.01)	-
Agreeableness	0.95 (0.90 to 1.02)	0.96 (0.90 to 1.03)	-
Conscientiousness	1.04 (0.99 to 1.10)	1.04 (0.98 to 1.10)	-
Emotional stability	1.03 (0.98 to 1.07)	1.02 (0.98 to 1.07)	-
Intellect	1.02 (0.96 to 1.08)	1.01 (0.95 to 1.08)	-
Qualifications	-	1.06 (0.78 to 1.45)	-
SIMD	-	1.40 (1.05 to 1.87)*	1.47 (1.05 to 1.92)*

Model 3 reports the results of a backward elimination model using all variables entered in the fully-adjusted model (Model 2).
SES, socioeconomic status; Age^2, Age-squared; SIMD, Scottish Index of Multiple Deprivation.
*p<0.05, **p<0.01, ***p<0.001. P-values are false discovery rate corrected.

For peer review only

Acknowledgements We thank the HAGIS participants.

Completing Interests None.

Funding This work was supported by the University of Edinburgh Centre for Cognitive Ageing and Cognitive Epidemiology, part of the cross council Lifelong Health and Wellbeing Initiative, funded by the Biotechnology and Biological Sciences Research Council (BBSRC) and Medical Research Council (MRC) (grant no MR/K026992/1). The HAGIS study (data resource) was part-funded by the National Institute of Aging (RAG044535A) in the USA and was also part-funded by the Nuffield Foundation (OPD/42197), but the views expressed are those of the authors and not necessarily those of the Foundation. MvdP is supported by the Chief Scientist Office (CSO) of the Scottish Government Health and Social Care Directorates (SGHSC). The views expressed here are those of the Unit and not necessarily those of the CSO.

Author Contributions Chloe Fawns-Ritchie contributed to the conception and design of the project, analysed the data, interpreted the data, and drafted the initial manuscript. Christopher B. Miller contributed to the conception and design of the project, analysed the data, interpreted the data, and drafted the initial manuscript. Marjon van der Pol contributed to the statistical analysis of the data, interpreted the data, and critically revised the manuscript. Elaine Douglas interpreted the data, and critically revised the manuscript. David Bell Douglas interpreted the data, and critically revised the manuscript. Ronan E. O'Carroll contributed to the conception and design of the project, interpreted the data, and critically revised the manuscript. Ian J. Deary contributed to the conception and design of the project, interpreted the data, and critically revised the manuscript.

Data Availability Statement The anonymised HAGIS pilot data will be made available via the Gateway to Global Aging Data: https://g2aging.org/

For peer review only - http://bmjopen.bmj.com/site/about/guidelines.xhtml

REFERENCES

1 NHS Information Services Division. Cancer Incidence in Scotland (2016). Scotland: NHS Nationl Services Scotland 2018.

2 Scholefield J, Moss S, Mangham C*, et al.* Nottingham trial of faecal occult blood testing for colorectal cancer: a 20-year follow-up. *Gut* 2012;**61**:1036-40. doi: 10.1136/gutjnl-2011-300774

3 NHS Information Services Division. Scottish Bowel Screening Programme Statistics. Scotland: NHS National Services Scotland 2017.

4 Mansouri D, McMillan DC, Grant Y*, et al.* The impact of age, sex and socioeconomic deprivation on outcomes in a colorectal cancer screening programme. *PLoS One* 2013;**8**:e66063. doi: 10.1371/journal.pone.0066063

5 Quyn AJ, Fraser CG, Stanners G*, et al.* Uptake trends in the Scottish Bowel Screening Programme and the influences of age, sex, and deprivation. *J Med Screen* 2017:969141317694065. doi: 10.1177/0969141317694065

6 Heflin MT, Oddone EZ, Pieper CF*, et al.* The effect of comorbid illness on receipt of cancer screening by older people. *J Am Geriatr Soc* 2002;**50**:1651-8. doi: 10.1046/j.1532-5415.2002.50456.x

7 Leung DY, Leung AY, Chi I. Breast and colorectal cancer screening and associated correlates among Chinese older women. *Asian Pac J Cancer Prev* 2012;**13**:283-7. doi: 10.7314/apjcp.2012.13.1.283

8 Sen CK, Kumkale GT. Who does not get screened? A simple model of the complex relationships in mammogram non-attendance. *J Health Psychol* 2016;**21**:2838-50. doi: 10.1177/1359105315587138

For peer review only - http://bmjopen.bmj.com/site/about/guidelines.xhtml

9 Arai S, Nakaya N, Kakizaki M*, et al.* Personality and gastric cancer screening attendance: a cross-sectional analysis from the Miyagi Cohort Study. *J Epidemiol* 2009;**19**:34-40. doi: 10.2188/jea.je20080024

10 Gale CR, Deary IJ, Wardle J*, et al.* Cognitive ability and personality as predictors of participation in a national colorectal cancer screening programme: the English Longitudinal Study of Ageing. *J Epidemiol Community Health* 2015;**69**:530-5. doi: 10.1136/jech-2014-204888

11 Baker DW. The meaning and the measure of health literacy. *J Gen Intern Med* 2006;**21**:878-83. doi: 10.1111/j.1525-1497.2006.00540.x

12 Kobayashi LC, Wardle J, von Wagner C. Limited health literacy is a barrier to colorectal cancer screening in England: evidence from the English Longitudinal Study of Ageing. *Prev Med* 2014;**61**:100-5. doi: 10.1016/j.ypmed.2013.11.012

13 Solmi F, Von Wagner C, Kobayashi LC*, et al.* Decomposing socio-economic inequality in colorectal cancer screening uptake in England. *Soc Sci Med* 2015;**134**:76-86. doi: 10.1016/j.socscimed.2015.04.010

14 Peterson NB, Dwyer KA, Mulvaney SA*, et al.* The influence of health literacy on colorectal cancer screening knowledge, beliefs and behavior. *J Natl Med Assoc* 2007;**99**:1105-12.

15 Oldach BR, Katz ML. Health literacy and cancer screening: a systematic review. *Patient education and counseling* 2014;**94**:149-57. doi: 10.1016/j.pec.2013.10.001

16 Bradford WD. The association between individual time preferences and health maintenance habits. *Med Decis Making* 2010;**30**:99-112. doi: 10.1177/0272989x09342276

For peer review only

For peer review only - http://bmjopen.bmj.com/site/about/guidelines.xhtml

17 Bradford WD, Zoller J, Silvestri GA. Estimating the effect of individual time preferences on the use of disease screening. *Southern Economic Journal* 2010;**76**:1005-31. doi: 10.4284/sej.2010.76.4.1005

18 Goldzahl L. Contributions of risk preference, time orientation and perceptions to breast cancer screening regularity. *Soc Sci Med* 2017;**185**:147-57. doi: 10.1016/j.socscimed.2017.04.037

19 Picone G, Sloan F, Taylor D. Effects of risk and time preference and expected longevity on demand for medical tests. *J Risk Uncertain* 2004;**28**:39-53. doi: 10.1023/b:risk.0000009435.11390.23

20 Whitaker KL, Good A, Miles A*, et al.* Socioeconomic inequalities in colorectal cancer screening uptake: does time perspective play a role? *Health Psychol* 2011;**30**:702-9. doi: 10.1037/a0023941

21 Reeve CL, Basalik D. Is health literacy an example of construct proliferation? A conceptual and empirical evaluation of its redundancy with general cognitive ability. *Intelligence* 2014;**44**:93-102. doi: 10.1016/j.intell.2014.03.004

22 Shamosh NA, Gray JR. Delay discounting and intelligence: A meta-analysis. *Intelligence* 2008;**36**:289-305. doi: 10.1016/j.intell.2007.09.004

23 Boyle PA, Yu L, Wilson RS*, et al.* Cognitive decline impairs financial and health literacy among community-based older persons without dementia. *Psychology and aging* 2013;**28**:614-24. doi: 10.1037/a0033103

24 Becker A, Deckers T, Dohmen T*, et al.* The relationship between economic preferences and psychological personality measures. *Annu Rev Econom* 2012;**4**:453-78. doi: 10.2139/ssrn.2199369

For peer review only - http://bmjopen.bmj.com/site/about/guidelines.xhtml

25 Douglas E, Rutherford A, Bell D. Pilot study protocol to inform a future longitudinal study of ageing using linked administrative data: Healthy AGeing in Scotland (HAGIS). *BMJ Open* 2018;**8**:e018802. doi: 10.1136/bmjopen-2017-018802

26 Andersen S, Harrison GW, Lau MI*, et al.* Elicitation using multiple price list formats. *Exp Econ* 2006;**9**:383-405. doi: 10.1007/s10683-008-9204-6

27 Kirby KN, Petry NM, Bickel WK. Heroin addicts have higher discount rates for delayed rewards than non-drug-using controls. *Journal of Experimental psychology: general* 1999;**128**:78. doi: 10.1111/j.1360-0443.2003.00669.x

28 Weiss BD, Mays MZ, Martz W*, et al.* Quick assessment of literacy in primary care: the newest vital sign. *Ann Fam Med* 2005;**3**:514-22. doi: 10.1370/afm.405

29 Goldberg LR. The development of markers for the Big-Five factor structure. *Psychol Assess* 1992;**4**:26. doi: 10.1037//1040-3590.4.1.26

30 Scottish Government. Scottish Index of Multiple Deprivation 2016. Scotland 2016.

For peer review only

For peer review only - http://bmjopen.bmj.com/site/about/guidelines.xhtml

Supplementary Materials for: Fawns-Ritchie et al. Psychological correlates of free colorectal cancer screening uptake in a Scottish sample

HAGIS risk aversion questions

1. Income A, which will with certainty give you a £1,500 per month for the rest of your life OR Income B which will give you a 50-50 chance of £3,000 and a 50-50 chance of £1,000 per month for the rest of your life.

Would you choose Income A or Income B?
(a) Income A
(b) Income B

2. Income A, which will with certainty give you a £1,500 per month for the rest of your life OR Income B, which will give you a 50-50 chance of £3,000 and a 50-50 chance of £1,200 per month for the rest of your life.

Would you choose Income A or Income B?
(a) Income A
(b) Income B

3. Income A, which will with certainty give you a £1,500 per month for the rest of your life OR Income B, which will give you a 50-50 chance of £3,000 and a 50-50 chance of £1,300 per month for the rest of your life.

Would you choose Income A or Income B?
(a) Income A
(b) Income B

For peer review only

For peer review only - http://bmjopen.bmj.com/site/about/guidelines.xhtml

HAGIS time orientation questions

1. Would you rather have £1,500 now or £1,506 a month from now?
   (a) £1,500 now
   (b) £1,506 a month from now

2. Would you rather have £1,500 now or £1,512 a month from now?
   (a) £1,500 now
   (b) £1,512 a month from now

3. Would you rather have £1,500 now or £1,518 a month from now?
   (a) £1,500 now
   (b) £1,518 a month from now

4. Would you rather have £1,500 now or £1,524 a month from now?
   (a) £1,500 now
   (b) £1,524 a month from now

5. Would you rather have £1,500 now or £1,536 a month from now?
   (a) £1,500 now
   (b) £1,536 a month from now

6. Would you rather have £1,500 now or £1,548 a month from now?
   (a) £1,500 now
   (b) £1,548 a month from now

7. Would you rather have £1,500 now or £1,596 a month from now?
   (a) £1,500 now
   (b) £1,596 a month from now

For peer review only

SUPPLEMENTARY METHODS

Cognitive ability

Five tests of cognitive function that were assessed during the face-to-face interview were used here. Verbal declarative memory was assessed using the word list learning test in which participants were presented with a list of 10 words and had to recall these words immediately, and again after an approximately 20 minute delay. The scores for word list learning immediate and delayed tests correlated at $r=0.66$ ($p<0.001$) therefore, the score used here is the total number of words recalled across the two tests (range 0-20). Categorical (animal) fluency, in which participants are asked to name as many animals as possible, was used to measure executive function. The score is the number of animals named in 60 seconds. The word list learning and animal fluency tests have been administered in other longitudinal studies of ageing including the Health and Retirement Study[1] and the English Longitudinal Study of Ageing.[2] Processing speed was measured using the letter digit substitution test.[3] Here, participants were presented with a piece of paper with a key pairing letters with numbers. Below the key were rows of letters with empty boxes underneath each letter. Participants were to use this key to write in the number that is paired with each letter. The score is the number of correct letter-digit matches made in 60 seconds. Two tests from the COGNITO neuropsychological test battery were administered to assess vocabulary and non-verbal fluid reasoning.[4] A shortened version of the COGNITO vocabulary test[4] was used. Participants were presented with a target word and were instructed to select which of six alternatives means the same as the target word. There were 20 items and the score is the number of correctly answered questions. COGNITO matrices[4] was used to assess non-verbal reasoning. Participants were presented with 15 matrix designs with a piece missing

For peer review only - http://bmjopen.bmj.com/site/about/guidelines.xhtml

and they were required to select the missing piece from a list of alternatives. The score is the number of correctly answered questions in 4 minutes.

To create a measure of general cognitive ability, scores on the five cognitive tests were entered into a principal components analysis. The eigenvalues and scree plot (Supplementary Figure 1) indicated that one component should be extracted. The first unrotated principal component accounted for 44% of the total variance across the five tests. The loadings for the five tests were: total word recall=0.60, animal fluency=0.73, letter digit substitution=0.70, vocabulary=0.58, matrices=0.68. The score from the first unrotated principal component was used here as a measure of general cognitive ability (mean=0.00, SD=1.00).

For peer review only

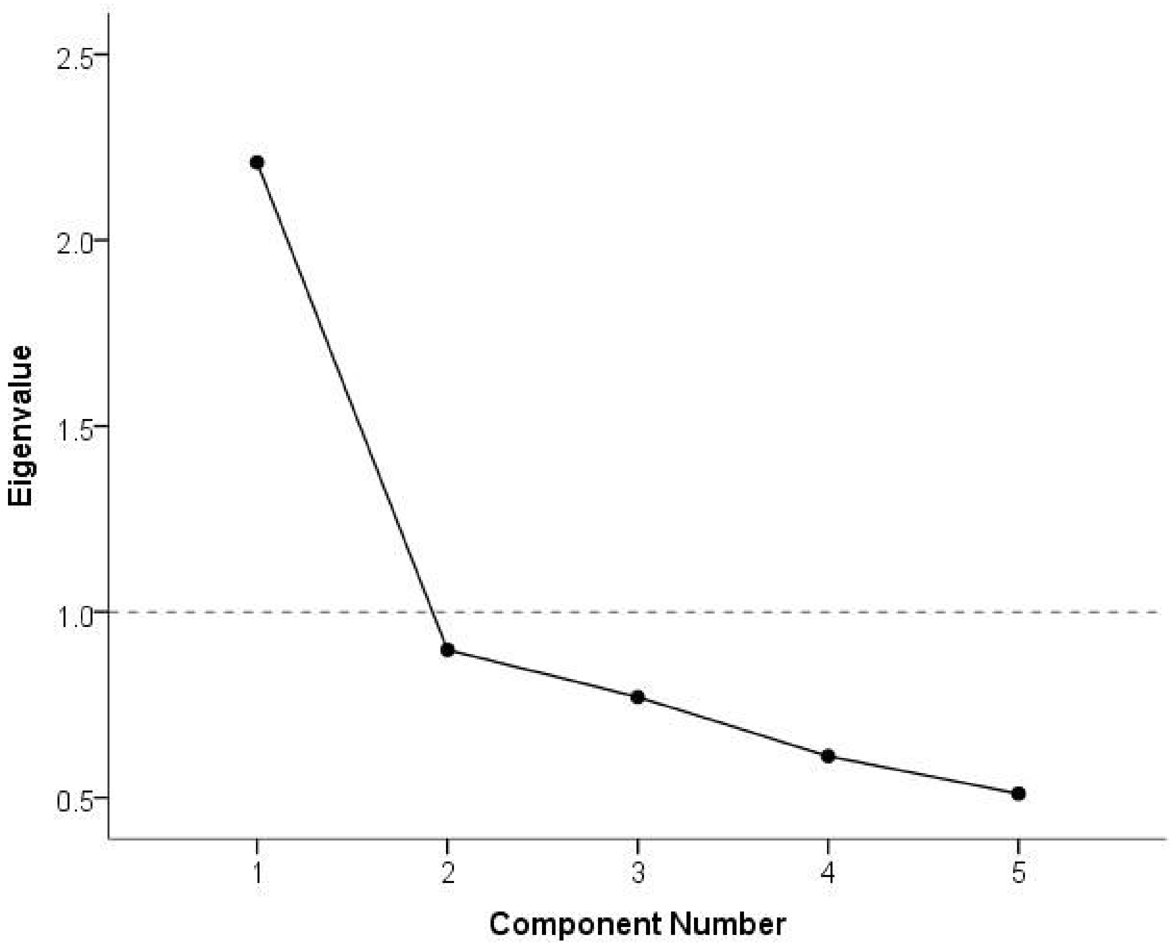

Supplementary Figure 1 Scree plot of eigenvalues from the 5 HAGIS cognitive tests

For peer review only - http://bmjopen.bmj.com/site/about/guidelines.xhtml

Supplementary Table 1 Rank order correlations (below the diagonal) and partial correlations controlling for age and sex (above the diagonal) between covariates

	1	2	3	4	5	6	7	8	9	10	11	12	13	14
1. Age	-	-	-	-	-	-	-	-	-	-	-	-	-	-
2. Sex	0.04	-	-	-	-	-	-	-	-	-	-	-	-	-
3. Cohabiting	-0.04	0.18***	-	0.12***	0.17***	-0.08	-0.09*	0.17***	0.15***	0.12**	0.07	0.08	0.04	0.13**
4. Qualifications	-0.16***	0.07*	0.14***	-	0.39***	-0.22***	-0.27***	0.22***	0.48***	-0.03	-0.05	0.01	0.13*	0.36***
5. SIMD	0.03	0.03	0.17***	0.35***	-	-0.18***	-0.29***	0.10*	0.22***	-0.04	-0.04	-0.02	0.04	0.14**
6. Risk aversion	0.18***	-0.08	-0.10*	-0.21***	-0.15***	-	0.15**	-0.18***	-0.12*	-0.01	0.09	0.05	0.03	-0.15**
7. Time preference	-0.01	-0.11*	-0.11*	-0.24***	-0.28***	0.14**	-	-0.11*	-0.16**	0.12*	0.09	0.06	0.03	-0.13**
8. Newest Vital Sign	-0.20***	-0.04	0.17***	0.22***	0.05	-0.17***	-0.11*	-	0.44***	0.06	-0.01	0.06	0.10	0.23***
9. Cognitive ability	-0.28***	-0.04	0.15***	0.48***	0.26***	-0.17***	-0.11***	0.45***	-	0.14**	0.13*	0.20***	0.23***	0.37***
10. Extraversion	-0.03	-0.04	0.11**	0.00	0.01	-0.06	0.07	0.11**	0.17***	-	0.37***	0.22***	0.27***	0.27***
11. Agreeableness	-0.08	-0.29***	0.01	0.03	0.00	0.07	0.10*	0.08	0.22***	0.37***	-	0.45***	0.30***	0.27***
12. Conscientiousness	-0.05	-0.09*	0.06	0.09*	-0.02	0.01	0.04	0.13**	0.23***	0.20***	0.45***	-	0.33***	0.21***
13. Emotional stability	0.06	0.05	0.05	0.13**	0.04	0.03	0.03	0.08	0.18***	0.27***	0.25***	0.30***	-	0.09
14. Intellect	-0.08	0.06	0.14**	0.36***	0.13**	-0.18***	-0.12**	0.22	0.39***	0.28***	0.27***	0.30***	0.08	-

SIMD, Scottish Index of Multiple Deprivation.
Sex is coded 0 for female, 1 for male. Cohabiting is coded 0 for living alone, 1 for cohabiting.
*p<0.05, **p<0.01, ***p<0.001.

For peer review only

Supplementary Table 2 Participant characteristics according to whether they have complete data on all variables of interest (completers) or whether they had missing data (non-completers)

	N	Completers (n=384)	Non-completers (n=524)	p value for a difference
Age (years), mean (SD)	908	65.33 (8.10)	66.24 (8.31)	0.101
Sex, n (%)	908			0.953
Female		214 (55.7%)	291 (55.5%)	
Male		170 (44.3%)	233 (44.5%)	
Living Arrangement, n (%)	908			0.003
Cohabiting		275 (71.6%)	325 (62.0%)	
Living alone		109 (28.4%)	199 (38.0%)	
Qualifications, n (%)	902			<0.001
Primary or less		43 (11.2%)	124 (23.9%)	
O level/O grade		113 (29.4%)	162 (31.3%)	
Highers/Sixth year studies/HNC/HND		113 (29.4%)	148 (28.6%)	
First degree		62 (16.1%)	57 (11.0%)	
Postgraduate/higher degree		53 (13.8%)	27 (5.2%)	
SIMD, n (%)	908			0.007
1 (Most deprived)		68 (17.7%)	144 (27.5%)	
2		86 (22.4%)	109 (20.8%)	
3		121 (31.5%)	142 (27.1%)	
4 (Least deprived)		109 (28.4%)	129 (24.6%)	

SIMD, Scottish Index of Multiple Deprivation.

For peer review only

Supplementary Table 3 Odd ratios (95% CIs) for predictors of participation in colorectal cancer screening using participants with complete data (n=384)

	OR (95% CI)
Risk aversion	0.51 (0.35 to 0.72)***
Time preference	0.90 (0.82 to 0.98)*
Newest Vital Sign	1.17 (0.99 to 1.37)
Cognitive ability	1.56 (1.17 to 2.08)**
Extraversion	0.98 (0.94 to 1.01)
Agreeableness	0.97 (0.92 to 1.02)
Conscientiousness	1.02 (0.98 to 1.06)
Emotional stability	1.02 (0.98 to 1.06)
Intellect	1.05 (1.00 to 1.10)
Qualifications	1.44 (1.14 to 1.83)**
SIMD	1.65 (1.28 to 2.13)***

ORs (95% CIs) reported are for each psychological factor entered individually, adjusting for age, age-squared, sex, living arrangement, and sex*living arrangement.
SIMD, Scottish Index of Multiple Deprivation.
*p<0.05, **p<0.01, ***p<0.001.

For peer review only

SUPPLEMENTARY REFERENCES

1 Sonnega A, Faul JD, Ofstedal MB*, et al.* Cohort Profile: the Health and Retirement Study (HRS). *International journal of epidemiology* 2014;**43**:576-85. doi: 10.1093/ije/dyu067

2 Steptoe A, Breeze E, Banks J*, et al.* Cohort profile: the English longitudinal study of ageing. *International journal of epidemiology* 2013;**42**:1640-8. doi: 10.1093/ije/dys168

3 van der Elst W, van Boxtel MP, van Breukelen GJ*, et al.* The Letter Digit Substitution Test: normative data for 1,858 healthy participants aged 24–81 from the Maastricht Aging Study (MAAS): influence of age, education, and sex. *J Clin Exp Neuropsychol* 2006;**28**:998-1009. doi: 10.1080/13803390591004428

4 Ritchie K, de Roquefeuil G, Ritchie C*, et al.* COGNITO: computerized assessment of information processing. *J Psychol Psychother* 2014;**4**:136. doi: 10.4172/2161-0487.1000136

For peer review only

For peer review only - http://bmjopen.bmj.com/site/about/guidelines.xhtml

STROBE Statement—Checklist of items that should be included in reports of ***cross-sectional studies***

	Item No	Recommendation	Page No
**Title and abstract**	1	(*a*) Indicate the study's design with a commonly used term in the title or the abstract	1-2
		(*b*) Provide in the abstract an informative and balanced summary of what was done and what was found	2
**Introduction**			
Background/rationale	2	Explain the scientific background and rationale for the investigation being reported	5-6
Objectives	3	State specific objectives, including any prespecified hypotheses	6
**Methods**			
Study design	4	Present key elements of study design early in the paper	6
Setting	5	Describe the setting, locations, and relevant dates, including periods of recruitment, exposure, follow-up, and data collection	7
Participants	6	(*a*) Give the eligibility criteria, and the sources and methods of selection of participants	7
Variables	7	Clearly define all outcomes, exposures, predictors, potential confounders, and effect modifiers. Give diagnostic criteria, if applicable	7-10
Data sources/ measurement	8*	For each variable of interest, give sources of data and details of methods of assessment (measurement). Describe comparability of assessment methods if there is more than one group	7-10
Bias	9	Describe any efforts to address potential sources of bias	13-14
Study size	10	Explain how the study size was arrived at	7, 11
Quantitative variables	11	Explain how quantitative variables were handled in the analyses. If applicable, describe which groupings were chosen and why	10-11
Statistical methods	12	(*a*) Describe all statistical methods, including those used to control for confounding	10-11
		(*b*) Describe any methods used to examine subgroups and interactions	10-11, 13-14
		(*c*) Explain how missing data were addressed	10-11
		(*d*) If applicable, describe analytical methods taking account of sampling strategy	NA
		(*e*) Describe any sensitivity analyses	13-14
**Results**			
Participants	13*	(a) Report numbers of individuals at each stage of study—eg numbers potentially eligible, examined for eligibility, confirmed eligible, included in the study, completing follow-up, and analysed	7, 11
		(b) Give reasons for non-participation at each stage	7, 11
		(c) Consider use of a flow diagram	NA
Descriptive data	14*	(a) Give characteristics of study participants (eg demographic, clinical, social) and information on exposures and potential confounders	17
		(b) Indicate number of participants with missing data for each variable of interest	17
Outcome data	15*	Report numbers of outcome events or summary measures	17

For peer review only

Main results	16	(*a*) Give unadjusted estimates and, if applicable, confounder-adjusted estimates and their precision (eg, 95% confidence interval). Make clear which confounders were adjusted for and why they were included	17-19
		(*b*) Report category boundaries when continuous variables were categorized	17
		(*c*) If relevant, consider translating estimates of relative risk into absolute risk for a meaningful time period	NA
Other analyses	17	Report other analyses done—eg analyses of subgroups and interactions, and sensitivity analyses	13-14
**Discussion**			
Key results	18	Summarise key results with reference to study objectives	14
Limitations	19	Discuss limitations of the study, taking into account sources of potential bias or imprecision. Discuss both direction and magnitude of any potential bias	15
Interpretation	20	Give a cautious overall interpretation of results considering objectives, limitations, multiplicity of analyses, results from similar studies, and other relevant evidence	15-16
Generalisability	21	Discuss the generalisability (external validity) of the study results	15-16
**Other information**			
Funding	22	Give the source of funding and the role of the funders for the present study and, if applicable, for the original study on which the present article is based	20

*Give information separately for exposed and unexposed groups.

Note: An Explanation and Elaboration article discusses each checklist item and gives methodological background and published examples of transparent reporting. The STROBE checklist is best used in conjunction with this article (freely available on the Web sites of PLoS Medicine at http://www.plosmedicine.org/, Annals of Internal Medicine at http://www.annals.org/, and Epidemiology at http://www.epidem.com/). Information on the STROBE Initiative is available at www.strobe-statement.org.

For peer review only

BMJ Open

Psychological correlates of free colorectal cancer screening uptake in a Scottish sample: A cross-sectional observational study

Journal:	*BMJ Open*
Manuscript ID	bmjopen-2020-042210.R1
Article Type:	Original research
Date Submitted by the Author:	29-Apr-2021
Complete List of Authors:	Fawns-Ritchie, Chloe; The University of Edinburgh<br>Miller, Christopher; University of Stirling, Department of Psychology<br>van der Pol, Marjon; University of Aberdeen, Health Economics Research Unit<br>Douglas, Elaine; University of Stirling, Division of Economics<br>Bell, David; University of Stirling, Division of Economics<br>O'carroll, Ronan; University of Stirling, Division of Psychology<br>Deary, Ian; The University of Edinburgh, Department of Psychology
**Primary Subject Heading**:	Epidemiology
Secondary Subject Heading:	Public health
Keywords:	EPIDEMIOLOGY, PUBLIC HEALTH, STATISTICS & RESEARCH METHODS

SCHOLARONE™
Manuscripts

I, the Submitting Author has the right to grant and does grant on behalf of all authors of the Work (as defined in the below author licence), an exclusive licence and/or a non-exclusive licence for contributions from authors who are: i) UK Crown employees; ii) where BMJ has agreed a CC-BY licence shall apply, and/or iii) in accordance with the terms applicable for US Federal Government officers or employees acting as part of their official duties; on a worldwide, perpetual, irrevocable, royalty-free basis to BMJ Publishing Group Ltd ("BMJ") its licensees and where the relevant Journal is co-owned by BMJ to the co-owners of the Journal, to publish the Work in this journal and any other BMJ products and to exploit all rights, as set out in our licence.

The Submitting Author accepts and understands that any supply made under these terms is made by BMJ to the Submitting Author unless you are acting as an employee on behalf of your employer or a postgraduate student of an affiliated institution which is paying any applicable article publishing charge ("APC") for Open Access articles. Where the Submitting Author wishes to make the Work available on an Open Access basis (and intends to pay the relevant APC), the terms of reuse of such Open Access shall be governed by a Creative Commons licence – details of these licences and which Creative Commons licence will apply to this Work are set out in our licence referred to above.

Other than as permitted in any relevant BMJ Author's Self Archiving Policies, I confirm this Work has not been accepted for publication elsewhere, is not being considered for publication elsewhere and does not duplicate material already published. I confirm all authors consent to publication of this Work and authorise the granting of this licence.

For peer review only

Psychological correlates of free colorectal cancer screening uptake in a Scottish sample: A cross-sectional observational study

Chloe Fawns-Ritchie[1,2], Christopher B. Miller[3], Marjon van der Pol[4], Elaine Douglas[5], David Bell[5], Ronan E. O'Carroll[3]*, and Ian J. Deary[1,2]*

[1]Centre for Cognitive Ageing and Cognitive Epidemiology, University of Edinburgh, Edinburgh, UK

[2]Department of Psychology, University of Edinburgh, Edinburgh, UK

[3]Division of Psychology, University of Stirling, Stirling, UK

[4]Health Economics Research Unit, University of Aberdeen, Aberdeen, UK

[5]Division of Economics, University of Stirling, Stirling, UK

*These authors contributed equally

Corresponding author: Chloe Fawns-Ritchie, Department of Psychology, University of Edinburgh, Edinburgh, Scotland, UK, EH8 9JZ.

Telephone: (+44) 0131 650 8317

Email: c.fawns-ritchie@ed.ac.uk

Word count: 4594

For peer review only - http://bmjopen.bmj.com/site/about/guidelines.xhtml

For peer review only

ABSTRACT

Objectives Colorectal cancer (CRC) screening uptake in Scotland is 56%. This study examined whether psychological factors were associated with CRC screening uptake.

Design Cross-sectional observational study.

Setting This study used data from the Healthy AGeing In Scotland (HAGIS) pilot study, a study designed to be representative of Scottish adults aged 50 years and older.

Participants 908 (505 female) Scottish adults aged 50 to 80 years (mean age=65.84, SD=8.24), who took part in the HAGIS study (2016-2017).

Primary and secondary outcome measures Self-reported participation in CRC screening was the outcome measure. Logistic regression was used to test whether scores on measures of health literacy, cognitive ability, risk aversion, time preference (e.g., present-oriented or future-oriented), and personality were associated with CRC screening when these psychological factors were entered individually and simultaneously in the same model.

Results Controlling for age, age-squared, sex, living arrangement, and sex*living arrangement, a one-point increase in risk aversion (OR=0.66, 95% CI 0.51 to 0.85), and present-orientation (OR=0.86, 0.80 to 0.94) was associated with reduced odds of screening. Higher scores on health literacy (OR per one-point increase=1.20, 1.09 to 1.31), cognitive ability (OR per SD increase=1.51, 1.25 to 1.81), and the intellect personality trait (OR per one-point increase=1.05, 1.01 to 1.09) were associated with increased odds of screening. Higher risk aversion was the only psychological variable that was associated with CRC screening participation when all psychological variables were entered in the same models, and remained associated with CRC screening when additionally adjusting for deprivation and education. A backward elimination model retained two psychological variables as correlates of CRC screening; risk aversion and cognitive ability.

For peer review only

Conclusion Individuals who are more risk averse are less likely to participate in free, home CRC screening.

Keywords: Cancer screening, cognitive ability, personality, health literacy, risk aversion, time preference

For peer review only

Strengths and limitations of this study

- This study used data from the Healthy AGeing In Scotland (HAGIS) pilot study, which is a study designed to be representative of Scottish adults aged 50 years and older.
- A wide range of psychological characteristics were measured which allowed us to investigate whether each psychological characteristic was associated with colorectal cancer screening independent of the other psychological characteristics measured.
- The outcome measure used was whether participants have ever completed a colorectal screening test, and we did not examine whether participants regularly take part in colorectal cancer screening.

For peer review only

For peer review only - http://bmjopen.bmj.com/site/about/guidelines.xhtml

INTRODUCTION

Colorectal cancer (CRC) is the third most common cancer in Scotland.[1] Screening to detect CRC using the faecal occult blood test (FOBT) has been found to reduce CRC-related mortality.[2] The Scottish Bowel Screening Programme was implemented in 2009 and involves inviting adults aged 50 to 74 years to take part in free home screening (FOBT kits are mailed to eligible individuals and returned postage free) for faecal blood every two years.[3] Uptake of CRC screening in Scotland is 56%.[3, 4] Understanding the characteristics of individuals who do not complete the FOBT are important for designing methods for improving uptake. Younger age, male sex, and lower socioeconomic status are associated with lower screening rates.[4, 5]

Psychological variables may also influence screening uptake. Many studies have used the Health Belief Model[6] to investigate whether perceptions about the susceptibility and severity of cancer and the perceived benefits and barriers to screening influence cancer screening uptake. The Health Belief Model purports that an individual will participate in a health behaviour, such as cancer screening, if they believe: 1) they are *susceptible* to the condition; 2) the condition is *severe*; 3) that there would be *benefits* to engaging in a certain health behaviour; and 4) that any benefits to the behaviour outweigh any *barriers*.[6-8] A study of 335 adults aged 50-79 years in the US, tested whether constructs of the Health Belief Model were associated with FOBT as well as other recommended CRC screening procedures in the US.[9] Specific barriers to completing the FOBT were associated with lower odds of having had a FOBT in the last 12 months; however, perceived CRC risk, perceived benefits of CRC screening in general, and perceived benefits of the FOBT were not associated with having had a FOBT in the last 12 months. The Health Belief Model has also been used to predict participation in other types of cancer screening. A review of studies using the Health Belief Model to predict uptake in mammography and pap screening found strong support that

For peer review only - http://bmjopen.bmj.com/site/about/guidelines.xhtml

perceived benefits were positively associated with screening uptake and perceived barriers were negatively associated with screening uptake; however, they found weak support for an association between perceived susceptibility and severity with screening uptake.[10]

Whereas a large number of studies have tested whether the constructs of the Health Belief Model are associated with cancer screening, the relationship between other psychological characteristics, such as cognitive ability and personality, with cancer screening is less well understood. Individuals with cognitive impairment are less likely to participate in CRC screening.[11, 12] There is some evidence that people who score higher on the personality traits extraversion and conscientiousness have higher rates of cancer screening.[11-15] The role of cognitive ability and personality in CRC screening were investigated together using a nationally representative sample of older adults from England.[15] Better cognitive ability and higher conscientiousness, when measured in the same model, were associated with higher rates of CRC screening participation after adjusting for age and sex. These associations were attenuated and non-significant when additionally adjusting for health literacy and household wealth.[15] Adequate health literacy—the ability to obtain and understand basic health information[16]—has been identified as another possible correlate of CRC screening. Whereas some studies have found that lower health literacy was associated with reduced rates of CRC screening, others have not.[17-20] Gale et al.[15] tested whether the association between health literacy and CRC screening may be partly explained by cognitive ability. The association between health literacy and CRC screening was attenuated by 40% when adjusting for cognitive ability.[15]

Other psychological characteristics that may be associated with cancer screening include time and risk preference.[21-25] Individuals who are more future-oriented may be more likely to participate in routine cancer screening because these individuals place a higher value on future benefits of screening, such as reducing the need for more invasive cancer treatment,

For peer review only - http://bmjopen.bmj.com/site/about/guidelines.xhtml

For peer review only

and a lower value on the immediate costs of screening, such as time and effort, compared to individuals who are more present-oriented.[22, 23] Future-oriented women have been found to be more likely to undergo breast cancer and cervical screening.[22-24] However, when examining the role of time preference in prostate exam attendance, more present-oriented men were more likely to have had this procedure.[22] Characteristics associated with one screening programme may not necessarily be those associated with other types of screening [26].

A study of 809 UK adults examined the role of time perspective in the association between socioeconomic inequalities and CRC screening uptake (flexible sigmoidoscopy).[25] This study did not find direct associations between high consideration of future consequences (future-orientation) and CRC screening uptake; however, the association between high socioeconomic status and CRC screening uptake was mediated by future-orientation. This study also found that future-orientation was positively associated with perceived benefits and negatively associated with perceived barriers of CRC screening, which were in turn associated with screening uptake.[25]

More risk averse individuals may be more likely to take part in screening because it can reduce the risk of ill-health by identifying cancer earlier, and therefore the treatment required is less aggressive and the prognosis is better. Alternatively, risk averse individuals may be less likely to take part in screening because they may see screening as risky.[23, 24] Positive screening results are likely to lead to medical treatments which have uncertain outcomes. Studies that have investigated the association between risk aversion and cancer screening provide some support for the latter hypothesis; that risk averse individuals are less likely to participate in cancer screening. More risk averse women have been found to be less likely to undergo regular breast cancer screening.[23] Another study[24] concluded that risk aversion

For peer review only - http://bmjopen.bmj.com/site/about/guidelines.xhtml

For peer review only

was weakly associated with reduced use of cancer screening services; however, these associations did not reach statistical significance.

Most studies have tended to examine the association between only one psychological variable, or a small number of psychological variables, with cancer screening uptake. These psychological characteristics, however, have moderate correlations with each other. Higher cognitive ability is moderately correlated with health literacy[27] and with greater future-orientation.[28] Risk aversion correlates with lower cognitive function and lower literacy.[29] Risk aversion and time preference also have small, but inconsistent, associations with conscientiousness, agreeableness and extraversion.[30] It is not clear whether risk aversion, present-orientation, health literacy, cognitive ability, and personality traits, which are moderately correlated with each other, have associations with CRC screening uptake that are independent of the other psychological characteristics. Using data from the Healthy AGeing in Scotland (HAGIS) pilot study—a study designed to be representative of middle-aged and older Scottish adults—the present study aimed to test whether risk aversion, present-orientation, cognitive ability, health literacy and personality traits, when examined simultaneously in the same model, have independent associations with self-reported participation in home CRC screening.

METHODS

Participants

Data from the HAGIS pilot study[31] were used here. To recruit participants, a random sample of 5,211 residential addresses in mainland Scotland were identified using the Postcode Address File. These addresses were screened by the National Records for Scotland to find addresses where at least one resident was aged over 50 years. A total of 3,088

For peer review only - http://bmjopen.bmj.com/site/about/guidelines.xhtml

addresses were identified with someone aged 50 years and older living in the household and these were the list of addresses fieldworkers used to recruit HAGIS participants. More information on the sampling procedure is reported elsewhere.[31] Eligible participants were those aged over 50 years, and their partners if their partner was aged over 45 years.[31] A total of 1,057 participants were recruited in 2016-2017 (Figure 1). Ethical approval was obtained from the University of Stirling Ethics, the Administrative Data Research Network (PROJ-065-HAGIS Study), and the Public Benefits and Privacy Panel (HAGIS 1516-0417). This study conformed to the principles embodied in the Declaration of Helsinki.

Face-to-face interviews were carried out in the participants' own home using Computer Aided Personal Interviewing (CAPI). Written informed consent was obtained prior to the start of the interview. Information collected during the face-to-face interview included health, cognitive function, and social circumstances. At the end of the face-to-face interview, participants were also given a self-completion questionnaire which they could complete online, on paper and return via pre-paid post, or via the CAPI immediately after the face-to-face interview. The self-completion questionnaire covered topics including health behaviours and personality. Of the 1,057 participants who completed the face-to-face interview, 705 completed the self-completion questionnaire. Data collected during the face-to-face interview and the self-completion questionnaire were used in the current analysis.

For the present report, the sample was restricted to participants aged between 50 and 80 years, as these participants would have been invited to take part in the Scottish Bowel Screening Programme. Of the 1,057 participants who completed the face-to-face interview, 917 participants we aged between 50 and 80 year.

Patient and Public Involvement

For peer review only - http://bmjopen.bmj.com/site/about/guidelines.xhtml

HAGIS participants were not involved in the development of any part of this study. The results of this study will be disseminated to participants and the public via the HAGIS website (www.hagis.scot).

Measures

CRC screening

As part of the face-to-face interview participants were asked “Have you ever completed a home testing kit for screening bowel cancer?” Responses were recorded as “yes”, “no”, “don’t know“, or “refused” to answer.

Risk aversion

Risk aversion and time preference were assessed using Multiple Price Lists (MPL); a standard method for eliciting preferences in economics.[32] A MPL with three choices was used to assess risk aversion in the self-completion questionnaire (shown in Supplementary Materials). Participants were presented with a series of choices between a certain amount of monthly income of £1,500 or taking a gamble with a 50% chance of a higher monthly income and a 50% chance of a lower monthly income. The lower monthly income increased from £1,000 in question 1 to £1,300 in question 3. Less risk averse individuals will be more likely to choose the gamble. The measure of risk aversion was the point at which the participant switched from choosing the certain monthly income of £1,500, to choosing the gamble (score range 1 to 4). Higher scores reflect more risk aversion.

Time preference - present-orientation

For peer review only - http://bmjopen.bmj.com/site/about/guidelines.xhtml

Time preference was assessed using a MPL with seven choices administered in the self-completion questionnaire (shown in Supplementary Materials) that were based on a simplified version of the Kirby Delay-Discounting task.[33] Participants had to choose between receiving £1,500 now, or a higher amount of money in one month's time. The amount received in one month's time ranged from £1,506 to £1,596. If participants are very future-oriented, they will always pick the higher amount in one month's time. The measure of time preference used here was the point at which the participant switched from choosing the money now to choosing the money in one month's time (score range 1 to 8). A higher score reflects more present-orientation.

Health literacy

Health literacy was assessed during the face-to-face interview using a popular test of health literacy, the Newest Vital Sign (NVS).[34] The NVS assesses reading and numeracy skills required to understand health information.[34] Participants were presented with a nutrition label from a container of ice cream and were asked six questions about this information. For example, one question asked participants to work out how many calories they would eat if they ate the entire container of ice cream. The score is the sum of correct answers (maximum score=6).

Cognitive ability

Five tests of cognitive function that were assessed in the face-to-face interview were used here. These tests were designed to measure verbal declarative memory (immediate and delayed word recall), executive function (categorical fluency), processing speed (letter digit substitution), crystallised ability (vocabulary), and non-verbal reasoning (matrices). Scores on

For peer review only - http://bmjopen.bmj.com/site/about/guidelines.xhtml

the five cognitive tests were entered into a principal components analysis and the score on the first unrotated component was saved and used as a measure of general cognitive ability (mean=0.00, SD=1.00). Descriptions of each cognitive test and more detail on the creation of the general cognitive ability measure are reported in the Supplementary Methods.

Personality traits

Personality was assessed as part of the self-completion questionnaire using the 50-item IPIP questionnaire which measures the Big-Five personality traits of extraversion, agreeableness, conscientiousness, emotional stability (the opposite of neuroticism), and intellect/imagination.[35] This questionnaire consists of ten items for each personality factor. Participants are presented with a statement (e.g., "I make people feel at ease") and were asked to rate how accurately each statement described them on a 5-point Likert scale (very inaccurate to very accurate). The score for each factor is the sum of these ten items.

Covariates

Age in days, sex, living arrangement, social deprivation and educational qualifications were assessed as part of the face-to-face interview. Participants who reported they were married or living with their partner as if married were categorised as cohabiting. Individuals who reported they were single, separated, divorced, or widowed were categorised as living alone. Deprivation was measured using Scottish Index of Multiple Deprivation (SIMD) quartiles,[36] which ranks participant's level of deprivation based on the amount of employment, income, health, education, housing, access and crime in the area where they live. Education was assessed by asking participants "What is the highest level of education you have completed?". Qualifications were coded as 1=primary or less, 2=O level/O grade or

For peer review only - http://bmjopen.bmj.com/site/about/guidelines.xhtml

For peer review only

equivalent, 3=Highers/sixth year studies/HNC/HND or equivalent, 4=first degree, 5=postgraduate/higher degree.

Statistical analysis

To test whether individuals who have participated in CRC screening differ from non-participants on demographic, socioeconomic and psychological variables, t-tests were performed for normally distributed variables, Mann-Whitney U tests were used for non-normally distributed variables, and χ^2 tests were used for categorical variables. Pearson correlations, rank-order correlations, and partial correlations adjusting for age and sex were calculated to examine the strength of the relationship between each of the psychological factors of interest. The correlations between covariates was also calculated.

Logistic regression was used to examine the association between psychological variables and CRC screening participation. The outcome variable was coded 1 for reporting participating in CRC screening, and 0 if not. Age, age-squared, sex, living arrangement, and an interaction between sex and living arrangement were entered as covariates into all models. An age-squared term was added as the assumption of linearity of the logit was violated. An interaction term between sex and living arrangement was included because previous research using the HAGIS data has found a significant interaction between sex and living arrangement on CRC screening uptake.[37] This study found that single men were less likely to take part in CRC screening, but there were no differences in CRC screening uptake for men living with a partner, single women or women living with a partner.[37]

To test the association of each psychological variable with CRC screening, models were run in which each of the psychological variables (risk aversion, present-orientation, health literacy, cognitive ability, and each personality trait) were entered individually. Next,

For peer review only - http://bmjopen.bmj.com/site/about/guidelines.xhtml

deprivation and qualifications were added as covariates to investigate whether the strength of the association between each psychological variable and CRC screening changed after adjustment for indicators of socioeconomic status.

To determine whether any associations between risk aversion, present-orientation, health literacy, cognitive ability and personality traits with CRC screening were independent of the other psychological characteristics, a multivariate logistic regression model was run in which all psychological variables were entered into the same model. A fully-adjusted model was then run which additionally adjusted for deprivation and qualifications. A backward elimination logistic regression was run to identify the best independent correlates of CRC screening participation. If the backward elimination model identified the same psychological variables as the models including all psychological variables entered simultaneously, this would provide additional support that this psychological characteristic was an important correlate of CRC screening participation.

Finally, we tested whether there was an interaction between risk aversion and present-orientation. Completing a CRC screening test can involve the risk of a bad outcome in the short-term, whereas not completing a CRC screening test could involve the risk of a bad outcome in the future. Models were run which included risk aversion, present-orientation, and the interaction between risk aversion and present-orientation, first adjusting for age, age-squared, sex, living arrangement, and the interaction between sex and living arrangement, and then additionally adjusting for deprivation and qualifications.

For each model, the sample size was based on the number of participants with complete data on all variables entered into the model therefore the sample size for each model varies. Imputation was not used in the current study because those who did and did not return the self-completion questionnaire may differ on the psychological variables assessed in the self-

For peer review only - http://bmjopen.bmj.com/site/about/guidelines.xhtml

completion questionnaire (e.g., conscientiousness). The models were re-run using a subsample of participants with complete data on all variables. The p-values reported for the logistic regression models have been false discovery rate (FDR) corrected to control for multiple comparisons. The FDR method controls for the number of false positive results in those tests that reach significance.

RESULTS

Of the 1,057 participants who completed the face-to-face interview, the following participants were removed before conducting analyses: participants who did not answer “yes” or “no” to the CRC screening question (no data, n=1; “don’t know”, n=6; “refused”, n=3); participants without data on age (n=3), participants aged <50 (n=7), participants aged >80 (n=126); participants without data on sex (n=1) or whose sex was recorded as transgender (n=1); participants without data on cohabiting status (n=1). We removed participants without data on CRC screening, age, sex, and living arrangement because these variables were entered into every model. After removal of these participants, the analytic sample consisted of 908 participants. A flow chart showing how the analytic sample was derived is shown in Figure 1.

The mean age of the sample was 65.85 (SD=8.23). A total of 683 (75.2%) participants had completed a home CRC screening test, whereas 225 (24.8%) had not. Participant characteristics according to screening status are shown in Table 1. Individuals who participated in CRC screening were older, more likely to be cohabiting, have higher qualifications and were less likely to live in deprived areas, compared to non-participants. Screeners were less risk averse, more future-oriented, and scored higher on health literacy, cognitive ability, emotional stability and intellect.

For peer review only - http://bmjopen.bmj.com/site/about/guidelines.xhtml

For peer review only

Pearson and rank-order correlations between psychological variables and covariates are reported in Supplementary Table 1, and partial correlations between the psychological variables controlling for age and sex are reported in Supplementary Table 2. Many of the psychological variables have moderate correlations with each other. Notable among them, higher cognitive ability was moderately correlated with higher health literacy (r=0.46, p<0.001; rho=0.45, p<0.001). Risk aversion and present-orientation were correlated positively with each other (r=0.14, p=0.002; rho=0.14, p=0.002). Cognitive ability was negatively correlated with risk aversion (r=-0.13, p=0.003; rho=-0.17, p<0.001) and present-orientation (r=-0.11, p=0.022; rho=-0.11, p<0.001). Health literacy was negatively correlated with risk aversion (r=-0.12, p=.004; rho=-0.17, p<0.001). Cognitive ability was positively correlated with all five personality traits (r=0.14 to 0.38, p<0.001; rho=0.17 to 0.39, p<0.001).

Table 2 (Model 1) shows the ORs (95% CI) for participating in CRC screening, for each psychological variable entered separately, adjusting for age, age-squared, sex, living arrangement and sex*living arrangement. More risk averse (OR=0.66, 95% CI 0.51 to 0.85) and more present-oriented (OR=0.86, 0.80 to 0.94) individuals were less likely to participate in CRC screening. Individuals with higher health literacy (OR=1.20, 1.09 to 1.31), cognitive ability (OR=1.51, 1.25 to 1.81) and intellect (OR=1.05, 1.01 to 1.09) scores were more likely to screen. When additionally adjusting for deprivation and qualifications (Table 2; Model 2), the associations between risk aversion (OR=0.71, 0.55 to 0.91), health literacy (OR=1.17, 1.06 to 1.28) and cognitive ability (OR=1.36, 1.10 to 1.67) were slightly attenuated but remained significantly associated with CRC screening participation. The associations between present-orientation (OR=0.91, 0.83 to 0.99) and intellect (OR=1.03, 0.99 to 1.07) were no longer significant.

For peer review only - http://bmjopen.bmj.com/site/about/guidelines.xhtml

Next, all of the psychological variables were entered into the same model, adjusting for age, age-squared, sex, living arrangement, and sex*living arrangement (Table 3, Model 1). The association between risk aversion and CRC screening became slightly stronger (OR=0.52, 0.36 to 0.76). Present-orientation, health literacy, cognitive ability, and the five personality traits were not associated with CRC screening participation when all psychological variables were entered simultaneously. The association between risk aversion and CRC screening remained almost unchanged when additionally adjusting for qualifications and deprivation (OR=0.53, 0.36 to 0.77; Table 3, Model 2). In this fully-adjusted model, older age was associated with increased odds of screening. The significant age-squared term suggests that this association became slightly weaker with increased age. Participants living alone, compared to those cohabiting, were less likely to screen. Although the confidence intervals for deprivation and the interaction between sex and living arrangement did not cross 1, these associations were not significant following FDR adjustment.

A backwards elimination logistic regression run from the fully-adjusted model retained two psychological variables: risk aversion (OR=0.55, 0.39 to 0.78) and cognitive ability (OR=1.42, 1.05 to 1.93). This model also retained age, age-squared, living arrangement and SIMD (Table 3; Model 3).

The interaction between risk aversion and present-orientation was non-significant (OR=1.04, 0.94 to 1.14; n=494) in a model including risk aversion, present-orientation, risk aversion*present-orientation, age, age-squared, sex, living arrangement, and sex*living arrangement. This interaction remained non-significant when additionally adjusting for deprivation and qualifications (OR=1.03, 0.94 to 1.13; n=490).

Of the 908 participants who make up the analytic sample, 384 participants had complete data on all variables. Demographic characteristics were compared between participants with and

For peer review only - http://bmjopen.bmj.com/site/about/guidelines.xhtml

without complete data (Supplementary Table 3). Those with complete data were more likely to be cohabiting, had higher qualifications, and lived in areas with less deprivation than non-completers. The models reported in Table 2 were re-run using participants with complete data (Supplementary Table 4). Using only participants with complete data (n=384), the association between risk aversion and CRC screening was slightly stronger (OR=0.51, 0.35 to 0.72; minimally adjusted) than that reported in Table 2; however, the 95% CIs largely overlap. The effect sizes for the associations between the other psychological variables with CRC screening were similar in size to those reported in Table 2. Despite similar effect sizes, the association between health literacy and intellect with CRC screening were no longer significant, possibly owing to the smaller sample size.

DISCUSSION

This study investigated the association between risk aversion, present-orientation, cognitive ability, health literacy and personality traits with CRC screening uptake, and how these associations changed when all psychological variables were measured simultaneously. Using a sample of middle-aged and older adults living in Scotland, this study found that, when examining each psychological variable separately, participants who were more risk averse and more present-oriented were less likely to have participated in CRC screening, whereas individuals who had higher health literacy, cognitive ability, and intellect were more likely to have participated in CRC screening. When entering these psychological variables simultaneously in the same model, risk averse individuals were consistently less likely to have completed a home CRC screening test, even when additionally adjusting for socioeconomic indicators. Although cognitive ability was not associated with CRC screening in the fully-adjusted model, a backwards elimination logistic regression retained cognitive

For peer review only - http://bmjopen.bmj.com/site/about/guidelines.xhtml

ability as a correlate of CRC screening participation. Time preference, health literacy, and intellect were no longer associated with CRC screening when all psychological characteristics were entered simultaneously, suggesting that these psychological variables do not have associations with CRC screening that are independent of the other psychological variables measured in the current study.

Finding that risk averse individuals were less likely to take part in CRC screening is in line with another study that reported that risk averse women were less likely to regularly undergo breast cancer screening.[23] Goldzahl[23] found that risk aversion was the most important predictor of breast cancer screening, accounting for 30% of disparity in screening regularity. The outcomes of treating cancer are uncertain. Risk averse individuals may be more sensitive to this uncertainty compared to risk seeking individuals.[23] Cancer screening can also lead to what might be perceived as risky treatments.[23, 24] Emphasising how CRC screening can reduce risk of future ill-health, e.g., by increasing survival rates and reducing the need for more invasive and uncertain cancer treatments, may encourage risk averse individuals to participate in CRC screening.[23]

The current study found some evidence that higher cognitive ability was associated with increased rates of screening. Completing a home CRC screening test may be a cognitively demanding task, and therefore individuals with lower cognitive ability might struggle to successfully complete this task. Gale et al.[15] also found some support that higher cognitive ability predicted participation in a home CRC screening test. Similar to the current study, the association between cognitive ability and CRC screening was attenuated and non-significant when concurrently measuring other psychological variables (conscientiousness, health literacy) and indicators of socioeconomic status (household wealth).[15] These results suggest that the association between cognitive ability and CRC screening participation may not be independent of other psychological and socioeconomic variables.

For peer review only - http://bmjopen.bmj.com/site/about/guidelines.xhtml

Whereas other studies have found that being future-oriented and more health literate are associated with an increased likelihood of participating in cancer screening,[18, 22, 23, 38] the present study found that present-orientation and health literacy associations were attenuated and non-significant when concurrently measuring other psychological variables. Present-orientation and health literacy may not have associations with CRC screening that are independent of these other psychological characteristics. Conscientiousness, which has been linked to increased colorectal and other cancer screening in previous research,[14, 15] did not predict CRC screening here. One limitation of the current study is that some of the measures had high levels of missing data. One-third of participants did not return the self-completion questionnaire, which included the personality assessment. Conscientiousness is characterised by being organised and industrious,[35] therefore individuals who returned the questionnaire may score higher on conscientiousness than those who did not.

This study examined the association between a range of different psychological characteristics with CRC screening, but it did not consider the four key constructs (perceived susceptibility, severity, benefits and barriers) of the Health Belief Model. In addition to these constructs, the Health Belief Model assumes that modifying factors including psychological and socioeconomic variables can influence health behaviour.[10] These modifying factors may be mediators or moderators of the association between the Health Belief Model constructs and health behaviours.[10, 25] Research should be carried out to explore the role that these psychological variables—especially risk aversion—play in the association between the Health Belief Model constructs and CRC screening participation.

This study has a number of advantages, including that the HAGIS pilot study was designed to be representative of Scottish adults aged over 50 years. However, only 42% of the analytic sample had data on all variables of interest. Participants with complete data on all variables were more likely to be cohabiting, have higher qualifications and live in less deprived areas,

For peer review only - http://bmjopen.bmj.com/site/about/guidelines.xhtml

therefore a limitation is that the sample used in the fully-adjusted model is not representative of the Scottish population. The wide range of psychological characteristics assessed in the HAGIS study is an advantage. This enabled the present study examine the role of each psychological characteristic on CRC screening when considered individually, and concurrently with other psychological measures. A limitation is that this study examined whether participants have ever screened, and not whether they regularly take part in CRC screening, which is important for detecting cancer early.[3] Future studies should investigate the psychological correlates of regularly participating in CRC screening.

In this sample of Scottish middle-aged and older adults, individuals who were risk averse were less likely to self-report having ever completed a home CRC screening test, even when adjusting for a range of other psychological and socioeconomic variables. Time preference, health literacy, cognitive ability and personality did not have associations with CRC screening participation independent of the other psychological characteristics measured. Educational materials that emphasise how CRC screening can reduce the risk of future ill-health may encourage risk averse individuals to participate in CRC screening.

For peer review only - http://bmjopen.bmj.com/site/about/guidelines.xhtml

Table 1 Participant characteristics according to colorectal cancer screening participation status

	N	Yes (n=683)	No (n=225)	Effect size	p value for a difference
Age (years), mean (SD)	908	66.51 (7.95)	63.87 (8.76)	*r*=0.136	<0.001
Sex, n (%)	908			*Φ*=0.011	0.741
Female		382 (55.9%)	123 (54.7%)		
Male		301 (44.1%)	102 (45.3%)		
Living Arrangement, n (%)	908			*Φ*=0.122	<0.001
Cohabiting		474 (69.4%)	126 (56.0%)		
Living alone		209 (30.6%)	99 (44.0%)		
Qualifications, n (%)	902			Φ_c=0.123	0.009
Primary or less		117 (17.3%)	50 (22.3%)		
O level/O grade		194 (28.6%)	81 (36.2%)		
Highers/Sixth year studies/HNC/HND		208 (30.7%)	53 (23.6%)		
First degree		90 (13.3%)	29 (12.9%)		
Postgraduate/higher degree		69 (10.2%)	11 (4.9%)		
SIMD, n (%)	908			*Φ*=0.163	<0.001
1 (Most deprived)		139 (20.4%)	73 (32.4%)		
2		136 (19.9%)	59 (26.2%)		
3		213 (31.2%)	50 (22.2%)		
4 (Least deprived)		195 (28.6%)	43 (19.1%)		
Risk aversion, n (%)	581				
1 (Risk seeking)		47 (10.3%)	8 (6.5%)		
2		46 (10.1%)	6 (4.8%)		
3		55 (12.0%)	12 (9.7%)		
4 (Risk averse)		309 (67.6%)	98 (79.0%)		
Risk aversion, mean (SD)		3.37 (1.03)	3.61 (0.85)	*r*=0.104	0.012
Present-orientation, n (%)	504				
1 (Future-oriented)		159 (39.7%)	24 (23.3%)		
2		27 (6.7%)	4 (3.9%)		
3		20 (5.0%)	2 (1.9%)		
4		34 (8.5%)	8 (7.8%)		
5		20 (5.0%)	3 (2.9%)		
6		17 (4.2%)	15 (14.6%)		
7		44 (11.0%)	21 (20.4%)		
8 (Present-oriented)		80 (20.0%)	26 (25.2%)		
Present orientation, mean (SD)		3.89 (2.89)	5.15 (2.74)	*r*=0.159	<0.001
Health literacy, mean (SD)	867	4.51 (1.68)	4.07 (1.86)	*r*=0.103	0.002
Cognitive ability, mean (SD)	790	0.06 (0.98)	-0.21 (1.06)	*g*=0.273	0.001
Extraversion, mean (SD)	575	30.64 (7.17)	30.71 (7.17)	*g*=0.009	0.929
Agreeableness, mean (SD)	566	40.18 (5.95)	39.88 (6.40)	*r*=0.010	0.804
Conscientiousness, mean (SD)	547	37.77 (6.04)	36.59 (6.63)	*g*=0.191	0.065
Emotional stability, mean (SD)	575	33.68 (7.22)	31.99 (7.97)	*g*=0.229	0.035
Intellect, mean (SD)	565	33.53 (5.61)	32.14 (5.91)	*r*=0.112	0.008

SIMD, Scottish Index of Multiple Deprivation; *r*, effect size correlation coefficient; *Φ*, Phi; Φ_c, Cramer's *V*; *g*, Hedge's *g*.

For peer review only - http://bmjopen.bmj.com/site/about/guidelines.xhtml

Table 2 Odd ratios (95% CI) for participating in colorectal cancer screening

	Model 1		Model 2	
	n	OR (95% CI)	n	OR (95% CI)
Risk aversion	581	0.66 (0.51 to 0.85)**	577	0.71 (0.55 to 0.91)*
Present-orientation	504	0.86 (0.80 to 0.94)**	500	0.91 (0.83 to 0.99)
Health literacy	867	1.20 (1.09 to 1.31)**	861	1.17 (1.06 to 1.28)**
Cognitive ability	790	1.51 (1.25 to 1.81)***	784	1.36 (1.10 to 1.67)*
Extraversion	575	0.99 (0.96 to 1.02)	571	0.99 (0.96 to 1.02)
Agreeableness	566	1.01 (0.97 to 1.04)	562	1.01 (0.97 to 1.04)
Conscientiousness	547	1.03 (1.00 to 1.07)	543	1.03 (0.99 to 1.07)
Emotional stability	575	1.03 (1.00 to 1.06)	571	1.02 (0.99 to 1.05)
Intellect	565	1.05 (1.01 to 1.09)*	561	1.03 (0.99 to 1.07)

Model 1 reports the ORs (95% CIs) for each psychological variable entered individually, adjusting for age, age-squared, sex, living arrangement and sex*living arrangement. Model 2 reports the ORs (95% CIs) when additionally adjusting for deprivation and qualifications. *p<0.05, **p<0.01, ***p<0.001. P-values are false discovery rate corrected.

For peer review only

For peer review only - http://bmjopen.bmj.com/site/about/guidelines.xhtml

Table 3 Odd ratios (95% CIs) for participating in colorectal cancer screening

	Model 1 (n=388)	Model 2 (n=384)	Model 3 (n=384)
Risk aversion	0.52 (0.36 to 0.76)**	0.53 (0.36 to 0.77)**	0.55 (0.39 to 0.78)**
Present-orientation	0.94 (0.85 to 1.04)	0.98 (0.88 to 1.08)	-
Health literacy	1.02 (0.84 to 1.24)	1.02 (0.84 to 1.25)	-
Cognitive ability	1.38 (0.97 to 1.97)	1.26 (0.86 to 1.85)	1.42 (1.05 to 1.93)
Extraversion	0.97 (0.92 to 1.01)	0.97 (0.92 to 1.01)	-
Agreeableness	0.95 (0.90 to 1.02)	0.96 (0.90 to 1.03)	-
Conscientiousness	1.04 (0.99 to 1.10)	1.04 (0.98 to 1.10)	-
Emotional stability	1.03 (0.98 to 1.07)	1.02 (0.98 to 1.07)	-
Intellect	1.02 (0.96 to 1.08)	1.01 (0.95 to 1.08)	-
Age	1.08 (1.04 to 1.12)***	1.08 (1.04 to 1.12)**	1.09 (1.05 to 1.12)***
Age^2	0.99 (0.99 to 0.99)**	0.99 (0.99 to 0.99)*	0.995 (0.991 to 0.999)*
Sex			
Female	Reference	Reference	-
Male	0.60 (0.32 to 1.11)	0.61 (0.33 to 1.15)	-
Living arrangement			
Cohabiting	Reference	Reference	Reference
Living alone	0.40 (0.22 to 0.73)*	0.43 (0.23 to 0.78)*	0.54 (0.31 to 0.95)
Sex*Living arrangement	0.24 (0.07 to 0.81)	0.24 (0.07 to 0.81)	-
Qualifications	-	1.06 (0.78 to 1.45)	-
SIMD	-	1.40 (1.05 to 1.87)	1.47 (1.05 to 1.92)*

Model 1 reports the ORs (95% CIs) for each psychological variable entered simultaneously in the same model, adjusting for age, age-squared, sex, living arrangement and sex*living arrangement. Model 2 reports the ORs (95% CIs) when additionally adjusting for deprivation and qualifications. Model 3 reports the results of a backward elimination model using all variables entered in the fully-adjusted model (Model 2).
Age^2, Age-squared; SIMD, Scottish Index of Multiple Deprivation. Higher scores on SIMD reflect less deprivation.
*p<0.05, **p<0.01, ***p<0.001. P-values are false discovery rate corrected.

For peer review only - http://bmjopen.bmj.com/site/about/guidelines.xhtml

Figure 1 Participant flow chart

For peer review only

For peer review only - http://bmjopen.bmj.com/site/about/guidelines.xhtml

Acknowledgements We thank the HAGIS participants.

Completing Interests None.

Funding This work was supported by the University of Edinburgh Centre for Cognitive Ageing and Cognitive Epidemiology, part of the cross council Lifelong Health and Wellbeing Initiative, funded by the Biotechnology and Biological Sciences Research Council (BBSRC) and Medical Research Council (MRC) (grant no MR/K026992/1). The HAGIS study (data resource) was part-funded by the National Institute of Aging (RAG044535A) in the USA and was also part-funded by the Nuffield Foundation (OPD/42197), but the views expressed are those of the authors and not necessarily those of the Foundation. MvdP is supported by the Chief Scientist Office (CSO) of the Scottish Government Health and Social Care Directorates (SGHSC). The views expressed here are those of the Unit and not necessarily those of the CSO.

Author Contributions Chloe Fawns-Ritchie contributed to the conception and design of the project, analysed the data, interpreted the data, and drafted the initial manuscript. Christopher B. Miller contributed to the conception and design of the project, analysed the data, interpreted the data, and drafted the initial manuscript. Marjon van der Pol contributed to the statistical analysis of the data, interpreted the data, and critically revised the manuscript. Elaine Douglas interpreted the data, and critically revised the manuscript. David Bell interpreted the data, and critically revised the manuscript. Ronan E. O'Carroll contributed to the conception and design of the project, interpreted the data, and critically revised the manuscript. Ian J. Deary contributed to the conception and design of the project, interpreted the data, and critically revised the manuscript.

Data Availability Statement The anonymised HAGIS pilot data will be made available via the Gateway to Global Aging Data: https://g2aging.org/

For peer review only - http://bmjopen.bmj.com/site/about/guidelines.xhtml

Ethics Statement Ethical approval was obtained from the University of Stirling Ethics, the Administrative Data Research Network (PROJ-065-HAGIS Study), and the Public Benefits and Privacy Panel (HAGIS 1516-0417).

For peer review only

For peer review only - http://bmjopen.bmj.com/site/about/guidelines.xhtml

REFERENCES

1 NHS Information Services Division. Cancer Incidence in Scotland (2016). Scotland: NHS Nationl Services Scotland; 2018. Retrieved from: https://www.isdscotland.org/Health-Topics/Cancer/Publications/2018-04-24/2018-04-24-Cancer-Incidence-Report.pdf.

2 Scholefield J, Moss S, Mangham C, Whynes D, Hardcastle J. Nottingham trial of faecal occult blood testing for colorectal cancer: a 20-year follow-up. *Gut* 2012;61(7):1036-40.

3 NHS Information Services Division. Scottish Bowel Screening Programme Statistics. Scotland: NHS National Services Scotland; 2017. Retrieved from: http://www.nsd.scot.nhs.uk/services/screening/bowelscreening/index.html.

4 Mansouri D, McMillan DC, Grant Y, Crighton EM, Horgan PG. The impact of age, sex and socioeconomic deprivation on outcomes in a colorectal cancer screening programme. *PLoS One* 2013;8(6):e66063.

5 Quyn AJ, Fraser CG, Stanners G, Carey FA, Carden C, Shaukat A, et al. Uptake trends in the Scottish Bowel Screening Programme and the influences of age, sex, and deprivation. *J Med Screen* 2017:969141317694065.

6 Rosenstock IM. Historical origins of the health belief model. *Health Education Monographs* 1974;2(4):328-35.

7 Green EC, Murphy EM, Gryboski K. The Health Belief Model. The Wiley Encyclopedia of Health Psychology2020. p. 211-4.

8 Carpenter CJ. A meta-analysis of the effectiveness of health belief model variables in predicting behavior. *Health Communications* 2010;25(8):661-9.

9 Janz NK, Wren PA, Schottenfeld D, Guire KE. Colorectal cancer screening attitudes and behavior: a population-based study. *Preventive Medicine* 2003;37(6):627-34.

For peer review only - http://bmjopen.bmj.com/site/about/guidelines.xhtml

10 Tanner-Smith EE, Brown TN. Evaluating the Health Belief Model: A critical review of studies predicting mammographic and pap screening. *Social Theory & Health* 2010;8(1):95-125.

11 Heflin MT, Oddone EZ, Pieper CF, Burchett BM, Cohen HJ. The effect of comorbid illness on receipt of cancer screening by older people. *J Am Geriatr Soc* 2002;50(10):1651-8.

12 Leung DY, Leung AY, Chi I. Breast and colorectal cancer screening and associated correlates among Chinese older women. *Asian Pac J Cancer Prev* 2012;13(1):283-7.

13 Arai S, Nakaya N, Kakizaki M, Ohmori-Matsuda K, Shimazu T, Kuriyama S, et al. Personality and gastric cancer screening attendance: a cross-sectional analysis from the Miyagi Cohort Study. *J Epidemiol* 2009;19(1):34-40.

14 Sen CK, Kumkale GT. Who does not get screened? A simple model of the complex relationships in mammogram non-attendance. *J Health Psychol* 2016;21(12):2838-50.

15 Gale CR, Deary IJ, Wardle J, Zaninotto P, Batty GD. Cognitive ability and personality as predictors of participation in a national colorectal cancer screening programme: the English Longitudinal Study of Ageing. *J Epidemiol Community Health* 2015;69(6):530-5.

16 Baker DW. The meaning and the measure of health literacy. *J Gen Intern Med* 2006;21(8):878-83.

17 Kobayashi LC, Wardle J, von Wagner C. Limited health literacy is a barrier to colorectal cancer screening in England: evidence from the English Longitudinal Study of Ageing. *Prev Med* 2014;61:100-5.

18 Solmi F, Von Wagner C, Kobayashi LC, Raine R, Wardle J, Morris S. Decomposing socio-economic inequality in colorectal cancer screening uptake in England. *Soc Sci Med* 2015;134:76-86.

For peer review only - http://bmjopen.bmj.com/site/about/guidelines.xhtml

19 Peterson NB, Dwyer KA, Mulvaney SA, Dietrich MS, Rothman RL. The influence of health literacy on colorectal cancer screening knowledge, beliefs and behavior. *J Natl Med Assoc* 2007;99(10):1105-12.

20 Oldach BR, Katz ML. Health literacy and cancer screening: a systematic review. *Patient education and counseling* 2014;94(2):149-57.

21 Bradford WD. The association between individual time preferences and health maintenance habits. *Med Decis Making* 2010;30(1):99-112.

22 Bradford WD, Zoller J, Silvestri GA. Estimating the effect of individual time preferences on the use of disease screening. *Southern Economic Journal* 2010;76(4):1005-31.

23 Goldzahl L. Contributions of risk preference, time orientation and perceptions to breast cancer screening regularity. *Soc Sci Med* 2017;185:147-57.

24 Picone G, Sloan F, Taylor D. Effects of risk and time preference and expected longevity on demand for medical tests. *J Risk Uncertain* 2004;28(1):39-53.

25 Whitaker KL, Good A, Miles A, Robb K, Wardle J, von Wagner C. Socioeconomic inequalities in colorectal cancer screening uptake: does time perspective play a role? *Health Psychol* 2011;30(6):702-9.

26 Lo SH, Waller J, Wardle J, von Wagner C. Comparing barriers to colorectal cancer screening with barriers to breast and cervical screening: a population-based survey of screening-age women in Great Britain. *J Med Screen* 2013;20(2):73-9.

27 Reeve CL, Basalik D. Is health literacy an example of construct proliferation? A conceptual and empirical evaluation of its redundancy with general cognitive ability. *Intelligence* 2014;44:93-102.

28 Shamosh NA, Gray JR. Delay discounting and intelligence: A meta-analysis. *Intelligence* 2008;36(4):289-305.

For peer review only - http://bmjopen.bmj.com/site/about/guidelines.xhtml

29 Boyle PA, Yu L, Wilson RS, Segawa E, Buchman AS, Bennett DA. Cognitive decline impairs financial and health literacy among community-based older persons without dementia. *Psychology and aging* 2013;28(3):614-24.

30 Becker A, Deckers T, Dohmen T, Falk A, Kosse F. The relationship between economic preferences and psychological personality measures. *Annu Rev Econom* 2012;4(1):453-78.

31 Douglas E, Rutherford A, Bell D. Pilot study protocol to inform a future longitudinal study of ageing using linked administrative data: Healthy AGeing in Scotland (HAGIS). *BMJ Open* 2018;8(1):e018802.

32 Andersen S, Harrison GW, Lau MI, Rutström EE. Elicitation using multiple price list formats. *Exp Econ* 2006;9(4):383-405.

33 Kirby KN, Petry NM, Bickel WK. Heroin addicts have higher discount rates for delayed rewards than non-drug-using controls. *Journal of Experimental psychology: general* 1999;128(1):78.

34 Weiss BD, Mays MZ, Martz W, Castro KM, DeWalt DA, Pignone MP, et al. Quick assessment of literacy in primary care: the newest vital sign. *Ann Fam Med* 2005;3(6):514-22.

35 Goldberg LR. The development of markers for the Big-Five factor structure. *Psychol Assess* 1992;4(1):26.

36 Scottish Government. Scottish Index of Multiple Deprivation 2016. Scotland; 2016.

37 Bell D, Douglas E. Bowel cancer screening uptake in a Scottish population. Stirling: University of Stirling; 2017. Retrieved from: https://www.hagis.scot/publications/.

38 Arnold CL, Rademaker A, Bailey SC, Esparza JM, Reynolds C, Liu D, et al. Literacy barriers to colorectal cancer screening in community clinics. *J Health Commun* 2012;17(sup3):252-64.

For peer review only - http://bmjopen.bmj.com/site/about/guidelines.xhtml

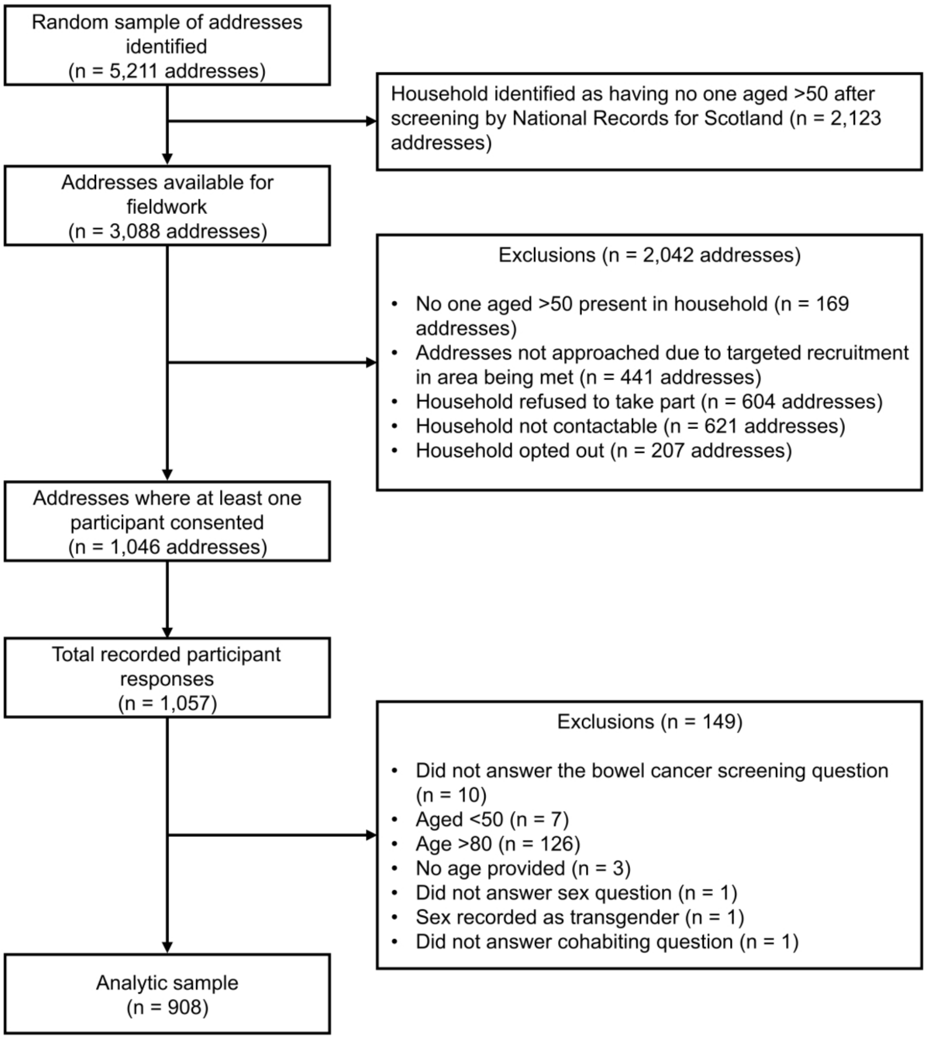

Figure 1 Participant flow chart

190x219mm (300 x 300 DPI)

Supplementary Materials for: Fawns-Ritchie et al. Psychological correlates of free colorectal cancer screening uptake in a Scottish sample

HAGIS risk aversion questions

1. Income A, which will with certainty give you a £1,500 per month for the rest of your life OR Income B which will give you a 50-50 chance of £3,000 and a 50-50 chance of £1,000 per month for the rest of your life.

Would you choose Income A or Income B?
(a) Income A
(b) Income B

2. Income A, which will with certainty give you a £1,500 per month for the rest of your life OR Income B, which will give you a 50-50 chance of £3,000 and a 50-50 chance of £1,200 per month for the rest of your life.

Would you choose Income A or Income B?
(a) Income A
(b) Income B

3. Income A, which will with certainty give you a £1,500 per month for the rest of your life OR Income B, which will give you a 50-50 chance of £3,000 and a 50-50 chance of £1,300 per month for the rest of your life.

Would you choose Income A or Income B?
(a) Income A
(b) Income B

For peer review only - http://bmjopen.bmj.com/site/about/guidelines.xhtml

HAGIS time orientation questions

1. Would you rather have £1,500 now or £1,506 a month from now?
   (a) £1,500 now
   (b) £1,506 a month from now

2. Would you rather have £1,500 now or £1,512 a month from now?
   (a) £1,500 now
   (b) £1,512 a month from now

3. Would you rather have £1,500 now or £1,518 a month from now?
   (a) £1,500 now
   (b) £1,518 a month from now

4. Would you rather have £1,500 now or £1,524 a month from now?
   (a) £1,500 now
   (b) £1,524 a month from now

5. Would you rather have £1,500 now or £1,536 a month from now?
   (a) £1,500 now
   (b) £1,536 a month from now

6. Would you rather have £1,500 now or £1,548 a month from now?
   (a) £1,500 now
   (b) £1,548 a month from now

7. Would you rather have £1,500 now or £1,596 a month from now?
   (a) £1,500 now
   (b) £1,596 a month from now

For peer review only

For peer review only - http://bmjopen.bmj.com/site/about/guidelines.xhtml

SUPPLEMENTARY METHODS

Cognitive ability

Five tests of cognitive function that were assessed during the face-to-face interview were used here. Verbal declarative memory was assessed using the word list learning test in which participants were presented with a list of 10 words and had to recall these words immediately, and again after an approximately 20 minute delay. The scores for word list learning immediate and delayed tests correlated at $r=0.66$ ($p<0.001$) therefore, the score used here is the total number of words recalled across the two tests (range 0-20). Categorical (animal) fluency, in which participants are asked to name as many animals as possible, was used to measure executive function. The score is the number of animals named in 60 seconds. The word list learning and animal fluency tests have been administered in other longitudinal studies of ageing including the Health and Retirement Study[1] and the English Longitudinal Study of Ageing.[2] Processing speed was measured using the letter digit substitution test.[3] Here, participants were presented with a piece of paper with a key pairing letters with numbers. Below the key were rows of letters with empty boxes underneath each letter. Participants were to use this key to write in the number that is paired with each letter. The score is the number of correct letter-digit matches made in 60 seconds. Two tests from the COGNITO neuropsychological test battery were administered to assess vocabulary and non-verbal fluid reasoning.[4] A shortened version of the COGNITO vocabulary test[4] was used. Participants were presented with a target word and were instructed to select which of six alternatives means the same as the target word. There were 20 items and the score is the number of correctly answered questions. COGNITO matrices[4] was used to assess non-verbal reasoning. Participants were presented with 15 matrix designs with a piece missing

For peer review only - http://bmjopen.bmj.com/site/about/guidelines.xhtml

and they were required to select the missing piece from a list of alternatives. The score is the number of correctly answered questions in 4 minutes.

To create a measure of general cognitive ability, scores on the five cognitive tests were entered into a principal components analysis. The eigenvalues and scree plot (Supplementary Figure 1) indicated that one component should be extracted. The first unrotated principal component accounted for 44% of the total variance across the five tests. The loadings for the five tests were: total word recall=0.60, animal fluency=0.73, letter digit substitution=0.70, vocabulary=0.58, matrices=0.68. The score from the first unrotated principal component was used here as a measure of general cognitive ability (mean=0.00, SD=1.00).

For peer review only

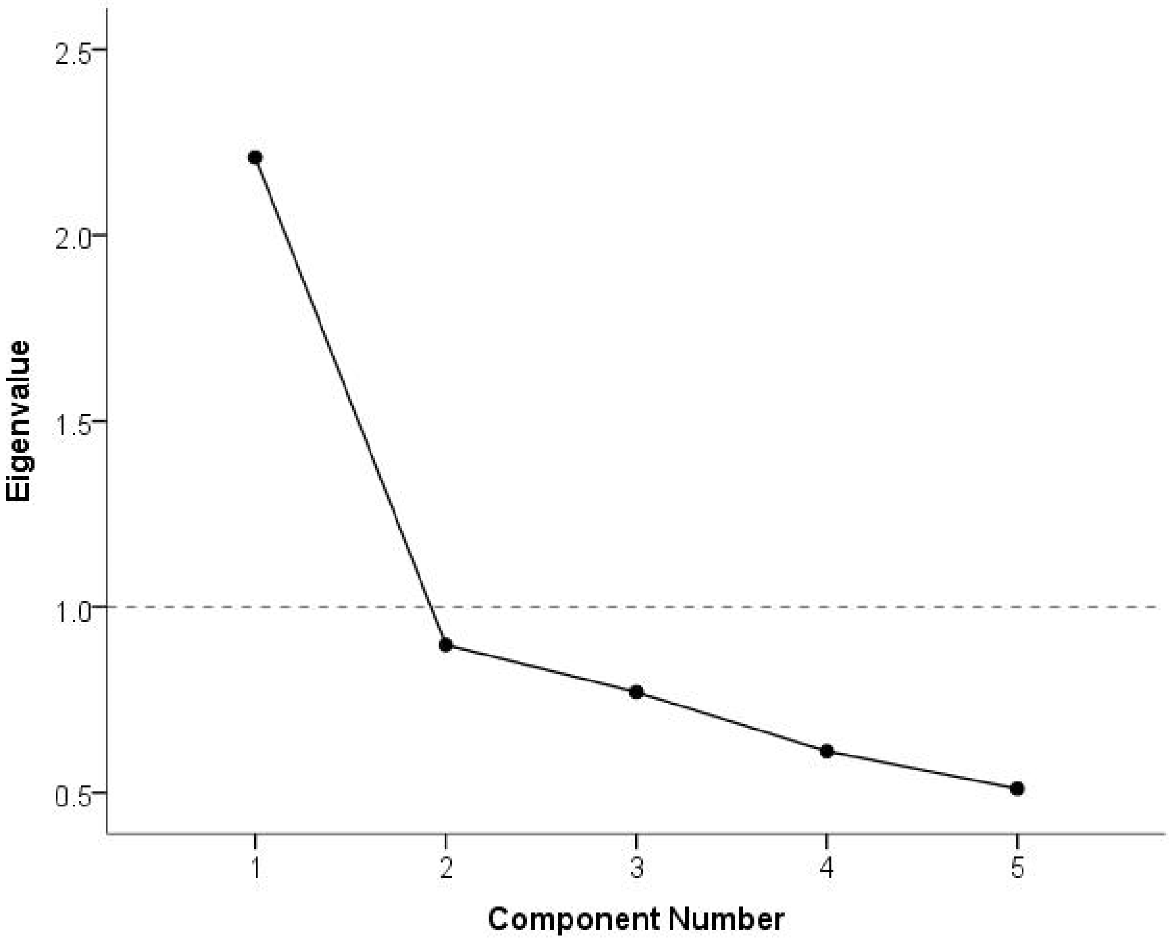

Supplementary Figure 1 Scree plot of eigenvalues from the 5 HAGIS cognitive tests

For peer review only - http://bmjopen.bmj.com/site/about/guidelines.xhtml

Supplementary Table 1 Pearson (below the diagonal) and rank-order (above the diagonal) correlations between psychological variables and covariates

	1	2	3	4	5	6	7	8	9	10	11	12	13	14
1. Age	-	0.04	-0.04	-0.16***	0.03	0.18***	-0.01	-0.20***	-0.28***	-0.03	-0.08	-0.05	0.06	-0.08
2. Sex	0.04	-	0.18***	0.07*	0.03	-0.08	-0.11*	-0.04	-0.04	-0.04	-0.29***	-0.09*	0.05	0.06
3. Cohabiting	-0.04	0.18***	-	0.14***	0.17***	-0.10*	-0.11*	0.17***	0.15***	0.11**	0.01	0.06	0.05	0.14**
4. Qualifications	-0.14***	0.08*	0.14***	-	0.35***	-0.21***	-0.24***	0.22***	0.48***	0.00	0.03	0.09*	0.13**	0.36***
5. SIMD	0.02	0.03	0.17***	0.35***	-	-0.15***	-0.28***	0.05	0.26***	0.01	0.00	-0.02	0.04	0.13**
6. Risk aversion	0.15***	-0.09*	-0.07	-0.16***	-0.12**	-	0.14**	-0.17***	-0.17***	-0.06	0.07	0.01	0.03	-0.18***
7. Present-orientation	-0.01	-0.10*	-0.11*	-0.25***	-0.30***	0.14**	-	-0.11*	-0.11***	0.07	0.10*	0.04	0.03	-0.12**
8. Health literacy	-0.21***	-0.06	0.16***	0.22***	0.08*	-0.12**	-0.09	-	0.45***	0.11**	0.08	0.13**	0.08	0.22
9. Cognitive ability	-0.27***	-0.04	0.15***	0.49***	0.27***	-0.13**	-0.11*	0.46***	-	0.17***	0.22***	0.23***	0.18***	0.39***
10. Extraversion	-0.02	-0.05	0.11**	-0.02	0.01	-0.05	0.07	0.11**	0.14**	-	0.37***	0.20***	0.27***	0.28***
11. Agreeableness	-0.09*	-0.30***	0.02	0.03	0.01	0.11*	0.10*	0.13**	0.22***	0.35***	-	0.45***	0.25***	0.27***
12. Conscientiousness	-0.05	-0.09*	0.07	0.09*	-0.01	0.05	0.04	0.15**	0.23***	0.18***	0.44***	-	0.30***	0.30***
13. Emotional stability	0.07	0.05	0.05	0.15***	0.06	0.03	0.01	0.08	0.18***	0.28***	0.22***	0.29***	-	0.08
14. Intellect	-0.06	0.08	0.12**	0.39***	0.14**	-0.15***	-0.13**	0.26***	0.38***	0.27***	0.25	0.30***	0.08	-

SIMD, Scottish Index of Multiple Deprivation.

Sex is coded 0 for female, 1 for male. Cohabiting is coded 0 for living alone, 1 for cohabiting.

*p<0.05, **p<0.01, ***p<0.001.

For peer review only - http://bmjopen.bmj.com/site/about/guidelines.xhtml

For peer review only

Supplementary Table 2 Partial correlations controlling for age and sex between psychological variables

	1	2	3	4	5	6	7	8	9
1. Risk aversion	-								
2. Present-orientation	0.15**	-							
3. Health literacy	-0.18***	-0.11*	-						
4. Cognitive ability	-0.12*	-0.16**	0.44***	-					
5. Extraversion	-0.01	0.12*	0.06	0.14**	-				
6. Agreeableness	0.09	0.09	-0.01	0.13*	0.37***	-			
7. Conscientiousness	0.05	0.06	0.06	0.20***	0.22***	0.45***	-		
8. Emotional stability	0.03	0.03	0.1	0.23***	0.27***	0.30***	0.33***	-	
9. Intellect	-0.15**	-0.13**	0.23***	0.37***	0.27***	0.27***	0.21***	0.09	-

For peer review only

For peer review only - http://bmjopen.bmj.com/site/about/guidelines.xhtml

Supplementary Table 3 Participant characteristics according to whether they have complete data on all variables of interest (completers) or whether they had missing data (non-completers)

	N	Completers (n=384)	Non-completers (n=524)	p value for a difference
Age (years), mean (SD)	908	65.33 (8.10)	66.24 (8.31)	0.101
Sex, n (%)	908			0.953
Female		214 (55.7%)	291 (55.5%)	
Male		170 (44.3%)	233 (44.5%)	
Living Arrangement, n (%)	908			0.003
Cohabiting		275 (71.6%)	325 (62.0%)	
Living alone		109 (28.4%)	199 (38.0%)	
Qualifications, n (%)	902			<0.001
Primary or less		43 (11.2%)	124 (23.9%)	
O level/O grade		113 (29.4%)	162 (31.3%)	
Highers/Sixth year studies/HNC/HND		113 (29.4%)	148 (28.6%)	
First degree		62 (16.1%)	57 (11.0%)	
Postgraduate/higher degree		53 (13.8%)	27 (5.2%)	
SIMD, n (%)	908			0.007
1 (Most deprived)		68 (17.7%)	144 (27.5%)	
2		86 (22.4%)	109 (20.8%)	
3		121 (31.5%)	142 (27.1%)	
4 (Least deprived)		109 (28.4%)	129 (24.6%)	

SIMD, Scottish Index of Multiple Deprivation.

For peer review only

Supplementary Table 4 Odd ratios (95% CIs) for participating in colorectal cancer screening using participants with complete data (n=384)

	Model 1	Model 2
	OR (95% CI)	OR (95% CI)
Risk aversion	0.51 (0.35 to 0.72)***	0.53 (0.37 to 0.77)**
Present-orientation	0.90 (0.82 to 0.98)*	0.96 (0.87 to 1.06)
Health literacy	1.17 (0.99 to 1.37)	1.10 (0.93 to 1.31)
Cognitive ability	1.56 (1.17 to 2.08)**	1.32 (0.96 to 1.83)
Extraversion	0.98 (0.94 to 1.01)	0.98 (0.95 to 1.02)
Agreeableness	0.97 (0.92 to 1.02)	0.98 (0.93 to 1.03)
Conscientiousness	1.02 (0.98 to 1.06)	1.02 (0.98 to 1.07)
Emotional stability	1.02 (0.98 to 1.06)	1.01 (0.98 to 1.05)
Intellect	1.05 (1.00 to 1.10)	1.02 (0.97 to 1.08)

Model 1 reports the ORs (95% CIs) for each psychological variable entered individually, adjusting for age, age-squared, sex, living arrangement and sex*living arrangement. Model 2 reports the ORs (95% CIs) when additionally adjusting for deprivation and qualifications. *p<0.05, **p<0.01, ***p<0.001.

For peer review only

SUPPLEMENTARY REFERENCES

1 Sonnega A, Faul JD, Ofstedal MB*, et al.* Cohort Profile: the Health and Retirement Study (HRS). *International journal of epidemiology* 2014;**43**:576-85. doi: 10.1093/ije/dyu067

2 Steptoe A, Breeze E, Banks J*, et al.* Cohort profile: the English longitudinal study of ageing. *International journal of epidemiology* 2013;**42**:1640-8. doi: 10.1093/ije/dys168

3 van der Elst W, van Boxtel MP, van Breukelen GJ*, et al.* The Letter Digit Substitution Test: normative data for 1,858 healthy participants aged 24–81 from the Maastricht Aging Study (MAAS): influence of age, education, and sex. *J Clin Exp Neuropsychol* 2006;**28**:998-1009. doi: 10.1080/13803390591004428

4 Ritchie K, de Roquefeuil G, Ritchie C*, et al.* COGNITO: computerized assessment of information processing. *J Psychol Psychother* 2014;**4**:136. doi: 10.4172/2161-0487.1000136

For peer review only

For peer review only - http://bmjopen.bmj.com/site/about/guidelines.xhtml

STROBE Statement—Checklist of items that should be included in reports of ***cross-sectional studies***

	Item No	Recommendation	Page No
**Title and abstract**	1	(*a*) Indicate the study's design with a commonly used term in the title or the abstract	1-2
		(*b*) Provide in the abstract an informative and balanced summary of what was done and what was found	2
**Introduction**			
Background/rationale	2	Explain the scientific background and rationale for the investigation being reported	5-8
Objectives	3	State specific objectives, including any prespecified hypotheses	8
**Methods**			
Study design	4	Present key elements of study design early in the paper	8, 13-15
Setting	5	Describe the setting, locations, and relevant dates, including periods of recruitment, exposure, follow-up, and data collection	8-9
Participants	6	(*a*) Give the eligibility criteria, and the sources and methods of selection of participants	8-9
Variables	7	Clearly define all outcomes, exposures, predictors, potential confounders, and effect modifiers. Give diagnostic criteria, if applicable	10-13
Data sources/ measurement	8*	For each variable of interest, give sources of data and details of methods of assessment (measurement). Describe comparability of assessment methods if there is more than one group	10-13
Bias	9	Describe any efforts to address potential sources of bias	17-18
Study size	10	Explain how the study size was arrived at	8-9, 15
Quantitative variables	11	Explain how quantitative variables were handled in the analyses. If applicable, describe which groupings were chosen and why	13-15
Statistical methods	12	(*a*) Describe all statistical methods, including those used to control for confounding	13-15
		(*b*) Describe any methods used to examine subgroups and interactions	13-15
		(*c*) Explain how missing data were addressed	14-15
		(*d*) If applicable, describe analytical methods taking account of sampling strategy	NA
		(*e*) Describe any sensitivity analyses	13-15
**Results**			
Participants	13*	(a) Report numbers of individuals at each stage of study—eg numbers potentially eligible, examined for eligibility, confirmed eligible, included in the study, completing follow-up, and analysed	8-9, 15, Fig1
		(b) Give reasons for non-participation at each stage	8-9, 15
		(c) Consider use of a flow diagram	Fig1
Descriptive data	14*	(a) Give characteristics of study participants (eg demographic, clinical, social) and information on exposures and potential confounders	22
		(b) Indicate number of participants with missing data for each variable of interest	22
Outcome data	15*	Report numbers of outcome events or summary measures	22

For peer review only

Main results	16	(*a*) Give unadjusted estimates and, if applicable, confounder-adjusted estimates and their precision (eg, 95% confidence interval). Make clear which confounders were adjusted for and why they were included	23-24
		(*b*) Report category boundaries when continuous variables were categorized	13-15
		(*c*) If relevant, consider translating estimates of relative risk into absolute risk for a meaningful time period	NA
Other analyses	17	Report other analyses done—eg analyses of subgroups and interactions, and sensitivity analyses	17-18
**Discussion**			
Key results	18	Summarise key results with reference to study objectives	18-19
Limitations	19	Discuss limitations of the study, taking into account sources of potential bias or imprecision. Discuss both direction and magnitude of any potential bias	20-21
Interpretation	20	Give a cautious overall interpretation of results considering objectives, limitations, multiplicity of analyses, results from similar studies, and other relevant evidence	18-21
Generalisability	21	Discuss the generalisability (external validity) of the study results	18-21
**Other information**			
Funding	22	Give the source of funding and the role of the funders for the present study and, if applicable, for the original study on which the present article is based	26

*Give information separately for exposed and unexposed groups.

Note: An Explanation and Elaboration article discusses each checklist item and gives methodological background and published examples of transparent reporting. The STROBE checklist is best used in conjunction with this article (freely available on the Web sites of PLoS Medicine at http://www.plosmedicine.org/, Annals of Internal Medicine at http://www.annals.org/, and Epidemiology at http://www.epidem.com/). Information on the STROBE Initiative is available at www.strobe-statement.org.

For peer review only

For peer review only - http://bmjopen.bmj.com/site/about/guidelines.xhtml

BMJ Open

Psychological correlates of free colorectal cancer screening uptake in a Scottish sample: A cross-sectional observational study

Journal:	*BMJ Open*
Manuscript ID	bmjopen-2020-042210.R2
Article Type:	Original research
Date Submitted by the Author:	18-Oct-2021
Complete List of Authors:	Fawns-Ritchie, Chloe; The University of Edinburgh<br>Miller, Christopher; University of Stirling, Department of Psychology<br>van der Pol, Marjon; University of Aberdeen, Health Economics Research Unit<br>Douglas, Elaine; University of Stirling, Division of Economics<br>Bell, David; University of Stirling, Division of Economics<br>O'carroll, Ronan; University of Stirling, Division of Psychology<br>Deary, Ian; The University of Edinburgh, Department of Psychology
**Primary Subject Heading**:	Epidemiology
Secondary Subject Heading:	Public health
Keywords:	EPIDEMIOLOGY, PUBLIC HEALTH, STATISTICS & RESEARCH METHODS

SCHOLARONE™
Manuscripts

I, the Submitting Author has the right to grant and does grant on behalf of all authors of the Work (as defined in the below author licence), an exclusive licence and/or a non-exclusive licence for contributions from authors who are: i) UK Crown employees; ii) where BMJ has agreed a CC-BY licence shall apply, and/or iii) in accordance with the terms applicable for US Federal Government officers or employees acting as part of their official duties; on a worldwide, perpetual, irrevocable, royalty-free basis to BMJ Publishing Group Ltd ("BMJ") its licensees and where the relevant Journal is co-owned by BMJ to the co-owners of the Journal, to publish the Work in this journal and any other BMJ products and to exploit all rights, as set out in our licence.

The Submitting Author accepts and understands that any supply made under these terms is made by BMJ to the Submitting Author unless you are acting as an employee on behalf of your employer or a postgraduate student of an affiliated institution which is paying any applicable article publishing charge ("APC") for Open Access articles. Where the Submitting Author wishes to make the Work available on an Open Access basis (and intends to pay the relevant APC), the terms of reuse of such Open Access shall be governed by a Creative Commons licence – details of these licences and which Creative Commons licence will apply to this Work are set out in our licence referred to above.

Other than as permitted in any relevant BMJ Author's Self Archiving Policies, I confirm this Work has not been accepted for publication elsewhere, is not being considered for publication elsewhere and does not duplicate material already published. I confirm all authors consent to publication of this Work and authorise the granting of this licence.

For peer review only

Psychological correlates of free colorectal cancer screening uptake in a Scottish sample: A cross-sectional observational study

Chloe Fawns-Ritchie[1,2], Christopher B. Miller[3], Marjon van der Pol[4], Elaine Douglas[5], David Bell[5], Ronan E. O'Carroll[3]*, and Ian J. Deary[1,2]*

[1]Centre for Cognitive Ageing and Cognitive Epidemiology, University of Edinburgh, Edinburgh, UK

[2]Department of Psychology, University of Edinburgh, Edinburgh, UK

[3]Division of Psychology, University of Stirling, Stirling, UK

[4]Health Economics Research Unit, University of Aberdeen, Aberdeen, UK

[5]Division of Economics, University of Stirling, Stirling, UK

*These authors contributed equally

Corresponding author: Chloe Fawns-Ritchie, Department of Psychology, University of Edinburgh, Edinburgh, Scotland, UK, EH8 9JZ.

Telephone: (+44) 0131 650 8317

Email: c.fawns-ritchie@ed.ac.uk

Word count: 4605

For peer review only - http://bmjopen.bmj.com/site/about/guidelines.xhtml

ABSTRACT

Objectives Colorectal cancer (CRC) screening uptake in Scotland is 56%. This study examined whether psychological factors were associated with CRC screening uptake.

Design Cross-sectional observational study.

Setting This study used data from the Healthy AGeing In Scotland (HAGIS) pilot study, a study designed to be representative of Scottish adults aged 50 years and older.

Participants 908 (505 female) Scottish adults aged 50 to 80 years (mean age=65.84, SD=8.24), who took part in the HAGIS study (2016-2017).

Primary and secondary outcome measures Self-reported participation in CRC screening was the outcome measure. Logistic regression was used to test whether scores on measures of health literacy, cognitive ability, risk aversion, time preference (e.g., present-oriented or future-oriented), and personality were associated with CRC screening when these psychological factors were entered individually and simultaneously in the same model.

Results Controlling for age, age-squared, sex, living arrangement, and sex*living arrangement, a one-point increase in risk aversion (OR=0.66, 95% CI 0.51 to 0.85), and present-orientation (OR=0.86, 0.80 to 0.94) was associated with reduced odds of screening. Higher scores on health literacy (OR per one-point increase=1.20, 1.09 to 1.31), cognitive ability (OR per SD increase=1.51, 1.25 to 1.81), and the intellect personality trait (OR per one-point increase=1.05, 1.01 to 1.09) were associated with increased odds of screening. Higher risk aversion was the only psychological variable that was associated with CRC screening participation when all psychological variables were entered in the same models, and remained associated with CRC screening when additionally adjusting for deprivation and education. A backward elimination model retained two psychological variables as correlates of CRC screening; risk aversion and cognitive ability.

For peer review only - http://bmjopen.bmj.com/site/about/guidelines.xhtml

Conclusion Individuals who are more risk averse are less likely to participate in free, home CRC screening.

Keywords: Cancer screening, cognitive ability, personality, health literacy, risk aversion, time preference

For peer review only

For peer review only - http://bmjopen.bmj.com/site/about/guidelines.xhtml

Strengths and limitations of this study

- This study used data from the Healthy AGeing In Scotland (HAGIS) pilot study, which is a study designed to be representative of Scottish adults aged 50 years and older.
- A wide range of psychological characteristics were measured which allowed us to investigate whether each psychological characteristic was associated with colorectal cancer screening independent of the other psychological characteristics measured.
- The outcome measure used was whether participants have ever completed a colorectal screening test, and we did not examine whether participants regularly take part in colorectal cancer screening.

For peer review only

INTRODUCTION

Colorectal cancer (CRC) is the third most common cancer in Scotland.[1] Screening to detect CRC using the faecal occult blood test (FOBT) has been found to reduce CRC-related mortality.[2] The Scottish Bowel Screening Programme was implemented in 2009 and involves inviting adults aged 50 to 74 years to take part in free home screening (FOBT kits are mailed to eligible individuals and returned postage free) for faecal blood every two years.[3] Uptake of CRC screening in Scotland is 56%.[3, 4] Understanding the characteristics of individuals who do not complete the FOBT are important for designing methods for improving uptake. Younger age, male sex, and lower socioeconomic status are associated with lower screening rates.[4, 5]

Psychological variables may also influence screening uptake. Many studies have used the Health Belief Model[6] to investigate whether perceptions about the susceptibility and severity of cancer and the perceived benefits and barriers to screening influence cancer screening uptake. The Health Belief Model purports that an individual will participate in a health behaviour, such as cancer screening, if they believe: 1) they are *susceptible* to the condition; 2) the condition is *severe*; 3) that there would be *benefits* to engaging in a certain health behaviour; and 4) that any benefits to the behaviour outweigh any *barriers*.[6-8] A study of 335 adults aged 50-79 years in the US, tested whether constructs of the Health Belief Model were associated with FOBT as well as other recommended CRC screening procedures in the US.[9] Specific barriers to completing the FOBT were associated with lower odds of having had a FOBT in the last 12 months; however, perceived CRC risk, perceived benefits of CRC screening in general, and perceived benefits of the FOBT were not associated with having had a FOBT in the last 12 months. The Health Belief Model has also been used to predict participation in other types of cancer screening. A review of studies using the Health Belief Model to predict uptake in mammography and pap screening found strong support that

For peer review only - http://bmjopen.bmj.com/site/about/guidelines.xhtml

perceived benefits were positively associated with screening uptake and perceived barriers were negatively associated with screening uptake; however, they found weak support for an association between perceived susceptibility and severity with screening uptake.[10]

Whereas a large number of studies have tested whether the constructs of the Health Belief Model are associated with cancer screening, the relationship between other psychological characteristics, such as cognitive ability and personality, with cancer screening is less well understood. Individuals with cognitive impairment are less likely to participate in CRC screening.[11, 12] There is some evidence that people who score higher on the personality traits extraversion and conscientiousness have higher rates of cancer screening.[11-15] The role of cognitive ability and personality in CRC screening were investigated together using a nationally representative sample of older adults from England.[15] Better cognitive ability and higher conscientiousness, when measured in the same model, were associated with higher rates of CRC screening participation after adjusting for age and sex. These associations were attenuated and non-significant when additionally adjusting for health literacy and household wealth.[15] Adequate health literacy—the ability to obtain and understand basic health information[16]—has been identified as another possible correlate of CRC screening. Whereas some studies have found that lower health literacy was associated with reduced rates of CRC screening, others have not.[17-20] Gale et al.[15] tested whether the association between health literacy and CRC screening may be partly explained by cognitive ability. The association between health literacy and CRC screening was attenuated by 40% when adjusting for cognitive ability.[15]

Other psychological characteristics that may be associated with cancer screening include time and risk preference.[21-25] Individuals who are more future-oriented may be more likely to participate in routine cancer screening because these individuals place a higher value on future benefits of screening, such as reducing the need for more invasive cancer treatment,

For peer review only - http://bmjopen.bmj.com/site/about/guidelines.xhtml

and a lower value on the immediate costs of screening, such as time and effort, compared to individuals who are more present-oriented.[22, 23] Future-oriented women have been found to be more likely to undergo breast cancer and cervical screening.[22-24] However, when examining the role of time preference in prostate exam attendance, more present-oriented men were more likely to have had this procedure.[22] Characteristics associated with one screening programme may not necessarily be those associated with other types of screening [26].

A study of 809 UK adults examined the role of time perspective in the association between socioeconomic inequalities and CRC screening uptake (flexible sigmoidoscopy).[25] This study did not find direct associations between high consideration of future consequences (future-orientation) and CRC screening uptake; however, the association between high socioeconomic status and CRC screening uptake was mediated by future-orientation. This study also found that future-orientation was positively associated with perceived benefits and negatively associated with perceived barriers of CRC screening, which were in turn associated with screening uptake.[25]

More risk averse individuals may be more likely to take part in screening because it can reduce the risk of ill-health by identifying cancer earlier, and therefore the treatment required is less aggressive and the prognosis is better. Alternatively, risk averse individuals may be less likely to take part in screening because they may see screening as risky.[23, 24] Positive screening results are likely to lead to medical treatments which have uncertain outcomes. Studies that have investigated the association between risk aversion and cancer screening provide some support for the latter hypothesis; that risk averse individuals are less likely to participate in cancer screening. More risk averse women have been found to be less likely to undergo regular breast cancer screening.[23] Another study[24] concluded that risk aversion

For peer review only - http://bmjopen.bmj.com/site/about/guidelines.xhtml

For peer review only

was weakly associated with reduced use of cancer screening services; however, these associations did not reach statistical significance.

Most studies have tended to examine the association between only one psychological variable, or a small number of psychological variables, with cancer screening uptake. These psychological characteristics, however, have moderate correlations with each other. Higher cognitive ability is moderately correlated with health literacy[27] and with greater future-orientation.[28] Risk aversion correlates with lower cognitive function and lower literacy.[29] Risk aversion and time preference also have small, but inconsistent, associations with conscientiousness, agreeableness and extraversion.[30] It is not clear whether risk aversion, present-orientation, health literacy, cognitive ability, and personality traits, which are moderately correlated with each other, have associations with CRC screening uptake that are independent of the other psychological characteristics. Using data from the Healthy AGeing in Scotland (HAGIS) pilot study—a study designed to be representative of middle-aged and older Scottish adults—the present study aimed to test whether risk aversion, present-orientation, cognitive ability, health literacy and personality traits, when examined simultaneously in the same model, have independent associations with self-reported participation in home CRC screening.

METHODS

Participants

Data from the HAGIS pilot study[31] were used here. To recruit participants, a random sample of 5,211 residential addresses in mainland Scotland were identified using the Postcode Address File. These addresses were screened by the National Records for Scotland to find addresses where at least one resident was aged over 50 years. A total of 3,088

For peer review only - http://bmjopen.bmj.com/site/about/guidelines.xhtml

addresses were identified with someone aged 50 years and older living in the household and these were the list of addresses fieldworkers used to recruit HAGIS participants. More information on the sampling procedure is reported elsewhere.[31] Eligible participants for the HAGIS study were those aged over 50 years, and their partners if their partner was aged over 45 years.[31] A total of 1,057 participants took part in the HAGIS study in 2016-2017 and form the HAGIS sample (Figure 1). Ethical approval was obtained from the University of Stirling Ethics, the Administrative Data Research Network (PROJ-065-HAGIS Study), and the Public Benefits and Privacy Panel (HAGIS 1516-0417). This study conformed to the principles embodied in the Declaration of Helsinki.

Face-to-face interviews were carried out in the participants' own home using Computer Aided Personal Interviewing (CAPI). Written informed consent was obtained prior to the start of the interview. Information collected during the face-to-face interview included health, cognitive function, and social circumstances. At the end of the face-to-face interview, participants were also given a self-completion questionnaire which they could complete online, on paper and return via pre-paid post, or via the CAPI immediately after the face-to-face interview. The self-completion questionnaire covered topics including health behaviours and personality. Of the 1,057 participants who took part in HAGIS, 705 completed the self-completion questionnaire. Data collected during the face-to-face interview and the self-completion questionnaire were used in the current analysis.

Patient and Public Involvement

HAGIS participants were not involved in the development of any part of this study. The results of this study will be disseminated to participants and the public via the HAGIS website (www.hagis.scot).

For peer review only - http://bmjopen.bmj.com/site/about/guidelines.xhtml

Measures

CRC screening

As part of the face-to-face interview participants were asked “Have you ever completed a home testing kit for screening bowel cancer?” Responses were recorded as “yes”, “no”, “don’t know“, or “refused” to answer.

Risk aversion

Risk aversion and time preference were assessed using Multiple Price Lists (MPL); a standard method for eliciting preferences in economics.[32] A MPL with three choices was used to assess risk aversion in the self-completion questionnaire (shown in Supplementary Materials). Participants were presented with a series of choices between a certain amount of monthly income of £1,500 or taking a gamble with a 50% chance of a higher monthly income and a 50% chance of a lower monthly income. The lower monthly income increased from £1,000 in question 1 to £1,300 in question 3. Less risk averse individuals will be more likely to choose the gamble. The measure of risk aversion was the point at which the participant switched from choosing the certain monthly income of £1,500, to choosing the gamble (score range 1 to 4). Higher scores reflect more risk aversion.

Time preference - present-orientation

Time preference was assessed using a MPL with seven choices administered in the self-completion questionnaire (shown in Supplementary Materials) that were based on a simplified version of the Kirby Delay-Discounting task.[33] Participants had to choose

For peer review only - http://bmjopen.bmj.com/site/about/guidelines.xhtml

between receiving £1,500 now, or a higher amount of money in one month's time. The amount received in one month's time ranged from £1,506 to £1,596. If participants are very future-oriented, they will always pick the higher amount in one month's time. The measure of time preference used here was the point at which the participant switched from choosing the money now to choosing the money in one month's time (score range 1 to 8). A higher score reflects more present-orientation.

Health literacy

Health literacy was assessed during the face-to-face interview using a popular test of health literacy, the Newest Vital Sign (NVS).[34] The NVS assesses reading and numeracy skills required to understand health information.[34] Participants were presented with a nutrition label from a container of ice cream and were asked six questions about this information. For example, one question asked participants to work out how many calories they would eat if they ate the entire container of ice cream. The score is the sum of correct answers (maximum score=6).

Cognitive ability

Five tests of cognitive function that were assessed in the face-to-face interview were used here. These tests were designed to measure verbal declarative memory (immediate and delayed word recall), executive function (categorical fluency), processing speed (letter digit substitution), crystallised ability (vocabulary), and non-verbal reasoning (matrices). Scores on the five cognitive tests were entered into a principal components analysis and the score on the first unrotated component was saved and used as a measure of general cognitive ability

For peer review only

(mean=0.00, SD=1.00). Descriptions of each cognitive test and more detail on the creation of the general cognitive ability measure are reported in the Supplementary Methods.

Personality traits

Personality was assessed as part of the self-completion questionnaire using the 50-item IPIP questionnaire which measures the Big-Five personality traits of extraversion, agreeableness, conscientiousness, emotional stability (the opposite of neuroticism), and intellect/imagination.[35] This questionnaire consists of ten items for each personality factor. Participants are presented with a statement (e.g., "I make people feel at ease") and were asked to rate how accurately each statement described them on a 5-point Likert scale (very inaccurate to very accurate). The score for each factor is the sum of these ten items.

Covariates

Age in days, sex, living arrangement, social deprivation and educational qualifications were assessed as part of the face-to-face interview. Participants who reported they were married or living with their partner as if married were categorised as cohabiting. Individuals who reported they were single, separated, divorced, or widowed were categorised as living alone. Deprivation was measured using Scottish Index of Multiple Deprivation (SIMD) quartiles,[36] which ranks participant's level of deprivation based on the amount of employment, income, health, education, housing, access and crime in the area where they live. Education was assessed by asking participants "What is the highest level of education you have completed?". Qualifications were coded as 1=primary or less, 2=O level/O grade or equivalent, 3=Highers/sixth year studies/HNC/HND or equivalent, 4=first degree, 5=postgraduate/higher degree.

For peer review only

Statistical analysis

To test whether individuals who have participated in CRC screening differ from non-participants on demographic, socioeconomic and psychological variables, t-tests were performed for normally distributed variables, Mann-Whitney U tests were used for non-normally distributed variables, and χ^2 tests were used for categorical variables. Pearson correlations, rank-order correlations, and partial correlations adjusting for age and sex were calculated to examine the strength of the relationship between each of the psychological factors of interest. The correlations between covariates was also calculated.

Logistic regression was used to examine the association between psychological variables and CRC screening participation. The outcome variable was coded 1 for reporting participating in CRC screening, and 0 if not. Age, age-squared, sex, living arrangement, and an interaction between sex and living arrangement were entered as covariates into all models. An age-squared term was added as the assumption of linearity of the logit was violated. An interaction term between sex and living arrangement was included because previous research using the HAGIS data has found a significant interaction between sex and living arrangement on CRC screening uptake.[37] This study found that single men were less likely to take part in CRC screening, but there were no differences in CRC screening uptake for men living with a partner, single women or women living with a partner.[37]

To test the association of each psychological variable with CRC screening, models were run in which each of the psychological variables (risk aversion, present-orientation, health literacy, cognitive ability, and each personality trait) were entered individually. Next, deprivation and qualifications were added as covariates to investigate whether the strength of

For peer review only - http://bmjopen.bmj.com/site/about/guidelines.xhtml

the association between each psychological variable and CRC screening changed after adjustment for indicators of socioeconomic status.

To determine whether any associations between risk aversion, present-orientation, health literacy, cognitive ability and personality traits with CRC screening were independent of the other psychological characteristics, a multivariate logistic regression model was run in which all psychological variables were entered into the same model. A fully-adjusted model was then run which additionally adjusted for deprivation and qualifications. A backward elimination logistic regression was run to identify the best independent correlates of CRC screening participation. If the backward elimination model identified the same psychological variables as the models including all psychological variables entered simultaneously, this would provide additional support that this psychological characteristic was an important correlate of CRC screening participation.

Finally, we tested whether there was an interaction between risk aversion and present-orientation. Completing a CRC screening test can involve the risk of a bad outcome in the short-term, whereas not completing a CRC screening test could involve the risk of a bad outcome in the future. Models were run which included risk aversion, present-orientation, and the interaction between risk aversion and present-orientation, first adjusting for age, age-squared, sex, living arrangement, and the interaction between sex and living arrangement, and then additionally adjusting for deprivation and qualifications.

For each model, the sample size was based on the number of participants with complete data on all variables entered into the model therefore the sample size for each model varies. Imputation was not used in the current study because those who did and did not return the self-completion questionnaire may differ on the psychological variables assessed in the self-completion questionnaire (e.g., conscientiousness). The models were re-run using a

For peer review only - http://bmjopen.bmj.com/site/about/guidelines.xhtml

subsample of participants with complete data on all variables. The p-values reported for the logistic regression models have been false discovery rate (FDR) corrected to control for multiple comparisons. The FDR method controls for the number of false positive results in those tests that reach significance.

RESULTS

Of the 1,057 participants who took part in HAGIS, the following participants were removed before conducting the current analyses: participants who did not answer "yes" or "no" to the CRC screening question (no data, n=1; "don't know", n=6; "refused", n=3); participants without data on age (n=3), participants aged <50 (n=7), participants aged >80 (n=126); participants without data on sex (n=1) or whose sex was recorded as transgender (n=1); participants without data on cohabiting status (n=1). We removed participants without data on CRC screening, age, sex, and living arrangement because these variables were entered into every model. We removed participants aged under 50 and over 80 because these participants would not have been invited to take part in the Scottish Bowel Cancer Screening Programme. After removal of these participants, the analytic sample consisted of 908 participants. A flow chart showing how the analytic sample was derived is shown in Figure 1.

The mean age of the sample was 65.85 (SD=8.23). A total of 683 (75.2%) participants had completed a home CRC screening test, whereas 225 (24.8%) had not. Participant characteristics according to screening status are shown in Table 1. Individuals who participated in CRC screening were older, more likely to be cohabiting, have higher qualifications and were less likely to live in deprived areas, compared to non-participants. Screeners were less risk averse, more future-oriented, and scored higher on health literacy, cognitive ability, emotional stability and intellect.

For peer review only - http://bmjopen.bmj.com/site/about/guidelines.xhtml

Pearson and rank-order correlations between psychological variables and covariates are reported in Supplementary Table 1, and partial correlations between the psychological variables controlling for age and sex are reported in Supplementary Table 2. Many of the psychological variables have moderate correlations with each other. Notable among them, higher cognitive ability was moderately correlated with higher health literacy (r=0.46, p<0.001; rho=0.45, p<0.001). Risk aversion and present-orientation were correlated positively with each other (r=0.14, p=0.002; rho=0.14, p=0.002). Cognitive ability was negatively correlated with risk aversion (r=-0.13, p=0.003; rho=-0.17, p<0.001) and present-orientation (r=-0.11, p=0.022; rho=-0.11, p<0.001). Health literacy was negatively correlated with risk aversion (r=-0.12, p=.004; rho=-0.17, p<0.001). Cognitive ability was positively correlated with all five personality traits (r=0.14 to 0.38, p<0.001; rho=0.17 to 0.39, p<0.001).

Table 2 (Model 1) shows the ORs (95% CI) for participating in CRC screening, for each psychological variable entered separately, adjusting for age, age-squared, sex, living arrangement and sex*living arrangement. More risk averse (OR=0.66, 95% CI 0.51 to 0.85) and more present-oriented (OR=0.86, 0.80 to 0.94) individuals were less likely to participate in CRC screening. Individuals with higher health literacy (OR=1.20, 1.09 to 1.31), cognitive ability (OR=1.51, 1.25 to 1.81) and intellect (OR=1.05, 1.01 to 1.09) scores were more likely to screen. When additionally adjusting for deprivation and qualifications (Table 2; Model 2), the associations between risk aversion (OR=0.71, 0.55 to 0.91), health literacy (OR=1.17, 1.06 to 1.28) and cognitive ability (OR=1.36, 1.10 to 1.67) were slightly attenuated but remained significantly associated with CRC screening participation. The associations between present-orientation (OR=0.91, 0.83 to 0.99) and intellect (OR=1.03, 0.99 to 1.07) were no longer significant.

For peer review only - http://bmjopen.bmj.com/site/about/guidelines.xhtml

Next, all of the psychological variables were entered into the same model, adjusting for age, age-squared, sex, living arrangement, and sex*living arrangement (Table 3, Model 1). The association between risk aversion and CRC screening became slightly stronger (OR=0.52, 0.36 to 0.76). Present-orientation, health literacy, cognitive ability, and the five personality traits were not associated with CRC screening participation when all psychological variables were entered simultaneously. The association between risk aversion and CRC screening remained almost unchanged when additionally adjusting for qualifications and deprivation (OR=0.53, 0.36 to 0.77; Table 3, Model 2). In this fully-adjusted model, older age was associated with increased odds of screening. The significant age-squared term suggests that this association became slightly weaker with increased age. Participants living alone, compared to those cohabiting, were less likely to screen. Although the confidence intervals for deprivation and the interaction between sex and living arrangement did not cross 1, these associations were not significant following FDR adjustment.

A backwards elimination logistic regression run from the fully-adjusted model retained two psychological variables: risk aversion (OR=0.55, 0.39 to 0.78) and cognitive ability (OR=1.42, 1.05 to 1.93). This model also retained age, age-squared, living arrangement and SIMD (Table 3; Model 3).

The interaction between risk aversion and present-orientation was non-significant (OR=1.04, 0.94 to 1.14; n=494) in a model including risk aversion, present-orientation, risk aversion*present-orientation, age, age-squared, sex, living arrangement, and sex*living arrangement. This interaction remained non-significant when additionally adjusting for deprivation and qualifications (OR=1.03, 0.94 to 1.13; n=490).

Of the 908 participants who make up the analytic sample, 524 individuals had missing data on one or more of the predictor variables and covariates, including 302 participants who did

For peer review only - http://bmjopen.bmj.com/site/about/guidelines.xhtml

not complete the self-completion questionnaire (Figure 1). Demographic characteristics were compared between participants with and without complete data (Supplementary Table 3). Those with complete data were more likely to be cohabiting, had higher qualifications, and lived in areas with less deprivation than non-completers. The models reported in Table 2 were re-run using participants with complete data (Supplementary Table 4). Using only participants with complete data (n=384), the association between risk aversion and CRC screening was slightly stronger (OR=0.51, 0.35 to 0.72; minimally adjusted) than that reported in Table 2; however, the 95% CIs largely overlap. The effect sizes for the associations between the other psychological variables with CRC screening were similar in size to those reported in Table 2. Despite similar effect sizes, the association between health literacy and intellect with CRC screening were no longer significant, possibly owing to the smaller sample size.

DISCUSSION

This study investigated the association between risk aversion, present-orientation, cognitive ability, health literacy and personality traits with CRC screening uptake, and how these associations changed when all psychological variables were measured simultaneously. Using a sample of middle-aged and older adults living in Scotland, this study found that, when examining each psychological variable separately, participants who were more risk averse and more present-oriented were less likely to have participated in CRC screening, whereas individuals who had higher health literacy, cognitive ability, and intellect were more likely to have participated in CRC screening. When entering these psychological variables simultaneously in the same model, risk averse individuals were consistently less likely to have completed a home CRC screening test, even when additionally adjusting for

For peer review only - http://bmjopen.bmj.com/site/about/guidelines.xhtml

socioeconomic indicators. Although cognitive ability was not associated with CRC screening in the fully-adjusted model, a backwards elimination logistic regression retained cognitive ability as a correlate of CRC screening participation. Time preference, health literacy, and intellect were no longer associated with CRC screening when all psychological characteristics were entered simultaneously, suggesting that these psychological variables do not have associations with CRC screening that are independent of the other psychological variables measured in the current study.

Finding that risk averse individuals were less likely to take part in CRC screening is in line with another study that reported that risk averse women were less likely to regularly undergo breast cancer screening.[23] Goldzahl[23] found that risk aversion was the most important predictor of breast cancer screening, accounting for 30% of disparity in screening regularity. The outcomes of treating cancer are uncertain. Risk averse individuals may be more sensitive to this uncertainty compared to risk seeking individuals.[23] Cancer screening can also lead to what might be perceived as risky treatments.[23, 24] Emphasising how CRC screening can reduce risk of future ill-health, e.g., by increasing survival rates and reducing the need for more invasive and uncertain cancer treatments, may encourage risk averse individuals to participate in CRC screening.[23]

The current study found some evidence that higher cognitive ability was associated with increased rates of screening. Completing a home CRC screening test may be a cognitively demanding task, and therefore individuals with lower cognitive ability might struggle to successfully complete this task. Gale et al.[15] also found some support that higher cognitive ability predicted participation in a home CRC screening test. Similar to the current study, the association between cognitive ability and CRC screening was attenuated and non-significant when concurrently measuring other psychological variables (conscientiousness, health literacy) and indicators of socioeconomic status (household wealth).[15] These results

For peer review only - http://bmjopen.bmj.com/site/about/guidelines.xhtml

suggest that the association between cognitive ability and CRC screening participation may not be independent of other psychological and socioeconomic variables.

Whereas other studies have found that being future-oriented and more health literate are associated with an increased likelihood of participating in cancer screening,[18, 22, 23, 38] the present study found that present-orientation and health literacy associations were attenuated and non-significant when concurrently measuring other psychological variables. Present-orientation and health literacy may not have associations with CRC screening that are independent of these other psychological characteristics. Conscientiousness, which has been linked to increased colorectal and other cancer screening in previous research,[14, 15] did not predict CRC screening here. One limitation of the current study is that some of the measures had high levels of missing data. One-third of participants did not return the self-completion questionnaire, which included the personality assessment. Conscientiousness is characterised by being organised and industrious,[35] therefore individuals who returned the questionnaire may score higher on conscientiousness than those who did not.

This study examined the association between a range of different psychological characteristics with CRC screening, but it did not consider the four key constructs (perceived susceptibility, severity, benefits and barriers) of the Health Belief Model. In addition to these constructs, the Health Belief Model assumes that modifying factors including psychological and socioeconomic variables can influence health behaviour.[10] These modifying factors may be mediators or moderators of the association between the Health Belief Model constructs and health behaviours.[10, 25] Research should be carried out to explore the role that these psychological variables—especially risk aversion—play in the association between the Health Belief Model constructs and CRC screening participation.

For peer review only - http://bmjopen.bmj.com/site/about/guidelines.xhtml

This study has a number of advantages, including that the HAGIS pilot study was designed to be representative of Scottish adults aged over 50 years. However, only 42% of the analytic sample had data on all variables of interest. Participants with complete data on all variables were more likely to be cohabiting, have higher qualifications and live in less deprived areas, therefore a limitation is that the sample used in the fully-adjusted model is not representative of the Scottish population. The wide range of psychological characteristics assessed in the HAGIS study is an advantage. This enabled the present study examine the role of each psychological characteristic on CRC screening when considered individually, and concurrently with other psychological measures. A limitation is that this study examined whether participants have ever screened, and not whether they regularly take part in CRC screening, which is important for detecting cancer early.[3] Future studies should investigate the psychological correlates of regularly participating in CRC screening.

In this sample of Scottish middle-aged and older adults, individuals who were risk averse were less likely to self-report having ever completed a home CRC screening test, even when adjusting for a range of other psychological and socioeconomic variables. Time preference, health literacy, cognitive ability and personality did not have associations with CRC screening participation independent of the other psychological characteristics measured. Educational materials that emphasise how CRC screening can reduce the risk of future ill-health may encourage risk averse individuals to participate in CRC screening.

For peer review only

Table 1 Participant characteristics according to colorectal cancer screening participation status

	N	Yes (n=683)	No (n=225)	Effect size	p value for a difference
Age (years), mean (SD)	908	66.51 (7.95)	63.87 (8.76)	r=0.136	<0.001
Sex, n (%)	908			Φ=0.011	0.741
Female		382 (55.9%)	123 (54.7%)		
Male		301 (44.1%)	102 (45.3%)		
Living Arrangement, n (%)	908			Φ=0.122	<0.001
Cohabiting		474 (69.4%)	126 (56.0%)		
Living alone		209 (30.6%)	99 (44.0%)		
Qualifications, n (%)	902			Φ_c=0.123	0.009
Primary or less		117 (17.3%)	50 (22.3%)		
O level/O grade		194 (28.6%)	81 (36.2%)		
Highers/Sixth year studies/HNC/HND		208 (30.7%)	53 (23.6%)		
First degree		90 (13.3%)	29 (12.9%)		
Postgraduate/higher degree		69 (10.2%)	11 (4.9%)		
SIMD, n (%)	908			Φ=0.163	<0.001
1 (Most deprived)		139 (20.4%)	73 (32.4%)		
2		136 (19.9%)	59 (26.2%)		
3		213 (31.2%)	50 (22.2%)		
4 (Least deprived)		195 (28.6%)	43 (19.1%)		
Risk aversion, n (%)	581				
1 (Risk seeking)		47 (10.3%)	8 (6.5%)		
2		46 (10.1%)	6 (4.8%)		
3		55 (12.0%)	12 (9.7%)		
4 (Risk averse)		309 (67.6%)	98 (79.0%)		
Risk aversion, mean (SD)		3.37 (1.03)	3.61 (0.85)	r=0.104	0.012
Present-orientation, n (%)	504				
1 (Future-oriented)		159 (39.7%)	24 (23.3%)		
2		27 (6.7%)	4 (3.9%)		
3		20 (5.0%)	2 (1.9%)		
4		34 (8.5%)	8 (7.8%)		
5		20 (5.0%)	3 (2.9%)		
6		17 (4.2%)	15 (14.6%)		
7		44 (11.0%)	21 (20.4%)		
8 (Present-oriented)		80 (20.0%)	26 (25.2%)		
Present orientation, mean (SD)		3.89 (2.89)	5.15 (2.74)	r=0.159	<0.001
Health literacy, mean (SD)	867	4.51 (1.68)	4.07 (1.86)	r=0.103	0.002
Cognitive ability, mean (SD)	790	0.06 (0.98)	-0.21 (1.06)	g=0.273	0.001
Extraversion, mean (SD)	575	30.64 (7.17)	30.71 (7.17)	g=0.009	0.929
Agreeableness, mean (SD)	566	40.18 (5.95)	39.88 (6.40)	r=0.010	0.804
Conscientiousness, mean (SD)	547	37.77 (6.04)	36.59 (6.63)	g=0.191	0.065
Emotional stability, mean (SD)	575	33.68 (7.22)	31.99 (7.97)	g=0.229	0.035
Intellect, mean (SD)	565	33.53 (5.61)	32.14 (5.91)	r=0.112	0.008

SIMD, Scottish Index of Multiple Deprivation; r, effect size correlation coefficient; Φ, Phi; Φ_c, Cramer's V; g, Hedge's g.

For peer review only - http://bmjopen.bmj.com/site/about/guidelines.xhtml

Table 2 Odd ratios (95% CI) for participating in colorectal cancer screening

	Model 1		Model 2	
	n	OR (95% CI)	n	OR (95% CI)
Risk aversion	581	0.66 (0.51 to 0.85)**	577	0.71 (0.55 to 0.91)*
Present-orientation	504	0.86 (0.80 to 0.94)**	500	0.91 (0.83 to 0.99)
Health literacy	867	1.20 (1.09 to 1.31)**	861	1.17 (1.06 to 1.28)**
Cognitive ability	790	1.51 (1.25 to 1.81)***	784	1.36 (1.10 to 1.67)*
Extraversion	575	0.99 (0.96 to 1.02)	571	0.99 (0.96 to 1.02)
Agreeableness	566	1.01 (0.97 to 1.04)	562	1.01 (0.97 to 1.04)
Conscientiousness	547	1.03 (1.00 to 1.07)	543	1.03 (0.99 to 1.07)
Emotional stability	575	1.03 (1.00 to 1.06)	571	1.02 (0.99 to 1.05)
Intellect	565	1.05 (1.01 to 1.09)*	561	1.03 (0.99 to 1.07)

Model 1 reports the ORs (95% CIs) for each psychological variable entered individually, adjusting for age, age-squared, sex, living arrangement and sex*living arrangement. Model 2 reports the ORs (95% CIs) when additionally adjusting for deprivation and qualifications. *p<0.05, **p<0.01, ***p<0.001. P-values are false discovery rate corrected.

For peer review only

For peer review only - http://bmjopen.bmj.com/site/about/guidelines.xhtml

Table 3 Odd ratios (95% CIs) for participating in colorectal cancer screening

	Model 1 (n=388)	Model 2 (n=384)	Model 3 (n=384)
Risk aversion	0.52 (0.36 to 0.76)**	0.53 (0.36 to 0.77)**	0.55 (0.39 to 0.78)**
Present-orientation	0.94 (0.85 to 1.04)	0.98 (0.88 to 1.08)	-
Health literacy	1.02 (0.84 to 1.24)	1.02 (0.84 to 1.25)	-
Cognitive ability	1.38 (0.97 to 1.97)	1.26 (0.86 to 1.85)	1.42 (1.05 to 1.93)
Extraversion	0.97 (0.92 to 1.01)	0.97 (0.92 to 1.01)	-
Agreeableness	0.95 (0.90 to 1.02)	0.96 (0.90 to 1.03)	-
Conscientiousness	1.04 (0.99 to 1.10)	1.04 (0.98 to 1.10)	-
Emotional stability	1.03 (0.98 to 1.07)	1.02 (0.98 to 1.07)	-
Intellect	1.02 (0.96 to 1.08)	1.01 (0.95 to 1.08)	-
Age	1.08 (1.04 to 1.12)***	1.08 (1.04 to 1.12)**	1.09 (1.05 to 1.12)***
Age^2	0.99 (0.99 to 0.99)**	0.99 (0.99 to 0.99)*	0.995 (0.991 to 0.999)*
Sex			
Female	Reference	Reference	-
Male	0.60 (0.32 to 1.11)	0.61 (0.33 to 1.15)	-
Living arrangement			
Cohabiting	Reference	Reference	Reference
Living alone	0.40 (0.22 to 0.73)*	0.43 (0.23 to 0.78)*	0.54 (0.31 to 0.95)
Sex*Living arrangement	0.24 (0.07 to 0.81)	0.24 (0.07 to 0.81)	-
Qualifications	-	1.06 (0.78 to 1.45)	-
SIMD	-	1.40 (1.05 to 1.87)	1.47 (1.05 to 1.92)*

Model 1 reports the ORs (95% CIs) for each psychological variable entered simultaneously in the same model, adjusting for age, age-squared, sex, living arrangement and sex*living arrangement. Model 2 reports the ORs (95% CIs) when additionally adjusting for deprivation and qualifications. Model 3 reports the results of a backward elimination model using all variables entered in the fully-adjusted model (Model 2).
Age^2, Age-squared; SIMD, Scottish Index of Multiple Deprivation. Higher scores on SIMD reflect less deprivation.
*p<0.05, **p<0.01, ***p<0.001. P-values are false discovery rate corrected.

For peer review only - http://bmjopen.bmj.com/site/about/guidelines.xhtml

Figure 1 Participant flow chart

For peer review only

For peer review only - http://bmjopen.bmj.com/site/about/guidelines.xhtml

Acknowledgements We thank the HAGIS participants.

Completing Interests None.

Funding This work was supported by the University of Edinburgh Centre for Cognitive Ageing and Cognitive Epidemiology, part of the cross council Lifelong Health and Wellbeing Initiative, funded by the Biotechnology and Biological Sciences Research Council (BBSRC) and Medical Research Council (MRC) (grant number: MR/K026992/1). The HAGIS study (data resource) was part-funded by the National Institute of Aging (grant number: RAG044535A) in the USA and was also part-funded by the Nuffield Foundation (grant number: OPD/42197), but the views expressed are those of the authors and not necessarily those of the Foundation. MvdP is supported by the Chief Scientist Office (CSO) of the Scottish Government Health and Social Care Directorates (SGHSC; grant number: not applicable). The views expressed here are those of the Unit and not necessarily those of the CSO.

Author Contributions Chloe Fawns-Ritchie contributed to the conception and design of the project, analysed the data, interpreted the data, and drafted the initial manuscript. Christopher B. Miller contributed to the conception and design of the project, analysed the data, interpreted the data, and drafted the initial manuscript. Marjon van der Pol contributed to the statistical analysis of the data, interpreted the data, and critically revised the manuscript. Elaine Douglas interpreted the data, and critically revised the manuscript. David Bell interpreted the data, and critically revised the manuscript. Ronan E. O'Carroll contributed to the conception and design of the project, interpreted the data, and critically revised the manuscript. Ian J. Deary contributed to the conception and design of the project, interpreted the data, and critically revised the manuscript.

For peer review only

Data Availability Statement The anonymised HAGIS pilot data will be made available via the Gateway to Global Aging Data: https://g2aging.org/

Ethics Statement Ethical approval was obtained from the University of Stirling Ethics, the Administrative Data Research Network (PROJ-065-HAGIS Study), and the Public Benefits and Privacy Panel (HAGIS 1516-0417).

For peer review only

For peer review only - http://bmjopen.bmj.com/site/about/guidelines.xhtml

REFERENCES

1 NHS Information Services Division. Cancer Incidence in Scotland (2016). Scotland: NHS Nationl Services Scotland; 2018. Retrieved from: https://www.isdscotland.org/Health-Topics/Cancer/Publications/2018-04-24/2018-04-24-Cancer-Incidence-Report.pdf.

2 Scholefield J, Moss S, Mangham C, Whynes D, Hardcastle J. Nottingham trial of faecal occult blood testing for colorectal cancer: a 20-year follow-up. *Gut* 2012;61(7):1036-40.

3 NHS Information Services Division. Scottish Bowel Screening Programme Statistics. Scotland: NHS National Services Scotland; 2017. Retrieved from: http://www.nsd.scot.nhs.uk/services/screening/bowelscreening/index.html.

4 Mansouri D, McMillan DC, Grant Y, Crighton EM, Horgan PG. The impact of age, sex and socioeconomic deprivation on outcomes in a colorectal cancer screening programme. *PLoS One* 2013;8(6):e66063.

5 Quyn AJ, Fraser CG, Stanners G, Carey FA, Carden C, Shaukat A, et al. Uptake trends in the Scottish Bowel Screening Programme and the influences of age, sex, and deprivation. *J Med Screen* 2017:969141317694065.

6 Rosenstock IM. Historical origins of the health belief model. *Health Education Monographs* 1974;2(4):328-35.

7 Green EC, Murphy EM, Gryboski K. The Health Belief Model. The Wiley Encyclopedia of Health Psychology2020. p. 211-4.

8 Carpenter CJ. A meta-analysis of the effectiveness of health belief model variables in predicting behavior. *Health Communications* 2010;25(8):661-9.

9 Janz NK, Wren PA, Schottenfeld D, Guire KE. Colorectal cancer screening attitudes and behavior: a population-based study. *Preventive Medicine* 2003;37(6):627-34.

For peer review only

10 Tanner-Smith EE, Brown TN. Evaluating the Health Belief Model: A critical review of studies predicting mammographic and pap screening. *Social Theory & Health* 2010;8(1):95-125.

11 Heflin MT, Oddone EZ, Pieper CF, Burchett BM, Cohen HJ. The effect of comorbid illness on receipt of cancer screening by older people. *J Am Geriatr Soc* 2002;50(10):1651-8.

12 Leung DY, Leung AY, Chi I. Breast and colorectal cancer screening and associated correlates among Chinese older women. *Asian Pac J Cancer Prev* 2012;13(1):283-7.

13 Arai S, Nakaya N, Kakizaki M, Ohmori-Matsuda K, Shimazu T, Kuriyama S, et al. Personality and gastric cancer screening attendance: a cross-sectional analysis from the Miyagi Cohort Study. *J Epidemiol* 2009;19(1):34-40.

14 Sen CK, Kumkale GT. Who does not get screened? A simple model of the complex relationships in mammogram non-attendance. *J Health Psychol* 2016;21(12):2838-50.

15 Gale CR, Deary IJ, Wardle J, Zaninotto P, Batty GD. Cognitive ability and personality as predictors of participation in a national colorectal cancer screening programme: the English Longitudinal Study of Ageing. *J Epidemiol Community Health* 2015;69(6):530-5.

16 Baker DW. The meaning and the measure of health literacy. *J Gen Intern Med* 2006;21(8):878-83.

17 Kobayashi LC, Wardle J, von Wagner C. Limited health literacy is a barrier to colorectal cancer screening in England: evidence from the English Longitudinal Study of Ageing. *Prev Med* 2014;61:100-5.

18 Solmi F, Von Wagner C, Kobayashi LC, Raine R, Wardle J, Morris S. Decomposing socio-economic inequality in colorectal cancer screening uptake in England. *Soc Sci Med* 2015;134:76-86.

For peer review only - http://bmjopen.bmj.com/site/about/guidelines.xhtml

19 Peterson NB, Dwyer KA, Mulvaney SA, Dietrich MS, Rothman RL. The influence of health literacy on colorectal cancer screening knowledge, beliefs and behavior. *J Natl Med Assoc* 2007;99(10):1105-12.

20 Oldach BR, Katz ML. Health literacy and cancer screening: a systematic review. *Patient education and counseling* 2014;94(2):149-57.

21 Bradford WD. The association between individual time preferences and health maintenance habits. *Med Decis Making* 2010;30(1):99-112.

22 Bradford WD, Zoller J, Silvestri GA. Estimating the effect of individual time preferences on the use of disease screening. *Southern Economic Journal* 2010;76(4):1005-31.

23 Goldzahl L. Contributions of risk preference, time orientation and perceptions to breast cancer screening regularity. *Soc Sci Med* 2017;185:147-57.

24 Picone G, Sloan F, Taylor D. Effects of risk and time preference and expected longevity on demand for medical tests. *J Risk Uncertain* 2004;28(1):39-53.

25 Whitaker KL, Good A, Miles A, Robb K, Wardle J, von Wagner C. Socioeconomic inequalities in colorectal cancer screening uptake: does time perspective play a role? *Health Psychol* 2011;30(6):702-9.

26 Lo SH, Waller J, Wardle J, von Wagner C. Comparing barriers to colorectal cancer screening with barriers to breast and cervical screening: a population-based survey of screening-age women in Great Britain. *J Med Screen* 2013;20(2):73-9.

27 Reeve CL, Basalik D. Is health literacy an example of construct proliferation? A conceptual and empirical evaluation of its redundancy with general cognitive ability. *Intelligence* 2014;44:93-102.

28 Shamosh NA, Gray JR. Delay discounting and intelligence: A meta-analysis. *Intelligence* 2008;36(4):289-305.

For peer review only - http://bmjopen.bmj.com/site/about/guidelines.xhtml

29 Boyle PA, Yu L, Wilson RS, Segawa E, Buchman AS, Bennett DA. Cognitive decline impairs financial and health literacy among community-based older persons without dementia. *Psychology and aging* 2013;28(3):614-24.

30 Becker A, Deckers T, Dohmen T, Falk A, Kosse F. The relationship between economic preferences and psychological personality measures. *Annu Rev Econom* 2012;4(1):453-78.

31 Douglas E, Rutherford A, Bell D. Pilot study protocol to inform a future longitudinal study of ageing using linked administrative data: Healthy AGeing in Scotland (HAGIS). *BMJ Open* 2018;8(1):e018802.

32 Andersen S, Harrison GW, Lau MI, Rutström EE. Elicitation using multiple price list formats. *Exp Econ* 2006;9(4):383-405.

33 Kirby KN, Petry NM, Bickel WK. Heroin addicts have higher discount rates for delayed rewards than non-drug-using controls. *Journal of Experimental psychology: general* 1999;128(1):78.

34 Weiss BD, Mays MZ, Martz W, Castro KM, DeWalt DA, Pignone MP, et al. Quick assessment of literacy in primary care: the newest vital sign. *Ann Fam Med* 2005;3(6):514-22.

35 Goldberg LR. The development of markers for the Big-Five factor structure. *Psychol Assess* 1992;4(1):26.

36 Scottish Government. Scottish Index of Multiple Deprivation 2016. Scotland; 2016.

37 Bell D, Douglas E. Bowel cancer screening uptake in a Scottish population. Stirling: University of Stirling; 2017. Retrieved from: https://www.hagis.scot/publications/.

38 Arnold CL, Rademaker A, Bailey SC, Esparza JM, Reynolds C, Liu D, et al. Literacy barriers to colorectal cancer screening in community clinics. *J Health Commun* 2012;17(sup3):252-64.

For peer review only - http://bmjopen.bmj.com/site/about/guidelines.xhtml

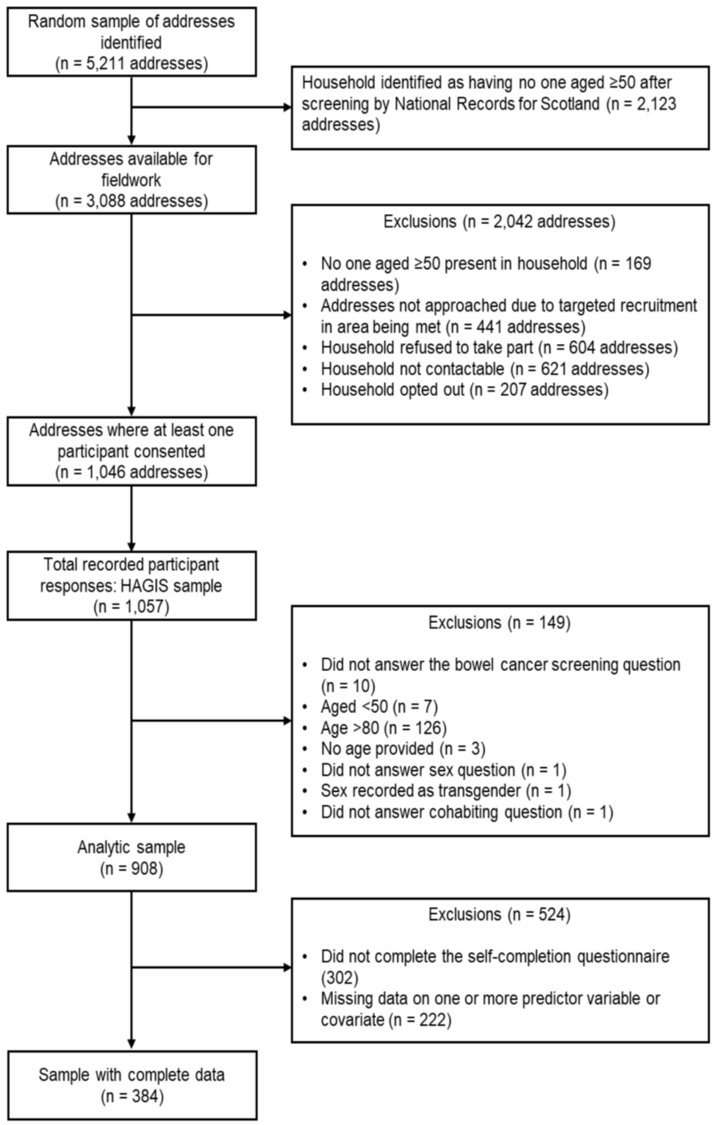

Figure 1: Participant flow chart

209x297mm (300 x 300 DPI)

Supplementary Materials for: Fawns-Ritchie et al. Psychological correlates of free colorectal cancer screening uptake in a Scottish sample

HAGIS risk aversion questions

1. Income A, which will with certainty give you a £1,500 per month for the rest of your life OR Income B which will give you a 50-50 chance of £3,000 and a 50-50 chance of £1,000 per month for the rest of your life.

Would you choose Income A or Income B?

(a) Income A
(b) Income B

2. Income A, which will with certainty give you a £1,500 per month for the rest of your life OR Income B, which will give you a 50-50 chance of £3,000 and a 50-50 chance of £1,200 per month for the rest of your life.

Would you choose Income A or Income B?

(a) Income A
(b) Income B

3. Income A, which will with certainty give you a £1,500 per month for the rest of your life OR Income B, which will give you a 50-50 chance of £3,000 and a 50-50 chance of £1,300 per month for the rest of your life.

Would you choose Income A or Income B?

(a) Income A
(b) Income B

For peer review only

For peer review only - http://bmjopen.bmj.com/site/about/guidelines.xhtml

HAGIS time orientation questions

1. Would you rather have £1,500 now or £1,506 a month from now?
   (a) £1,500 now
   (b) £1,506 a month from now

2. Would you rather have £1,500 now or £1,512 a month from now?
   (a) £1,500 now
   (b) £1,512 a month from now

3. Would you rather have £1,500 now or £1,518 a month from now?
   (a) £1,500 now
   (b) £1,518 a month from now

4. Would you rather have £1,500 now or £1,524 a month from now?
   (a) £1,500 now
   (b) £1,524 a month from now

5. Would you rather have £1,500 now or £1,536 a month from now?
   (a) £1,500 now
   (b) £1,536 a month from now

6. Would you rather have £1,500 now or £1,548 a month from now?
   (a) £1,500 now
   (b) £1,548 a month from now

7. Would you rather have £1,500 now or £1,596 a month from now?
   (a) £1,500 now
   (b) £1,596 a month from now

For peer review only - http://bmjopen.bmj.com/site/about/guidelines.xhtml

SUPPLEMENTARY METHODS

Cognitive ability

Five tests of cognitive function that were assessed during the face-to-face interview were used here. Verbal declarative memory was assessed using the word list learning test in which participants were presented with a list of 10 words and had to recall these words immediately, and again after an approximately 20 minute delay. The scores for word list learning immediate and delayed tests correlated at $r=0.66$ ($p<0.001$) therefore, the score used here is the total number of words recalled across the two tests (range 0-20). Categorical (animal) fluency, in which participants are asked to name as many animals as possible, was used to measure executive function. The score is the number of animals named in 60 seconds. The word list learning and animal fluency tests have been administered in other longitudinal studies of ageing including the Health and Retirement Study[1] and the English Longitudinal Study of Ageing.[2] Processing speed was measured using the letter digit substitution test.[3] Here, participants were presented with a piece of paper with a key pairing letters with numbers. Below the key were rows of letters with empty boxes underneath each letter. Participants were to use this key to write in the number that is paired with each letter. The score is the number of correct letter-digit matches made in 60 seconds. Two tests from the COGNITO neuropsychological test battery were administered to assess vocabulary and non-verbal fluid reasoning.[4] A shortened version of the COGNITO vocabulary test[4] was used. Participants were presented with a target word and were instructed to select which of six alternatives means the same as the target word. There were 20 items and the score is the number of correctly answered questions. COGNITO matrices[4] was used to assess non-verbal reasoning. Participants were presented with 15 matrix designs with a piece missing

For peer review only - http://bmjopen.bmj.com/site/about/guidelines.xhtml

and they were required to select the missing piece from a list of alternatives. The score is the number of correctly answered questions in 4 minutes.

To create a measure of general cognitive ability, scores on the five cognitive tests were entered into a principal components analysis. The eigenvalues and scree plot (Supplementary Figure 1) indicated that one component should be extracted. The first unrotated principal component accounted for 44% of the total variance across the five tests. The loadings for the five tests were: total word recall=0.60, animal fluency=0.73, letter digit substitution=0.70, vocabulary=0.58, matrices=0.68. The score from the first unrotated principal component was used here as a measure of general cognitive ability (mean=0.00, SD=1.00).

For peer review only

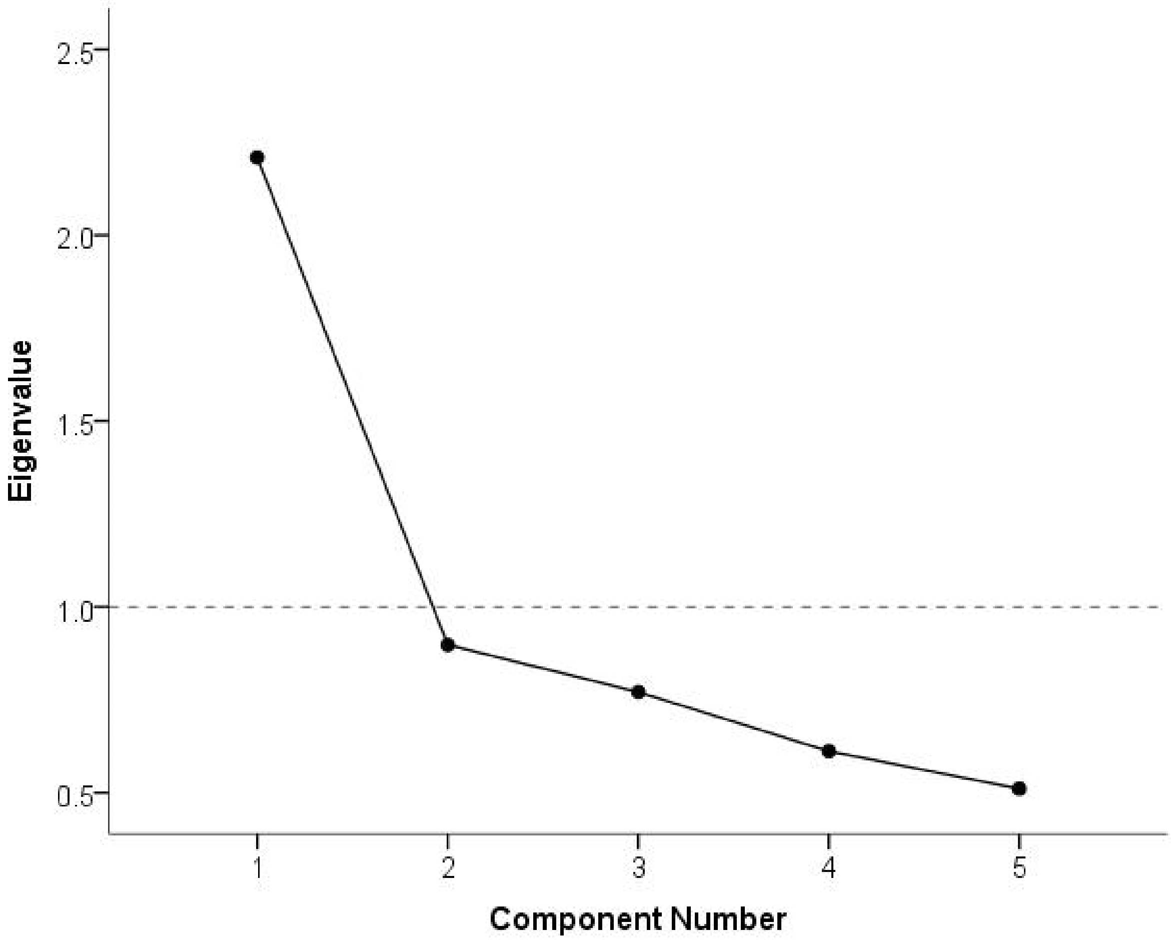

Supplementary Figure 1 Scree plot of eigenvalues from the 5 HAGIS cognitive tests

For peer review only - http://bmjopen.bmj.com/site/about/guidelines.xhtml

Supplementary Table 1 Pearson (below the diagonal) and rank-order (above the diagonal) correlations between psychological variables and covariates

	1	2	3	4	5	6	7	8	9	10	11	12	13	14
1. Age	-	0.04	-0.04	-0.16***	0.03	0.18***	-0.01	-0.20***	-0.28***	-0.03	-0.08	-0.05	0.06	-0.08
2. Sex	0.04	-	0.18***	0.07*	0.03	-0.08	-0.11*	-0.04	-0.04	-0.04	-0.29***	-0.09*	0.05	0.06
3. Cohabiting	-0.04	0.18***	-	0.14***	0.17***	-0.10*	-0.11*	0.17***	0.15***	0.11**	0.01	0.06	0.05	0.14**
4. Qualifications	-0.14***	0.08*	0.14***	-	0.35***	-0.21***	-0.24***	0.22***	0.48***	0.00	0.03	0.09*	0.13**	0.36***
5. SIMD	0.02	0.03	0.17***	0.35***	-	-0.15***	-0.28***	0.05	0.26***	0.01	0.00	-0.02	0.04	0.13**
6. Risk aversion	0.15***	-0.09*	-0.07	-0.16***	-0.12**	-	0.14**	-0.17***	-0.17***	-0.06	0.07	0.01	0.03	-0.18***
7. Present-orientation	-0.01	-0.10*	-0.11*	-0.25***	-0.30***	0.14**	-	-0.11*	-0.11***	0.07	0.10*	0.04	0.03	-0.12**
8. Health literacy	-0.21***	-0.06	0.16***	0.22***	0.08*	-0.12**	-0.09	-	0.45***	0.11**	0.08	0.13**	0.08	0.22
9. Cognitive ability	-0.27***	-0.04	0.15***	0.49***	0.27***	-0.13**	-0.11*	0.46***	-	0.17***	0.22***	0.23***	0.18***	0.39***
10. Extraversion	-0.02	-0.05	0.11**	-0.02	0.01	-0.05	0.07	0.11**	0.14**	-	0.37***	0.20***	0.27***	0.28***
11. Agreeableness	-0.09*	-0.30***	0.02	0.03	0.01	0.11*	0.10*	0.13**	0.22***	0.35***	-	0.45***	0.25***	0.27***
12. Conscientiousness	-0.05	-0.09*	0.07	0.09*	-0.01	0.05	0.04	0.15**	0.23***	0.18***	0.44***	-	0.30***	0.30***
13. Emotional stability	0.07	0.05	0.05	0.15***	0.06	0.03	0.01	0.08	0.18***	0.28***	0.22***	0.29***	-	0.08
14. Intellect	-0.06	0.08	0.12**	0.39***	0.14**	-0.15***	-0.13**	0.26***	0.38***	0.27***	0.25	0.30***	0.08	-

SIMD, Scottish Index of Multiple Deprivation.

Sex is coded 0 for female, 1 for male. Cohabiting is coded 0 for living alone, 1 for cohabiting.

*p<0.05, **p<0.01, ***p<0.001.

For peer review only

For peer review only - http://bmjopen.bmj.com/site/about/guidelines.xhtml

Supplementary Table 2 Partial correlations controlling for age and sex between psychological variables

	1	2	3	4	5	6	7	8	9
1. Risk aversion	-								
2. Present-orientation	0.15**	-							
3. Health literacy	-0.18***	-0.11*	-						
4. Cognitive ability	-0.12*	-0.16**	0.44***	-					
5. Extraversion	-0.01	0.12*	0.06	0.14**	-				
6. Agreeableness	0.09	0.09	-0.01	0.13*	0.37***	-			
7. Conscientiousness	0.05	0.06	0.06	0.20***	0.22***	0.45***	-		
8. Emotional stability	0.03	0.03	0.1	0.23***	0.27***	0.30***	0.33***	-	
9. Intellect	-0.15**	-0.13**	0.23***	0.37***	0.27***	0.27***	0.21***	0.09	-

For peer review only

Supplementary Table 3 Participant characteristics according to whether they have complete data on all variables of interest (completers) or whether they had missing data (non-completers)

	N	Completers (n=384)	Non-completers (n=524)	p value for a difference
Age (years), mean (SD)	908	65.33 (8.10)	66.24 (8.31)	0.101
Sex, n (%)	908			0.953
Female		214 (55.7%)	291 (55.5%)	
Male		170 (44.3%)	233 (44.5%)	
Living Arrangement, n (%)	908			0.003
Cohabiting		275 (71.6%)	325 (62.0%)	
Living alone		109 (28.4%)	199 (38.0%)	
Qualifications, n (%)	902			<0.001
Primary or less		43 (11.2%)	124 (23.9%)	
O level/O grade		113 (29.4%)	162 (31.3%)	
Highers/Sixth year studies/HNC/HND		113 (29.4%)	148 (28.6%)	
First degree		62 (16.1%)	57 (11.0%)	
Postgraduate/higher degree		53 (13.8%)	27 (5.2%)	
SIMD, n (%)	908			0.007
1 (Most deprived)		68 (17.7%)	144 (27.5%)	
2		86 (22.4%)	109 (20.8%)	
3		121 (31.5%)	142 (27.1%)	
4 (Least deprived)		109 (28.4%)	129 (24.6%)	

SIMD, Scottish Index of Multiple Deprivation.

For peer review only

Supplementary Table 4 Odd ratios (95% CIs) for participating in colorectal cancer screening using participants with complete data (n=384)

	Model 1	Model 2
	OR (95% CI)	OR (95% CI)
Risk aversion	0.51 (0.35 to 0.72)***	0.53 (0.37 to 0.77)**
Present-orientation	0.90 (0.82 to 0.98)*	0.96 (0.87 to 1.06)
Health literacy	1.17 (0.99 to 1.37)	1.10 (0.93 to 1.31)
Cognitive ability	1.56 (1.17 to 2.08)**	1.32 (0.96 to 1.83)
Extraversion	0.98 (0.94 to 1.01)	0.98 (0.95 to 1.02)
Agreeableness	0.97 (0.92 to 1.02)	0.98 (0.93 to 1.03)
Conscientiousness	1.02 (0.98 to 1.06)	1.02 (0.98 to 1.07)
Emotional stability	1.02 (0.98 to 1.06)	1.01 (0.98 to 1.05)
Intellect	1.05 (1.00 to 1.10)	1.02 (0.97 to 1.08)

Model 1 reports the ORs (95% CIs) for each psychological variable entered individually, adjusting for age, age-squared, sex, living arrangement and sex*living arrangement. Model 2 reports the ORs (95% CIs) when additionally adjusting for deprivation and qualifications. *p<0.05, **p<0.01, ***p<0.001.

For peer review only

SUPPLEMENTARY REFERENCES

1 Sonnega A, Faul JD, Ofstedal MB*, et al.* Cohort Profile: the Health and Retirement Study (HRS). *International journal of epidemiology* 2014;**43**:576-85. doi: 10.1093/ije/dyu067

2 Steptoe A, Breeze E, Banks J*, et al.* Cohort profile: the English longitudinal study of ageing. *International journal of epidemiology* 2013;**42**:1640-8. doi: 10.1093/ije/dys168

3 van der Elst W, van Boxtel MP, van Breukelen GJ*, et al.* The Letter Digit Substitution Test: normative data for 1,858 healthy participants aged 24–81 from the Maastricht Aging Study (MAAS): influence of age, education, and sex. *J Clin Exp Neuropsychol* 2006;**28**:998-1009. doi: 10.1080/13803390591004428

4 Ritchie K, de Roquefeuil G, Ritchie C*, et al.* COGNITO: computerized assessment of information processing. *J Psychol Psychother* 2014;**4**:136. doi: 10.4172/2161-0487.1000136

For peer review only

For peer review only - http://bmjopen.bmj.com/site/about/guidelines.xhtml

STROBE Statement—Checklist of items that should be included in reports of ***cross-sectional studies***

	Item No	Recommendation	Page No
**Title and abstract**	1	(*a*) Indicate the study's design with a commonly used term in the title or the abstract	1-2
		(*b*) Provide in the abstract an informative and balanced summary of what was done and what was found	2
**Introduction**			
Background/rationale	2	Explain the scientific background and rationale for the investigation being reported	5-8
Objectives	3	State specific objectives, including any prespecified hypotheses	8
**Methods**			
Study design	4	Present key elements of study design early in the paper	8, 13-15
Setting	5	Describe the setting, locations, and relevant dates, including periods of recruitment, exposure, follow-up, and data collection	8-9
Participants	6	(*a*) Give the eligibility criteria, and the sources and methods of selection of participants	8-9
Variables	7	Clearly define all outcomes, exposures, predictors, potential confounders, and effect modifiers. Give diagnostic criteria, if applicable	10-13
Data sources/ measurement	8*	For each variable of interest, give sources of data and details of methods of assessment (measurement). Describe comparability of assessment methods if there is more than one group	10-13
Bias	9	Describe any efforts to address potential sources of bias	17-18
Study size	10	Explain how the study size was arrived at	8-9, 15
Quantitative variables	11	Explain how quantitative variables were handled in the analyses. If applicable, describe which groupings were chosen and why	13-15
Statistical methods	12	(*a*) Describe all statistical methods, including those used to control for confounding	13-15
		(*b*) Describe any methods used to examine subgroups and interactions	13-15
		(*c*) Explain how missing data were addressed	14-15
		(*d*) If applicable, describe analytical methods taking account of sampling strategy	NA
		(*e*) Describe any sensitivity analyses	13-15
**Results**			
Participants	13*	(a) Report numbers of individuals at each stage of study—eg numbers potentially eligible, examined for eligibility, confirmed eligible, included in the study, completing follow-up, and analysed	8-9, 15, Fig1
		(b) Give reasons for non-participation at each stage	8-9, 15
		(c) Consider use of a flow diagram	Fig1
Descriptive data	14*	(a) Give characteristics of study participants (eg demographic, clinical, social) and information on exposures and potential confounders	22
		(b) Indicate number of participants with missing data for each variable of interest	22
Outcome data	15*	Report numbers of outcome events or summary measures	22

For peer review only - http://bmjopen.bmj.com/site/about/guidelines.xhtml

Main results	16	(*a*) Give unadjusted estimates and, if applicable, confounder-adjusted estimates and their precision (eg, 95% confidence interval). Make clear which confounders were adjusted for and why they were included	23-24
		(*b*) Report category boundaries when continuous variables were categorized	13-15
		(*c*) If relevant, consider translating estimates of relative risk into absolute risk for a meaningful time period	NA
Other analyses	17	Report other analyses done—eg analyses of subgroups and interactions, and sensitivity analyses	17-18
**Discussion**			
Key results	18	Summarise key results with reference to study objectives	18-19
Limitations	19	Discuss limitations of the study, taking into account sources of potential bias or imprecision. Discuss both direction and magnitude of any potential bias	20-21
Interpretation	20	Give a cautious overall interpretation of results considering objectives, limitations, multiplicity of analyses, results from similar studies, and other relevant evidence	18-21
Generalisability	21	Discuss the generalisability (external validity) of the study results	18-21
**Other information**			
Funding	22	Give the source of funding and the role of the funders for the present study and, if applicable, for the original study on which the present article is based	26

*Give information separately for exposed and unexposed groups.

Note: An Explanation and Elaboration article discusses each checklist item and gives methodological background and published examples of transparent reporting. The STROBE checklist is best used in conjunction with this article (freely available on the Web sites of PLoS Medicine at http://www.plosmedicine.org/, Annals of Internal Medicine at http://www.annals.org/, and Epidemiology at http://www.epidem.com/). Information on the STROBE Initiative is available at www.strobe-statement.org.

For peer review only

For peer review only - http://bmjopen.bmj.com/site/about/guidelines.xhtml
